# Phospholipid Removal for Enhanced Chemical Exposomics in Human Plasma

**DOI:** 10.1021/acs.est.3c00663

**Published:** 2023-07-03

**Authors:** Kalliroi Sdougkou, Hongyu Xie, Stefano Papazian, Bénilde Bonnefille, Ingvar A. Bergdahl, Jonathan W. Martin

**Affiliations:** † Department of Environmental Science, Science for Life Laboratory, Stockholm University, Stockholm 106 91, Sweden; ‡ National Facility for Exposomics, Science for Life Laboratory, Stockholm University, Solna 171 65, Sweden; § Department of Public Health and Clinical Medicine, Section for Sustainable Health, Umeå University, Umeå 901 87, Sweden

**Keywords:** chemical exposome, high-resolution mass spectrometry, liquid chromatography, multiclass targeted, non-targeted, plasma, phospholipid

## Abstract

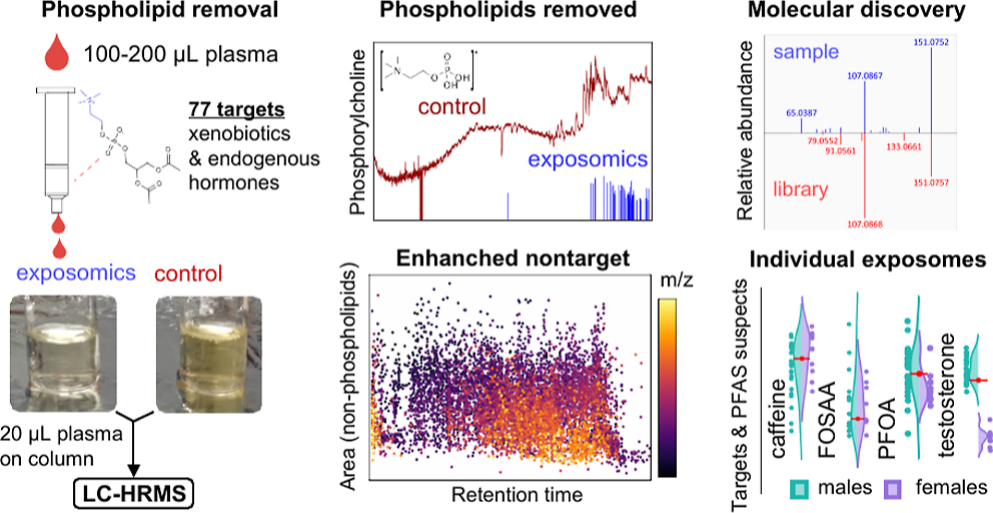

The challenge of chemical exposomics in human plasma is the 1000-fold concentration gap between endogenous substances and environmental pollutants. Phospholipids are the major endogenous small molecules in plasma, thus we validated a chemical exposomics protocol with an optimized phospholipid-removal step prior to targeted and non-targeted liquid chromatography high-resolution mass spectrometry. Increased injection volume with negligible matrix effect permitted sensitive multiclass targeted analysis of 77 priority analytes; median MLOQ = 0.05 ng/mL for 200 μL plasma. In non-targeted acquisition, mean total signal intensities of non-phospholipids were enhanced 6-fold in positive (max 28-fold) and 4-fold in negative mode (max 58-fold) compared to a control method without phospholipid removal. Moreover, 109 and 28% more non-phospholipid molecular features were detected by exposomics in positive and negative mode, respectively, allowing new substances to be annotated that were non-detectable without phospholipid removal. In individual adult plasma (100 μL, *n* = 34), 28 analytes were detected and quantified among 10 chemical classes, and quantitation of per- and polyfluoroalkyl substances (PFAS) was externally validated by independent targeted analysis. Retrospective discovery and semi-quantification of PFAS-precursors was demonstrated, and widespread fenuron exposure is reported in plasma for the first time. The new exposomics method is complementary to metabolomics protocols, relies on open science resources, and can be scaled to support large studies of the exposome.

## Abstract 2

Chemical exposomics in human plasma was enhanced by an optimized phospholipid removal step that increased targeted method sensitivity while also revealing >13,000 new molecular features by LC-HRMS non-targeted acquisition.

## Introduction

The human exposome includes measures of environmental exposures over an individual’s lifespan, encompassing internal and external factors such as pollution, diet, metabolism, and gut microbiota.^1,2^ These factors could influence health and wellness, or contribute to disease risk, but their dynamic nature and breadth make the exposome a practical challenge to implement in human studies where biological samples are limited.^1,2^ As outlined by Rappaport et al., measurement of the internal chemical exposome in blood has great relevance due to the simultaneous presence of endogenous metabolites, dietary substances, drugs, and environmental contaminants.^3^ Nevertheless, the major practical challenge is that blood concentrations of these span 11 orders of magnitude (i.e., from 160 fM to 140 mM), and environmental contaminants are generally present at 1000-fold lower concentrations than other substances.^2−4^ For this reason, environmental chemical exposures have traditionally been measured by sensitive targeted methods, usually one chemical class at a time.^5^ As a result, the cumulative mixture-effect of environmental chemicals on health, and indeed their interaction with other exposomic and genomic factors remains largely unexplored. Considering the hundreds of thousands of commercial chemicals in global use today,^6^ improved methods are needed to support comprehensive targeted and non-targeted chemical exposomics.

Current analytical trends in chemical exposomics include multiclass targeted methods based on liquid chromatography (LC) or gas chromatography (GC) with mass spectrometry, as well as suspect-screening and non-targeted LC- and GC-high resolution mass spectrometry (HRMS).^7−12^ Non-targeted HRMS approaches are common in metabolomics and are promising for chemical exposomics because they allow profiling and quantification of known substances, while also acquiring spectra of unknown molecules that may be unanticipated biomarkers of disease. Most chemical exposomics approaches in human studies have so-far been adapted directly from high-resolution metabolomics,^13,14^ involving only a protein precipitation step and injecting the equivalent of 1–5 μL of plasma on-column.^8,9,11,12^ While these methods have advantages for throughput and unbiased molecular analysis, they do not address the 1,000-fold concentration gap for environmental contaminants in plasma.^3^

One way to improve sensitivity of chemical exposomics is to introduce more environmental analytes on-column, either through preconcentration steps or large-volume injection. In practice, however, this can lead to increased interference and instrumental fouling from major endogenous substances. Phospholipids are the dominant small molecule class in human plasma, represented by an abundant complex mixture of over 2,000 chemical species.^15^ Even with standard LC metabolomics protocols these cause matrix effects, decreased precision, and compromised chromatography.^16−18^ Other abundant plasma lipid classes, such as triacylglycerides and cholesteryl esters are of lesser concern due to their nonpolar nature and lower extraction with polar solvents.^19^ One important metabolomics study showed that plasma phospholipid removal (after protein precipitation) improves the nonlipid metabolite coverage due to decreased matrix effects.^20^ Commercial phospholipid removal technologies show promise for analysis of environmental chemicals in human plasma or serum, and applications already include the targeted analysis of persistent organic pollutants by GC–MS,^21^ and of per- and poly-fluoroalkyl substances (PFAS) by LC–MS.^22^ Moreover, a chemical exposomics study by Chaker et al. recommended their use, concluding that phospholipid removal provides complementary molecular coverage compared to protein-precipitation alone.^10^ Nevertheless, Chaker et al. also reported that multiclass targeted analyte recoveries following phospholipid removal were low and highly variable when using generic phospholipid removal protocols from commercial suppliers,^10^ and quantitative, validated methods are yet to be developed. We hypothesized that an optimized and validated plasma phospholipid-removal protocol could be developed that would minimize matrix effects in chemical exposomics and may permit larger injection volumes to help overcome the method sensitivity challenges first raised by Rappaport et al. in 2014.^3^

Here we report a quantitatively validated sample preparation workflow for small volumes of human plasma (100–200 μL) including an optimized phospholipid removal step with commercial cartridges. Paired with LC-HRMS, removal of phospholipids permitted larger injection volumes with lower matrix effect, resulting in a highly sensitive method for 77 targeted environmental and endogenous analytes. Moreover, the new chemical exposomics protocol enabled enhanced molecular discovery which we demonstrate here by a non-targeted acquisition strategy, as well as by retrospective suspect screening of PFAS precursors.

## Experimental Section

### Selection of Targeted Analytes

A multiclass targeted analyte list of 77 substances was defined a priori to guide method development and for quantitative monitoring with the optimized exposomics method (Table S1). The list was widely representative of LC–MS amenable compounds, with a log *P* ranging from −3.6 to 7.2, and molecular weight spanning 139–714 Da. Selection of anthropogenic contaminants, natural dietary substances, and tobacco markers, or associated transformation products, was constrained by lists of substances routinely monitored in human serum or urine by the US National Health and Nutrition Examination Survey,^23^ and European HBM4EU priority substances,^24^ with preference given to those substances most frequently detectable in each class based on existing data. Five endogenous steroid hormones (estradiol, hydrocortisone, corticosterone, testosterone, and progesterone) were included as potential effect markers of endocrine disruption; associations are not reported in this preliminary work. Three pharmaceuticals (ibuprofen, diclofenac, and paracetamol) were also included in method validation, but concentrations in Swedish plasma were not investigated due to conditions of the ethics permission.

Native standards (purity > 95%) were used for all targeted analytes and for confirmation of compounds later discovered through suspect screening (Table S2). Select isotopically labelled standards (*n* = 35, purity > 95%) were used for several contaminant classes (Table S3).

### Sample Preparation for Exposomics

The chemical exposomics method optimized here, involving phospholipid removal and higher injection volume, was contrasted with a control method without phospholipid removal, as is typical for metabolomics. The methods are hereafter simply termed “exposomics” and “control” and were contrasted by preparing three aliquots of 200 μL pooled Swedish plasma per method. Plasma aliquots were first placed in 2 mL propylene tubes (Eppendorf) and fortified with 10 μL of isotopically labelled internal standard mixture of 34 substances in methanol (MeOH) (Optima LC/MS Grade, Thermo Scientific) (Table S3, final concentration 1 ng/mL of each). Protein precipitation was achieved by adding 800 μL acetonitrile (ACN) (Optima LC/MS Grade, Fisher Chemical) at room temperature and vortexing for 20 s. A 4:1 ACN/plasma ratio has been reported to achieve a ∼95% protein removal rate .^25^ For the exposomics method only, the ACN contained 0.5% citric acid (CA) (BioUltra, anhydrous, ≥99.5%). After solvent addition, all samples stayed at 4 °C for 20 min, then centrifuged at same temperature, at 20,800*g* for 10 min.

Exposomics supernatants were loaded to HybridSPE-Phospholipid cartridges (500 mg/6 mL, Merck) that had been prewashed with 12 mL MeOH and 12 mL ACN containing 0.5% CA. Samples were eluted with 1 mL ACN containing 0.5% CA, followed by 2 mL MeOH containing 1% ammonium formate (LiChropur, ≥99.0%) into 15 mL propylene tubes (Fisherbrand). The pH of the extracts was adjusted from approximately 3 to 6.5 by adding 40 μL of 25% ammonia solution (LiChropur, LC-MS grade) and centrifuging for 10 min at 4300*g*.

Exposomics and control supernatants were transferred to 5 mL propylene tubes (Eppendorf), evaporated to 100 μL under nitrogen flow and subsequently ultrasonicated for 5 min. A final rinse of the tubes with 100 μL MeOH was performed to reach an extract volume of 200 μL, followed by centrifuge filtration at 10,600*g* for 10 min (0.2 μm nylon centrifuge filters, Thermo Scientific). The final extracts were transferred to amber glass vials (350 μL, Thermo Scientific) and spiked with 10 μL of diuron-*d*_6_ solution (final concentration 4 ng/mL) to correct for extract volume variations and to monitor instrumental performance.

### LC-HRMS Analysis

Extracts were analyzed by ultrahigh pressure LC (Ultimate 3000, Thermo Scientific) with HRMS acquisition (Q Exactive Orbitrap HF-X, Thermo Scientific) in positive and negative electrospray ionization mode (ESI^+^ and ESI^–^) (i.e., two injections per sample). The HRMS was operated with parallel full scan (i.e., MS1; 90–1000 mass-to-charge ratio (*m*/*z*), 120,000 nominal resolution) and data-independent acquisition (DIA) MS/MS (i.e., MS2; 30,000 nominal resolution) with four *m*/*z* precursor windows of equal size (237 Da), with 10 Da window overlap. DIA was used since it is not biased to high-intensity peaks and provides a more comprehensive MS2 acquisition, compared to top-N data-dependent acquisition (DDA) strategies.^26^ DDA was used for analyte confirmation, as it provides higher confidence and cleaner MS2 spectra.^27^ Injection volumes were 10 μL for the control method and 20 μL for exposomics, corresponding to 10 and 20 μL plasma-equivalents on-column, respectively, for a 200 μL sample. The exposomics extracts were also injected at 10 μL to distinguish the injection volume effect from matrix effect removal. Chromatography was at 40 °C on an Acquity BEH C18 column (130 Å, 1.7 μm, 3 × 100 mm, Waters) with an Acquity BEH C18 1.7 μM vanguard pre-column. Upstream of the injector, an Acquity BEH C18 column (130 Å, 1.7 μm, 3 × 30 mm, Waters) was placed to separate instrumental background analytes from sample analytes. A binary gradient elution at 0.4 mL/min used mobile phases (A) water (Optima LC/MS Grade, Thermo Scientific) containing 1 mM ammonium fluoride (Honeywell Fluka, ≥98.0%), previously shown to enhance signals for steroids and xenobiotics^28^ and (B) methanol (Optima LC/MS Grade, Thermo Scientific). The elution gradient started at 5% B, linearly increased to 100% B by 15 min, held until 22 min, and returned to initial conditions with 4 min equilibration.

### Exposomics Method Validation

The optimized exposomics method was validated using pooled Swedish plasma and relevant low concentrations. The method limit of quantification (MLOQ) is thus defined as the lowest spiked concentration in matrix matched calibration curves detected with relative standard deviation (RSD) under 20%. Baseline LC noise was present for some analytes, particularly those eluting in the first half of the chromatogram, and in these specific cases, a signal to noise ratio of 10 was used as an additional requirement for the MLOQ. Briefly, triplicate 200 μL pooled plasma aliquots were spiked before extraction with different volumes of a native and isotopically labelled standard mixture to reach a calibration range of 0.01–100 ng/mL (9 calibration points). When the targeted analyte was present in unspiked plasma, the labelled standard was considered instead. When no labelled standard was available, the instrumental LOQ was reported using a non-extracted solvent-based calibration curve. The matrix-matched calibration curves were also used to evaluate linearity. Carryover was assessed by injecting blank solvent after the highest calibration points.

Extraction recovery was evaluated by comparing samples spiked with the targeted analytes pre-extraction and post-extraction at 5 ng/mL in triplicate, using the formula: % recovery = peak area of pre-spiked plasma/peak area of post-spiked plasma × 100. The RSD of recovery experiments is reported as method precision. Inter-day precision is reported as the % RSD for pooled plasma spiked pre-extraction at 1 ng/mL with labelled standards, over the course of 6 non-consecutive days (3 technical replicates per day). Matrix effects were evaluated by comparing samples to blank solvent, both spiked post-extraction at 0.5 and 5 ng/mL in triplicate, using the formula: % matrix effect = (peak area of post-spiked plasma – peak area of unspiked plasma)/(peak area of post-spiked solvent) × 100. For analytes with high endogenous signals (i.e., peak area of post-spiked plasma/peak area of unspiked plasma < 3), the corresponding isotopically labelled analyte was used for matrix effect calculations.

### Exposomics Application to Individual Plasma Samples

The final exposomics method was applied to 34 individual plasma samples (100 μL) from the Västerbotten Intervention Programme (VIP); a sub-cohort in the Northern Sweden Health and Disease Study.^29^ Sample selection was from among participants in a previous study^30^ whose samples (separate aliquots) had been analyzed for PFAS by a quantitative targeted method,^31^ thereby allowing external validation of the current exposomics method for priority PFAS analytes, namely perfluorooctane sulfonate (PFOS), perfluorohexane sulfonate (PFHxS), perfluorooctanoate (PFOA), perfluorononanoate (PFNA), perfluorodecanoate (PFDA), and perfluoroundecanoate (PFUnDA). The selected samples were collected between 1992 and 2012, from 10 women and 24 men, aged 30–60 years (median 50 years) who had self-reported their smoking/snuff using status. Our targeted and non-targeted study of these samples was approved by the Swedish Ethical Review Authority [Dnr 2020-03301].

### Quantification

Solvent-based external calibration curves with internal standards, were used to quantify most targeted analytes (9 points, 0.005–100 ng/mL). Linear extrapolation was performed to calculate concentrations above the curve range. For quantification of targeted analytes with presence in procedural blanks, only samples with peak areas at least 4 times higher than the blank were considered (see also “Quality Assurance/Quality Control” in Supporting Information). Additionally, reference standardization^32,33^ was used to semi-quantify analytes that were discovered in suspect screening (retrospective quantification), and the steroid hormones due to absence of isotopically labelled steroid standards in the spiking mix.

### Data Processing and Analysis

For analyte quantification, Xcalibur Quan Browser (Thermo Scientific, v.4.1) was used for peak area integration. For the non-targeted comparison of molecular features in exposomics and control workflows, raw data were pre-processed in MS-DIAL (v.4.80) by feature alignment across samples, MS1 and DIA MS2 spectral deconvolution, and peak integration (parameters in Table S4).^34^ Each molecular feature was defined by a chromatographic retention time (RT), an MS1 *m*/*z* and a deconvoluted MS2 spectrum. Spectral matching for annotations with confidence level 2 based on Schymanski et al.^35^ considered an accurate MS1 mass deviation of maximum 0.005 Da between the detected precursor ion and the library record, and a total identification score of >700 (i.e., dot- or reverse dot-product scores >600). Libraries used were MassBankEU (https://massbank.eu/) and Global Natural Product Social Molecular Networking (GNPS; https://gnps.ucsd.edu/). Data for the exposomics and control methods were processed in separate MS-DIAL projects, and the resultant feature lists were combined and analyzed in Python (v.3.7.3)^36^ using Jupyter Notebook (v.5.7.8).^37^ The Python libraries, Plotly (v.5.7.0)^38^ and Seaborn (v.0.9.0)^39^ were used for data visualization and statistical tests were performed with Microsoft Excel.

Downstream data analysis included feature filtering, tagging of diagnostic MS2 ions for phospholipid classification, and estimation of feature overlap between exposomics and control methods. Briefly, for each method, molecular features were considered only if present in all three replicates of Swedish pooled plasma, and eluted after the analytical void volume (i.e., RT > 1.3 min) with signal intensities five times higher than the respective procedural blank. To estimate feature overlap between datasets, an *m*/*z* tolerance of 0.002 Da, and an RT tolerance of 0.9 min were used; the rather high RT tolerance was due to RT shifts between the two methods for eight targeted analytes (eluting between 6 and 14 min) due to differences in pH or ionic strength of the extracts^40^ (e.g., monoethyl phthalate, norharman, and pentachlorophenol had RTs of 6.7, 11, and 14.6 min, respectively, in exposomics versus 6.1, 11.8, and 14.2 min in the control method).

### Classification of Phospholipid Features

Characteristic MS2 fragment ions were used diagnostically to classify non-targeted features as phospholipids (±5 ppm mass tolerance). In ESI^+^, we used the phosphorylcholine fragment ion ([C_5_H_15_O_4_NP]^+^, *m*/*z* 184.0733)^41^ and other head group fragment ions of phosphatidylcholines and sphingomyelins (i.e., [C_2_H_5_NaO_4_P]^+^, *m*/*z* 146.9817 and [C_5_H_13_O_3_NP]^+^, *m*/*z* 166.0627 and [C_2_H_6_O_4_P]^+^, *m*/*z* 124.9998).^42^ Additionally, we used the choline ion which is indicative of 2-lyso phosphatidylcholines (i.e., [C_5_H_14_NO]^+^, *m*/*z* 104.1070),^16^ specific fragment ions of ceramides or ceramide phosphates (i.e., [C_18_H_34_N]^+^, *m*/*z* 264.2686 and [C_18_H_36_N]^+^, *m*/*z* 266.2842)^43^ and sodiated phosphatidylethanolamines ([C_2_H_8_NNaO_4_P]^+^, *m*/*z* 164.0083 and [H_3_NaO_4_P]^−^, *m*/*z* 120.9660).^42^ In ESI-, all glycerophospholipids can fragment to a characteristic head group ion (i.e., [C_3_H_6_O_5_P]^−^, *m*/*z* 152.9958), and other specific fragment ions are markers of phosphatidylethanolamines (i.e., [C_2_H_7_O_4_NP]^−^, *m*/*z* 140.0118, and [C_5_H_11_O_5_NP]^−^, *m*/*z* 196.0380), phosphatidylinositols (i.e., [C_6_H_10_O_8_P]^−^, *m*/*z* 241.0119), as well as phosphatidylcholines and sphingomyelins (i.e., [C_4_H_11_O_4_NP]^−^, *m*/*z* 168.0431 and [C_7_H_17_NO_6_P]^−^, *m*/*z* 242.0798 and [C_7_H_15_NO_5_P]^−^, *m*/*z* 224.0693).^42,44^ For visualization of mass defect regions occupied by phospholipids, relevant masses (*n* = 1915) were retrieved from the LIPID MAPS database.^45,46^

## Results and Discussion

### Multiclass Targeted Exposomics Optimization and Validation

Unlike in the study by Chaker et al.,^10^ in the current work, we did not follow standard manufacturer protocols for the phospholipid removal step with commercial HybridSPE cartridges, as these gave poor recoveries. Instead, the current method was optimized in several ways to achieve clean extracts with the highest quantitative recoveries for the wide range of targeted analytes, as described further in Supporting Information and Figures S1–S8. Preliminary tests on another commercial phospholipid removal column were also conducted, but recoveries were too low or negligible for the perfluoroalkyl analytes (Figure S1) and late eluting non-targeted features (Figure S2). Briefly, a pre-wash step was necessary to remove background PFAS analytes prior to addition of plasma samples. Citric acid was an essential solvent additive for phase conditioning and to quantitatively recover a range of polar analytes that displayed weak affinity for the proprietary zirconia-coated silica particles.^47^ Elution with ammonium formate allowed satisfactory recoveries for organophosphate targeted analytes. Adjustment of eluent pH was beneficial for optimal peak shapes in LC, particularly considering the relatively large 20 μL injection volume. Finally, we avoided evaporating the extracts to dryness to prevent analyte losses, and although this led to compromised peak shapes for some early eluting analytes, this was preferred compared to complete loss of late eluting analytes (Figures S2 and S3). Future method development could focus on peak shape improvement for early eluting analytes.

All method validation results (i.e., MLOQ, recovery, precision, matrix effects, linearity; see Tables S5 and S6) correspond to absolute values that are not corrected by internal standards spiked before extraction; correction improves all parameters. The median extraction recovery of 77 targeted analytes at 5 ng/mL was 82% (range 61–104%) and median method precision was 7% RSD (range 0.2–18%). Median inter-day precision was 11% (range 7–35%, <25% for 94% of analytes). Matrix effects at 0.5 and 5 ng/mL were generally low for the phospholipid-free matrix, with medians of 91 and 107%, respectively. Overall method sensitivity was excellent to satisfactory for most targeted analytes, with median MLOQ being 0.05 ng/mL, and 62% of analytes having MLOQ between 0.01 and 0.1 ng/mL, 34% between 0.2 and 1 ng/mL, and only 4% between 1 and 5 ng/mL. Linearity in matrix matched calibration curves between MLOQ and 100 ng/mL (i.e., analytes spiked pre-extraction), was excellent over up to 4 orders of magnitude (*R*^2^ ≥ 0.97, median 0.996), and in these concentration ranges, no targeted analyte carryover was detectable in the instrument. For clarity, we note that all of the above validation results are based on combined targeted and non-targeted acquisition (i.e., full scan MS1 and parallel DIA MS2) and theoretically can be improved in a pure targeted acquisition.

### Targeted Analyte Performance of Exposomics

**Figure 1:**
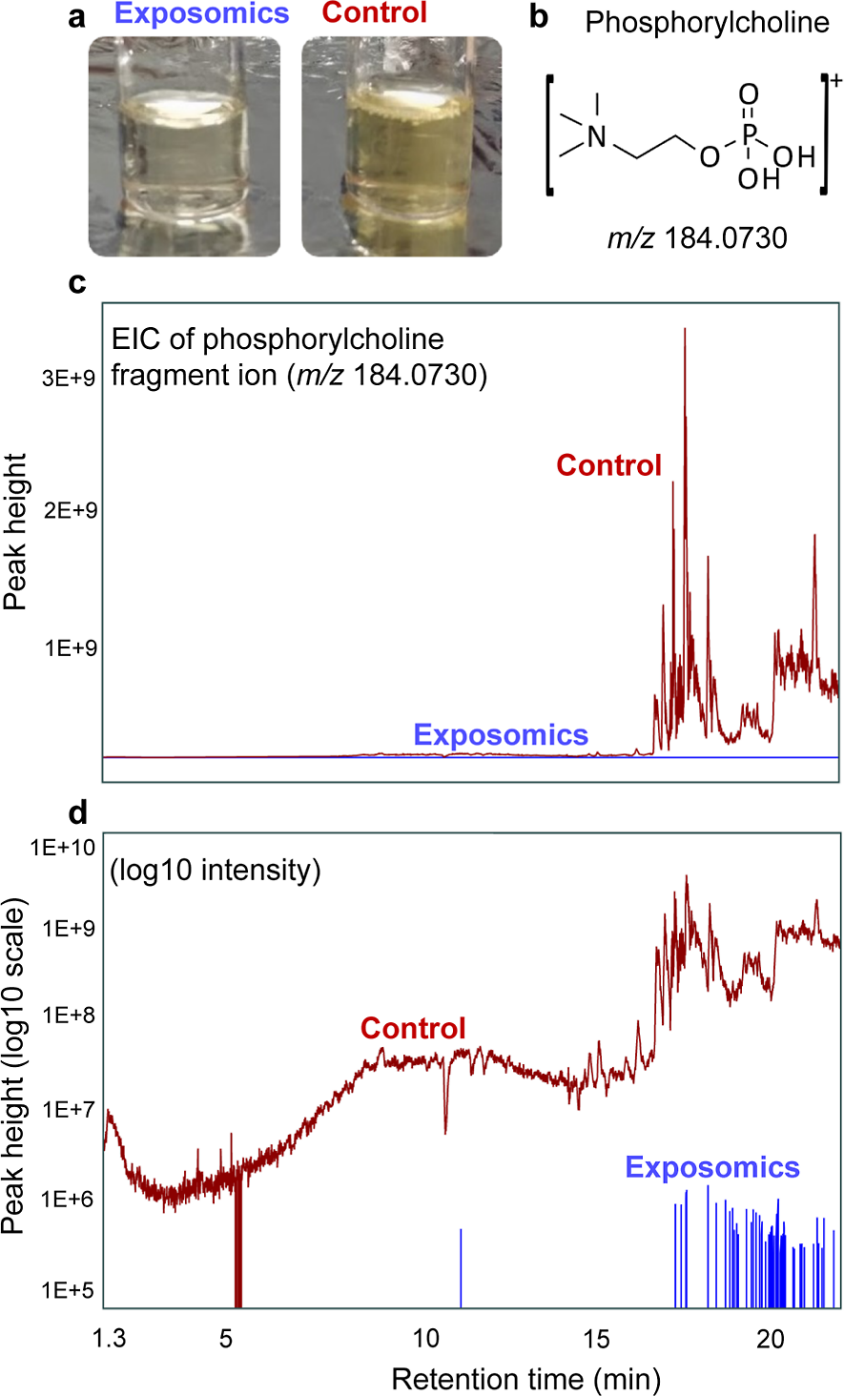
Comparison of background phospholipid signal in plasma extracts from exposomics and control protocols. (a) Photos of representative plasma extracts from the exposomics protocol (left) and control protocol (right); each sample was concentrated 4× to better visualize the differences in color. (b) Structure and exact mass of phosphorylcholine ([C_5_H_15_O_4_NP]^+^, *m*/*z* 184.0730) which is used as a characteristic MS2 fragment ion marker of plasma phospholipids in ESI^+^ mode. (c) MS2 EIC of phosphorylcholine in ESI^+^ mode for representative plasma extracts from exposomics (blue) and control (red) protocols. (d) Identical to panel (c) but with log_10_ transformed *y*-axis intensities.

The exposomics protocol resulted in visually clearer extracts than the control protocol (Figure 1a). Although not the focus here, the exposomics extracts remained clear even after four-fold concentration by nitrogen evaporation, whereas the control extracts precipitated and became cloudy with the same treatment (Figure S9). To confirm that this difference could be attributable to phospholipid removal in the exposomics protocol, we examined the extracted ion chromatogram (EIC) of the phosphorylcholine fragment in ESI^+^ MS2 (Figure 1b–d). In the exposomics extracts, this diagnostic fragment was effectively absent (Figure 1c,d), demonstrating effective phospholipid retention on the cartridges despite the additional washing procedures included here for improved analyte recovery. In contrast, the log_10_ version of the phosphorylcholine EIC (Figure 1d) revealed that the phosphorylcholine fragment was present in the control method throughout the entire chromatographic range (RT 1.3–22 min, and particularly abundant after 15 min), highlighting that the complex mixture of endogenous phospholipids could interfere with all other analytes, irrespective of RT. By effectively removing this background with the exposomics protocol it was, therefore, of interest to understand the associated impact on response of targeted analytes and non-targeted molecular features.

In unspiked pooled Swedish plasma, 24 targeted analytes were detected by both protocols, with a median 2-fold peak area enhancement by exposomics compared to the control method (Figure S10a). The peak areas of the 34 internal standards spiked before extraction also showed a median 2-fold enhancement by exposomics (Figure S10b). These results were partly anticipated due to the double injection volume of the exposomics protocol, but confirmed that phospholipid removal did not compromise the recoveries of targeted analytes and that there was negligible matrix effect, consistent with validation tests described above. Two notable exceptions were the earliest eluting analytes, namely acesulfame (2.5 min RT) which was suppressed in exposomics and acephate-acetyl-*d*_3_ (4.5 min RT) which was neither enhanced nor suppressed in exposomics (Figure S10). This was likely related to the higher injection volumes used in the exposomics protocol, which can decrease retention of the earliest eluting analytes due to higher organic solvent volume injected to the column.^48^ When exposomics samples were injected at 10 μL, median fold change was 0.9 for the targeted analytes and 0.94 for the internal standards (Figure S11).

A notable benefit of the exposomics protocol was evident for the targeted analyte, perfluorooctane sulfonamide (FOSA; C_8_F_17_SO_2_NH_2_), which was not detectable from noise by the control protocol but was detected by exposomics as an abundant mixture of linear and branched isomers, that is, a hallmark of its electrochemical fluorination manufacturing sources^49^ (Figure S12). Similarly, FOSA was abundantly detected by exposomics with a 10 μL injection (Figure S11), thus removal of phospholipids can explain the improved sensitivity by exposomics. Consistent with this, FOSA was the latest eluting targeted analyte (RT = 16 min) among analytes detected in the pooled plasma and within the retention range where the phosphorylcholine fragment ion intensity increases in the control protocol (Figure 1d).

### Non-targeted Feature Acquisition by Exposomics

**Figure 2:**
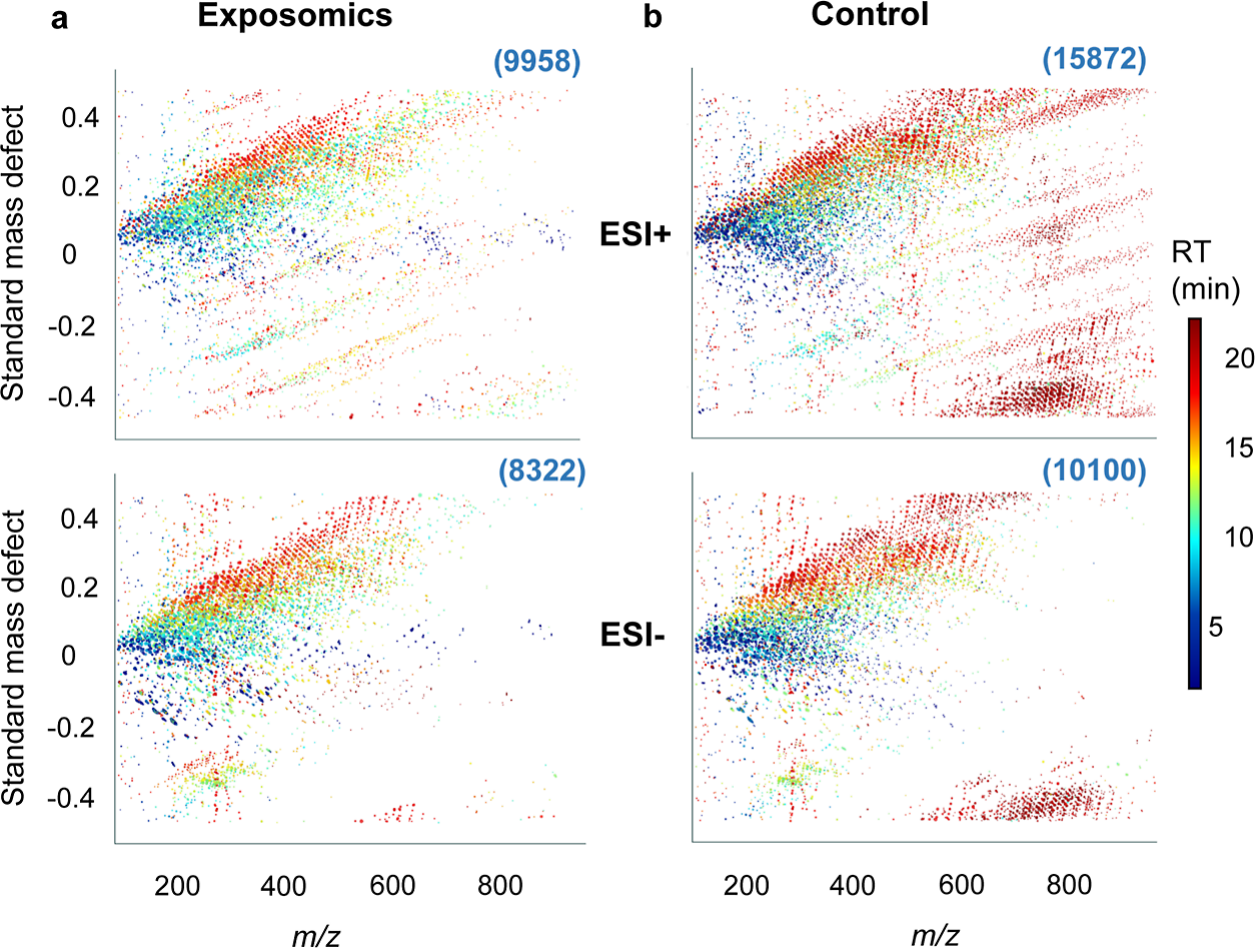
Non-targeted LC-HRMS feature comparison between exposomics and control protocols. Standard mass defect plots of all non-targeted features in ESI^+^ and ESI^–^ detected in pooled plasma extracts by (a) exposomics and (b) control protocols. Features are colored by RT (1.3–22 min), with size of each feature marker scaled by relative peak area. Numbers in brackets, in blue font, are total features by each method in each mode.

The detected targeted analytes represent only a very small fraction of small molecules in human plasma. Thus, mass spectral acquisition by full scan MS1 in combination with DIA MS2 allowed for a deeper comparison by considering all non-targeted features. Results were broadly visualized in standard mass defect plots, colored by RT (Figure 2). As expected, due to the intended removal of phospholipids, fewer features were detected by exposomics compared to the control method (i.e., 37% fewer features in ESI^+^, 18% fewer in ESI^–^). The greater decrease of feature counts in ESI^+^ can be explained by the preference of the most abundant plasma phospholipids (i.e., phosphatidylcholines) to ionize in ESI^+^.^41,50^ The loss of features by exposomics was mostly in the mass defect plot regions where phospholipids should appear, as shown by plotting relevant masses from the LIPID MAPS database^45,46^ (Figure S13), and suggesting strong selectivity of the phospholipid removal step.

Considering all diagnostic phospholipid fragment ions (see Experimental Section), 72% of all features in the control protocol in ESI^+^ (11,413 of 15,872 features) and 39% in ESI^–^ (3932 of 10,100 features) were classified as phospholipids (Table S7 and Figure S14). For the exposomics extracts, only 6% of all features in ESI^+^ (628 of 9958 features) and 5% in ESI^–^ (452 of 8322 features) could be classified as phospholipids by identical criteria. At the same time, the exposomics protocol resulted in increased detectable non-targeted features that could not be classified as phospholipids. This indicated complementary molecular coverage by the exposomics protocol, potentially including low abundance metabolites or environmental substances. For discussion purposes, we refer to these here as new features. More specifically, the exposomics method showed a 109% increase in non-phospholipid features in ESI^+^ (7931 new features) and a 28% increase in ESI^–^ (5303 new features) (Table S7). New features detected by exposomics are unlikely due to increased adduct formation, in fact a higher percentage of features were labelled as adducts in the control method (32% in ESI^+^, 20% in ESI^–^) than by the exposomics protocol (27% in ESI^+^, 17% in ESI^–^).

**Figure 3:**
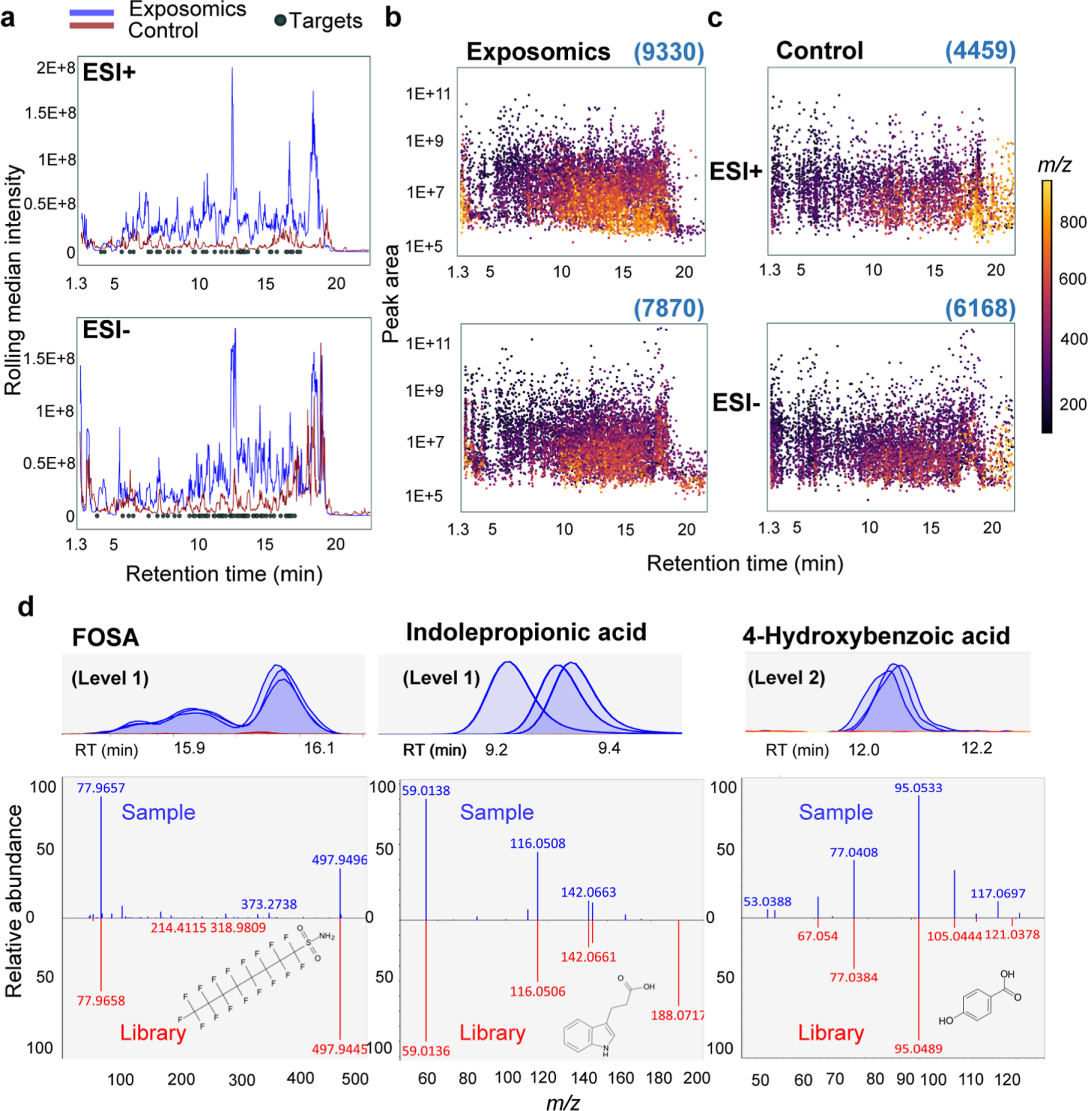
Non-phospholipid features detected by the exposomics and control protocols in triplicate pooled Swedish plasma. Panel (a) shows the rolling median intensity of non-phospholipid features across the chromatographic range in the exposomics (blue) and control (red) plasma extracts in ESI^+^ and ESI^–^. RT of the targeted analytes are shown as dark grey dots parallel to the *x*-axis. Panels (b,c) display the non-phospholipid features detected across the chromatographic range (*x*-axis, 1.3–22 min) along with their peak areas (*y*-axis, log_10_ transformed) and *m*/*z* (color scale, 90–1000 *m*/*z*) for the exposomics and control protocols, respectively. Numbers in brackets, in blue font, are total non-phospholipid feature counts by each protocol in two ESI modes. Panel (d) displays chromatographic peaks for three example substances detected only in the exposomics extracts, identified at confidence level 1 or annotated at level 2 by deconvoluted DIA MS2 spectra matching to the GNPS/MassBankEU database.

Moreover, the rolling median intensity of all non-phospholipid features (common features, and those specific to each protocol) was consistently higher for the exposomics protocol across the 4–19 min chromatographic range in ESI^+^ (6-fold on average, 28-fold max) and ESI^–^ (4-fold on average, 58-fold max) (Figure 3a). Regarding common non-phospholipid features between the two methods, in ESI^+^ 76% of these had enhanced signals by exposomics (2-fold median, approx. 900-fold max) and in ESI^–^ 86% had enhanced signals by exposomics (2-fold median, approx. 500-fold max) (Figure S15). These non-targeted results together indicate enhanced performance by exposomics beyond the two-fold increased injection volume and are consistent with observations for the targeted analyte FOSA as described above (Figure 3d). The results obtained by injecting exposomics extracts at 10 μL further support that observed enhancement was a result of matrix effect reduction after phospholipid removal, as well as the increased injection volume that was thereby enabled. More specifically, the number of non-phospholipid features was still higher in exposomics extracts injected at 10 μL (5646 in ESI^+^, 6593 in ESI^–^) compared to the control extracts (4459 in ESI^+^, 6168 in ESI^–^). Additionally, the rolling median intensity of non-phospholipid features was enhanced in the chromatographic range of 4–19 min compared to the control protocol (2-fold on average, 11-fold max in ESI^+^ and 1.5-fold on average, 29-fold max in ESI^–^).

In total, 220 non-phospholipid features were annotated at confidence level 2 with the exposomics method (76 in ESI^–^ and 144 in ESI^+^) (Table S8). Out of these, 32 were new features (i.e., not present in the control method) and examples include 4-hydroxybenzoic acid, which has wide applications in the cosmetic, pharmaceutical and food industry,^51^ and the microbial metabolite indolepropionic acid, which has been associated with lower risk for type 2 diabetes^52^ (Figure 3d). Indolepropionic acid was subsequently confirmed at level 1 by DDA (Figure S16). Importantly, the new features in Figure 3d were also abundant in exposomics injected at 10 μL (Figure S17). The level 2 features detected by both methods (187 features) showed an average 3-fold and a maximum 87-fold increased response by exposomics. This is a bit lower but generally consistent with the average increase of all non-phospholipid features by 4- to 6-fold in ESI^–^ and ESI^+^, respectively, based on rolling median intensity (Figure 3a).

The increased non-phospholipid feature detections by exposomics, and the associated signal enhancement can be explained by the combination of phospholipid removal, ion suppression reduction,^16^ and increased injection volume. Similarly, Tulipani et al. observed enhancement of the nonlipid metabolite coverage after phospholipid removal in plasma metabolomics, although they focused only on targeted or annotated metabolites and did not investigate larger injection volumes.^20^

### Limitations to Chemical Space Coverage by the Exposomics Protocol

Despite the excellent performance of the method for targeted analytes and enhanced discovery of non-phospholipid features, only 31% of non-phospholipid features detectable by the control method in ESI^+^ and 42% in ESI^–^ were detected by exposomics. We acknowledge that feature alignment could not be performed in an ideal way due to the observed RT shifts. However, these lost features are mainly the latest eluting after 19 min (Figure 3a–c), an extremely hydrophobic chromatographic region that is well beyond the elution range of all 77 targeted substances, and the range of observed RT shifts. The latest eluting targeted analytes were octocrylene (C_24_H_27_NO_2_, 17.4 min) and perfluorotetradecanoate (C_14_F_27_O_2_^–^, 16.6 min) in ESI^+^ and ESI^–^, respectively. We confirmed that these lost non-targeted features could not be classified as phospholipids with the same fragment criteria noted above; some were tentatively free sterols or sterol esters, as observed by monitoring the sterol specific MS2 fragment ([C_27_H_45_]^+^, *m*/*z* 369.3516).^53^ Any of these lost hydrophobic features may be of limited relevance to environmental exposure and more suitably analyzed by metabolomic and lipidomic protocols or by complementary GC-methods,^4^ such as that of Hu et al.^7^

Similarly, there was loss of some of the earliest-eluting features by exposomics (i.e., RT < 4 min, Figure 3a–c). This was likely related to the higher injection volumes used in the exposomics protocol, which led to poorer chromatographic retention for some of these earliest eluting polar compounds, as discussed above for acesulfame and acephate-acetyl-*d*_3_ (non-targeted examples in Figure S18). In contrast with the late eluting hydrophobic features, this early chromatographic region contains very hydrophilic substances which are most likely better monitored in urine samples and with alternative chromatographic modes (e.g., HILIC).^12^ Acesulfame for instance is typically analyzed in urine where it is exclusively excreted in humans.^54,55^ In agreement with these observations, the majority of level 2 non-phospholipid features that were present only in the control protocol eluted either before 4 min or after 18 min (20 out of 35 features, Table S7). Nevertheless, the targeted analytes in this study, which were selected from among diverse known environmental contaminant classes in human serum or urine, and with log *P* ranging between −3.6 and 7.2, generally eluted in the chromatographic range where enhancement by exposomics was evident.

### Application of Exposomics to Individual Plasma Samples

**Figure 4:**
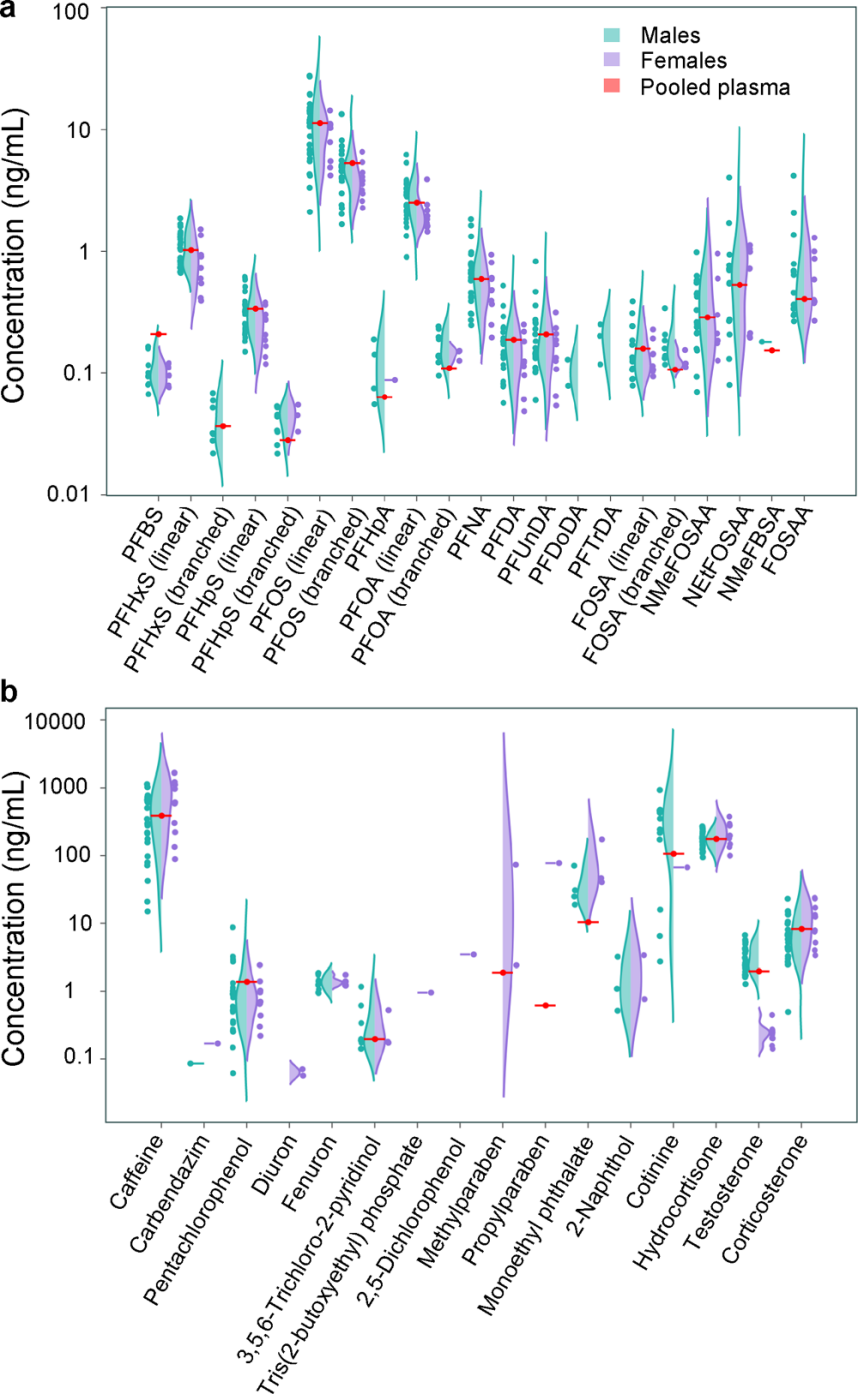
Quantification of targeted and suspect analytes in individual adult plasma (violin plots) and in pooled reference plasma (red lines). Violin plots of analyte concentrations (log_10_ scale) are shown for individual males (green, *n* = 24) and females (purple, *n* = 10). Panel (a) shows per- and polyfluoroalkyl substances and panel (b) shows all other detected analytes, including endogenous steroid hormones and a range of environmental exposures from diet, pesticides, personal care products, and commercial or industrial chemicals.

The exposomics protocol was applied to 34 adult plasma samples from the Swedish VIP cohort, separate aliquots of which had previously been analyzed by a targeted PFAS method,^30^ thereby allowing external method validation for six major PFAS. Only 100 μL of plasma was available from samples in this cohort but the method was applied with no adjustments; experiments with 100 μL of plasma showed comparable extraction recovery and precision to the optimized protocol which used 200 μL (Table S9), however, MLOQs are approximately 2-fold higher (adjusted MLOQs for detected targeted analytes in Table S10). In total, 26 targeted analytes and 2 additional compounds (fenuron and carbendazim, added after preliminary method validation), were detected and quantified, representing a diverse range of substances from 10 chemical classes and reflecting chemical mixture exposures between 1992 and 2012 when the samples were collected (Table S10, Figures 4 and S19–S46).

#### Per- and Polyfluoroalkyl Substances

The detected substances included 12 linear PFAS, 5 of which had respective branched isomers detectable at earlier RTs, thereby demonstrating good chromatography and high method sensitivity for these small plasma volumes. One example was the detection of branched PFOA isomers, which indicates exposure to 3M’s historic electrochemical PFOA that was phased-out of manufacturing by 2002, substituted by linear PFOA from telomer-manufacturing sources.^49^ The branched PFAS isomers were quantified separately for each analyte as “total branched isomers”, thereby raising the total number of quantified analytes to 33. Mean concentrations for many PFAS, including the respective branched isomers, were lower in women than men (Figure 4a: PFHxS, perfluoroheptane sulfonate (PFHpS), PFOS, perfluoroheptanoate (PFHpA), PFOA, PFNA, PFDA, and PFUnDA). In fact, the least frequently detected perfluorododecanoate (PFDoDA), perfluorotridecanoate (PFTrDA), and total branched PFHxS were only detectable in men. Previous findings report that breastfeeding, menstruation, and pregnancy are important elimination routes for several PFAS, leading to lower levels in women.^56−58^

Compared to previous targeted analysis of 6 PFAS in the same samples, published in 2019,^30^ total PFOS (i.e., sum of linear and total branched isomers), total PFHxS, total PFOA, and linear PFNA were statistically linearly associated (*p* < 0.01), with slopes in the range of 0.73–1.05, and high Pearson correlation coefficients (0.92–0.97) (Figure S47). Bland–Altman plots (Figures S48 and S49) showed absolute bias of 1.04 ng/mL for PFOS, 0.40 ng/mL for PFOA, 0.07 ng/mL for PFHxS and 0.08 ng/mL for PFNA, which corresponds to relative bias of 9.5% for PFOS, 10.9% for PFOA, 2.0% for PFHxS and 0.6% for PFNA. These comparisons further support that the current multiclass targeted approach is quantitative for these major PFAS. PFDA and PFUnDA could not be compared between studies because their detection frequency in the previous study was below LOQ in more than 70% of the samples, but detected consistently here which further demonstrates the sensitivity for the exposomics method.

#### Cotinine

Cotinine, the major plasma metabolite of nicotine, was detected in 38% of samples and concentrations were consistent with the reported smoking status of the individuals (see biphasic distribution for cotinine in Figure 4b). Smokers and/or snuff users had concentrations in the range of 67.0–933 ng/mL, in agreement with previously reported values for smokers (250–300 ng/mL average, 900 ng/mL max).^59^ The remaining cotinine detections corresponded to “former” or “never smokers/snuff users” and ranged between 2.7 and 15.9 ng/mL, consistent with reference values for passive exposure.^60^

#### Personal Care Products

Chemicals associated with personal care products or their metabolites (methylparaben, propylparaben, and 2,5-dichlorophenol) were only detectable in women (Figure 4b), consistent with previous reports of higher urinary paraben concentrations in women compared to men.^61^ Similarly, the plasticizer metabolite monoethyl phthalate, which is also used in personal care products,^62^ was detected in women at higher concentrations than in men (Figure 4b).

#### Pesticides and Insecticides

The fungicide pentachlorophenol was detected in all individuals (median 0.7 ng/mL, max 8.7 ng/mL) at concentrations comparable to previously reported levels in serum of pregnant women in Sweden (0.6–9.5 ng/g, years 1996–1999).^63^ The metabolite of the insecticide chlorpyrifos, 3,5,6-trichloro-2-pyridinol (TCPy), was detected in 32% of individuals (median 0.2 ng/mL, max 1.2 ng/mL). Interestingly, chlorpyrifos has never been approved for plant protection in Sweden, but TCPy detection can be linked to consumption of imported foods containing chlorpyrifos.^64^ TCPy was previously detected at high detection frequencies (≥99%) in urine of Swedish adolescents (median concentrations 0.82–1.41 μg/L through years 2000–2017).^64^ In January 2020, chlorpyrifos was banned in Europe and maximum residue levels are monitored in European food,^65^ thus chlorpyrifos metabolites will likely be lower today than in these archived samples.

Trace amounts of the herbicide diuron and the fungicide carbendazim were detected infrequently, but it was notable that the phenylurea herbicide fenuron was detected at relatively high frequency (56% of individuals, median 1.4 ng/mL, max 1.8 ng/mL). Fenuron has not previously been reported in human biofluids to our knowledge but was reported in hair samples of mothers living in France in 2011 (44% detection frequency, 0.09 pg/mg at the 75th percentile).^66^ Fenuron was never approved as a pesticide in Sweden^67^ and was banned in Europe in 2002.^68^ However, it is still commonly used in the building and construction industry as an additive in sealants, adhesives, fillers, polymers, and is still detectable in European rivers.^69^

#### Steroids and Miscellaneous Analytes

Testosterone, corticosterone and hydrocortisone were quantified in all individuals. Estradiol could not be detected as the MLOQ (0.5 ng/mL) was too high to detect known human plasma levels (i.e., <0.01–0.35 ng/mL).^70^ Testosterone concentrations, which were calculated with the semi-quantitative method of reference standardization, clearly delineated samples of men (1.3–6.7 ng/mL) and women (0.1–0.4 ng/mL), and all in accordance with reference values for healthy adults in comparable age groups (Figure 4b).^71^ This result further supports the proposal of Go et al. regarding the applicability of reference standardization to exposomics studies where solvent based calibration curves with internal standards are too costly and too tedious to effectively implement, or when non-targeted analytes are detected, allowing retrospective quantification without sample reinjection.^32^

Caffeine was measured in all individuals and at the highest concentrations among all targeted analytes (median 326 ng/mL, max 1661 ng/mL). The chemical 2-naphthol, a naphthalene metabolite, was quantified in 5 individuals (0.5–3.4 ng/mL) and the flame-retardant tris(2-butoxyethyl) phosphate in one individual (1.0 ng/mL).

### Suspect Screening for PFAS Precursors

To further explore the non-targeted data, we performed suspect screening of PFAS precursors that were included in a previous study of Swedish foods.^72^ Perfluorooctane sulfonamidoacetic acid (FOSAA), as well as its *N*-methyl and *N*-ethyl derivatives (NMeFOSAA and NEtFOSAA), and *N*-methyl perfluorobutane sulfonamide (NMeFBSA) were annotated in the current samples based on accurate mass and presence of characteristic MS2 fragments and were subsequently confirmed with authentic standards (level 1 confidence, Figures S50–S53).^35^ By applying the method of reference standardization, these substances were retrospectively semi-quantified (Figure 4a and Table S10) by point calibration to the pooled Swedish plasma. Like FOSA (a targeted PFAS precursor), FOSAA, and NEtFOSAA were more frequently detected in individuals sampled before the year 2000 (*n* = 17), compared to those sampled after 2000 (*n* = 17), and with higher mean concentrations (*p* < 0.05; Student’s *t*-test, two-tailed) (Figure S54), confirming previous findings that the PFAS exposome has been changing with time. NMeFOSAA did not have a significant decreasing trend over time in these few selected samples, but declining concentrations of FOSA, FOSAA, NMeFOSAA, and NEtFOSAA have been shown in Swedish serum previously, between 1996 and 2012.^73,74^

Perfluorobutane sulfonate (PFBS) was a frequently detected targeted analyte, and by suspect screening a PFBS-precursor, NMeFBSA, was also detected and quantified (level 1 confidence, Figure S52), but only in one individual sample (from 2002) and in the pooled Swedish plasma. NMeFBSA is believed to be an indicator of exposure to contemporary 3 M PFAS formulations that replaced PFOS-based formulation by 2002,^75^ but it has only rarely been reported in human samples; one blood sample from Örebro, Sweden (2018).^76^

### Applicability and Future Directions of the Chemical Exposomics Protocol

The chemical exposomics method described here is not meant to replace metabolomic analyses in human studies, but brings clear advantages of method sensitivity that allows trace analytes in the exposome to be more easily detected, quantified, or discovered. In fact, the current data demonstrate that exposomic and metabolomic approaches (as represented by our control method) are highly complementary, and thousands of additional molecular features can be followed if both approaches are applied together for wide coverage of environmental and endogenous substances. Indeed, specific plasma phospholipids have been associated with environmental chemical exposures,^77,78^ but it is evident here that removal of phospholipids brings advantages to exploration of the exposome, consistent with previous conclusions for exploration of the metabolome.^20^ Application of both methods to the same samples may be limited by sample volumes, as well as cost and analysis time considerations. Applications of the current exposomics method will depend on case-specific hypotheses and resources, and increased efficiencies might be gained in future by introducing polarity switching, as others have shown for combined exposomics and metabolomics.^12^

Compared with the control method, the current exposomics method requires additional steps, cost, and time, but it is only notable that the commercial cartridges used here are already offered in 96 well format.^47^ Thus, the current technique is scalable and can feasibly be adapted to liquid handling robotics with some modifications. Moreover, the cleaner matrix injected by exposomics may have advantages for throughput, whereby instrumental performance should be less impacted by ionization sensitivity drift and gradually increasing background signals.^79^ Finally, removal of major matrix ions from MS2 acquisition across the entire chromatographic range (Figure 1d) may also contribute to more accurate (i.e., cleaner) MS2 spectra in DIA acquisition mode (which requires a software deconvolution step to remove non-specific fragment ions), thereby perhaps assisting with MS2 spectral library matching and *in silico* molecular annotation.

## Supporting Information Available

Supporting figures and detailed description of method development, links to publicly available python scripts and identification numbers for MS data from pooled samples publicly deposited to the MassIVE database (PDF)
(XLSX)

## Notes

Access to data for individual plasma samples can be provided for research purposes but may be limited by laws regarding the privacy of research participants. Requests should be sent to the Biobank Research Unit at Umeå University (contact: ebf@umu.se).
