# Supplementary material for: Phospholipid Removal for Enhanced Chemical Exposomics in Human Plasma
Source: Environ Sci Technol. 2023 Jul 3;57(28):10173–84. doi: 10.1021/acs.est.3c00663 (PMC10357568; doi:10.1021/acs.est.3c00663)
Supplement: Supplementary file 1 — es3c00663_si_001.pdf [file es3c00663_si_001.pdf]

Supporting Information to:

Phospholipid Removal for Enhanced Chemical Exposomics in Human Plasma

Kalliroi Sdougkou[1], Hongyu Xie[1], Stefano Papazian[1,2], Bénilde Bonnefille[1], Ingvar A. Bergdahl[3], Jonathan W. Martin[1,2]*

[1]Department of Environmental Science, Science for Life Laboratory, Stockholm University, Stockholm 106 91, Sweden

[2]National Facility for Exposomics, Science for Life Laboratory, Stockholm University, Solna 171 65, Sweden

[3]Department of Public Health and Clinical Medicine, Section for Sustainable Health, Umeå University, Umeå 901 87, Sweden

Corresponding author: Jonathan W. Martin; Email: jon.martin@aces.su.se

This file contains:

Supplementary Methods p. S2-S5

Supplementary Figures p. S6-S59

Supplementary Tables

(separate file; XSLX)

Supplementary Dataset

MS datasets of pooled samples have been deposited as mzXML files at the MassIVE database with the identifications: GNPS Swedish Human Pooled Plasma LC-Orbitrap ESI+ (MSV000091086), GNPS Swedish Human Pooled Plasma LC-Orbitrap ESI- (MSV000091087).

Supplementary python scripts

Python scripts are available at the following links: Feature filtering and tagging MS2 ions script (10.6084/m9.figshare.21930945), Common features script (10.6084/m9.figshare.21930936).

Supplementary Methods

Method Development and Optimization

Initially the HybridSPE-Phospholipid cartridges (500 mg/6 mL, Merck) were tested against the Captiva EMR-Lipid cartridges (600 mg/6 mL, Agilent). The HybridSPE-Phospholipid sorbent consists of a Zirconia (Zr) coated silica-based material and targets the retention of phospholipids by Lewis acid-base interactions.[1] The EMR lipid sorbent retains lipids by size exclusion and hydrophobic interaction.[2] The recovery of isotopically-labelled per- and polyfluoroalkyl substances (PFAS, specifically M8FOSA and MPFAC-MXA, Wellington Laboratories) was tested with the two types of cartridges. Experiments were conducted in triplicate at a spiking level of 5 ng/mL and the applied conditions were based on manufacturer recommendations. Briefly, serum aliquots (500 µL, pooled human serum Merck) were protein precipitated by addition of 1.5 mL acetonitrile (ACN) and centrifugation (20,800 x g for 10 min at 4 °C). The supernatants were added to the cartridges (previously preconditioned with 3 mL 80% ACN in water for EMR and 3 mL ACN for HybridSPE), and elution was with 1 mL 80% ACN in water for EMR and 1 mL ACN for HybridSPE. All extracts were then evaporated to 100 µL under nitrogen flow and diluted with methanol (MeOH) to a 75% MeOH composition. Median recovery was 81% by HybridSPE, versus 68% for EMR, which additionally showed negligible recovery for labelled FOSA (**Figure S1**).

The effect of evaporation to dryness was studied for the exposomics extracts. Serum aliquots were extracted with HybridSPE as described above, and were either evaporated to dryness and reconstituted to 250 µL with 25% MeOH, or evaporated to 100 µL and diluted to 250 µL to reach a final composition of 25% MeOH (n = 3 for each condition). Fewer late eluting features (RT > 15 min, red color in mass defect plot) were detected with Hybrid SPE when samples were evaporated to dryness (**Figure S2a, S2b**). Moreover, samples that had been dried had lower recovery of labelled PFAS standards (spiked before sample preparation at 2 ng/mL, **Figure S3**), and the longer-chain length labelled PFAS (PFDA, PFUnDA and PFDoDA) were non-detectable. As a result, evaporation to dryness was avoided in further method optimization. Notably, when the protocol with no evaporation to dryness was applied to EMR cartridges, considerably less late eluting features (RT > 15 min) were detected compared to the HybridSPE cartridges (**Figure S2c**). Based on these results, only HybridSPE was chosen for further method development and optimization.

Solvents used in sample preparation were then optimized to ensure minimal analyte retention on the HybridSPE sorbent. Using the manufacturer recommendations as a starting point, acetonitrile with no additives or acetonitrile with either 1% formic acid (FA) or 0.5% citric acid (CA) were tested as a pre-wash (conditioning), protein precipitation and elution solvents (n = 3 for each condition, 5 ng/mL

spiking). Average recoveries for 57 targeted analytes were 72% with no additive, 61% with FA and 69% with CA (**Figure S4a**). Importantly, not using additives, despite resulting in highest average recoveries, led to complete loss or very poor recovery (< 10%) of several analytes, namely 2,4-dichlorophenoxyacetic acid, 2,5-dichlorophenol, 3,5,6-trichloro-pyridinol, mono-(2-ethyl-5-hydroxyhexyl) phthalate and pentachlorophenol (**Figure S4a**). These analytes were recovered to some degree by using acidic additives. This can be explained by the formate and citrate ions being strong Lewis bases and competing with acidic analytes on the Zr sites, preventing their retention.[3] Additionally, residual silanols are deactivated by acidic conditions created by the additives, preventing the retention of basic analytes.[3] However, a few analytes were not recovered even with acidic conditions, namely diphenyl phosphate, bis(1,3-dichloro-2-propyl) phosphate and 4-methylhippuric acid.

Ammonium formate in MeOH (1%) was tested as a second elution solvent (n = 3, 5 ng/mL spiking) (**Figure S4b**). Recoveries were enhanced with the addition of ammonium formate for both acidic additives. The combination of FA and ammonium formate gave average recoveries of 79% while the citric acid combination resulted in 85% average recoveries (Figure S3b). Additionally, the citric acid combination allowed the sufficient recovery of equol and 4-methylhippuric acid, which were either strongly retained or poorly recovered with the formic acid combination. Doubling the volume of the MeOH solvent with ammonium formate allowed also the recovery of the organophosphate analytes, diphenyl phosphate and bis(1,3-dichloro-2-propyl) phosphate (**Figure S5**), without resulting in an unwanted elution of phospholipids. Loss or poor recoveries of a few late eluting analytes was however noted, which was examined in later experiments, as explained below (triclocarban, tetrabromobisphenol A, PFTeDA, see **Figure S5**).

The acidic elution solvents used in the protocol led to a low pH (pH ~ 3), thus addition of 40 µL ammonia solution (25%) was utilized to raise the pH of extracts to approximately pH 6.5. The benefit of this adjustment was that basic analytes would not be fully ionized (i.e. protonated) and were therefore better retained on the reversed phase liquid chromatography (LC) column. This was particularly important with the larger injection volumes used in the exposomics protocol here (i.e. 20 µL), and some analytes that showed peak shape improvement after pH adjustment were paracetamol, cotinine and NAAL (see **Figure S6** for example of paracetamol).

The preconditioning step was also adjusted to eliminate background PFAS contamination in the HybridSPE cartridges, an issue which has been previously reported.[4] A combination of MeOH and ACN with 0.5% CA for preconditioning (12 mL of each solvent) was the most efficient in reducing background PFAS levels and was further used.

Finally, a tube washing step after extract evaporation to 100 µL was added to consistently improve recoveries of relatively hydrophobic analytes (such as pentachlorophenol and longer chain PFAS), which showed poorly reproducible recoveries due to adherence on the tube walls. Three solvent combinations were tested, namely 100% MeOH, 75% MeOH in water, and 50% MeOH in water (100 µL volume, n = 3 for each condition). The strongest solvent of 100% MeOH resulted in the highest recoveries of spiked analytes (5 ng/mL) and was therefore included in the final method (**Figure S7**).

For LC-MS, the buffer for the aqueous mobile phase (A) was chosen based on a sensitivity test comparing 4 conditions (**Figure S8**). Ammonium fluoride (1 mM) provided the most intense peaks compared to 3 other modifiers tested (2 mM ammonium acetate, 2 mM ammonium formate, 0.1% formic acid) for the majority of targeted analytes, with standard solution injections at 5 ng/mL. A previous study also reported signal enhancement of steroids and xenobiotics with the addition of ammonium fluoride to the aqueous mobile phase.[5]

LC columns with a relatively high inner diameter of 3 mm were employed to better accommodate injection volumes of 20 µL.[6] A flow rate of 0.4 mL/min was chosen since it resulted in good separation between close eluting isomers, such as 1- and 2- naphthol and linear and branched PFAS isomers, while not exceeding column pressure limits.

Quality Assurance / Quality Control

All samples were prepared in a positive pressure clean laboratory with high efficiency particulate filtration. Procedural blanks were prepared for all experiments (validation, comparison of exposomics and control protocols, analysis of individual samples) and treated in the same manner as the experimental plasma samples. Targeted analytes were in general absent in procedural blanks, however peaks were detectable for the plasticiser chemicals and metabolites (bisphenol A, monoethyl phthalate, monoisobutyl phthalate), personal care product chemicals (methylparaben, propylparaben, and oxybenzone), flame retardants or metabolites of them (diphenyl phosphate, tris-2-butoxyethyl phosphate) and the insecticide DEET. For quantification of the above targeted analytes only samples with a peak area of at least 4 times higher than the blank signal were considered. The blank concentration was then subtracted from the sample concentration. Instrumental blanks consisting of clean solvent were also run multiple times in the injection sequence to prevent carryover.

All 34 cohort samples were extracted within the same day along with 3 procedural blanks and 3 Swedish pooled plasma aliquots (used for reference standardisation). Then they were stored at 4 °C

overnight and analysed the next morning. Analysis of cohort samples, calibration solutions, blanks (procedural and instrumental) and Swedish pooled plasma samples lasted approximately 70 hours (26 minutes per injection in a single ESI mode), during which the samples stayed at 10 °C in the autosampler.

Calibration curves for quantification were run 3 times during the injection sequence (beginning, middle, end) to allow monitoring for instrumental drift. The Swedish pooled plasma that was used for method validation and reference standardisation was produced by pooling heparin plasma samples from 953 Swedish individuals (male and female) from the VIP cohort. To support reference standardization, pooled Swedish plasma was run in batches along with individual samples. In this way, analytes discovered in individual samples could be retrospectively semi-quantified by point calibration to the reference pool. Concentrations in the pooled plasma were determined by standard addition for the steroid hormones, or external calibration curves for analytes discovered in suspect screening.

Supplementary Figures

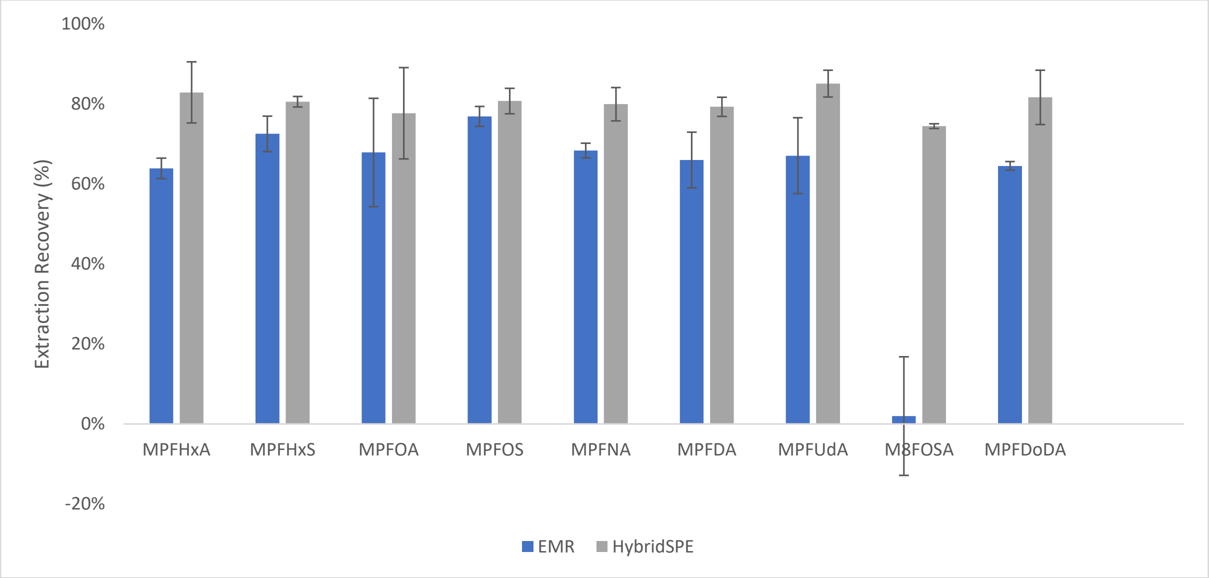

Figure S1. Triplicate extraction recoveries of spiked isotopically-labelled PFAS standards (5 ng/mL) with two commercial lipid removal cartridges: Captiva EMR-Lipid, and HybridSPE-Phospholipid.

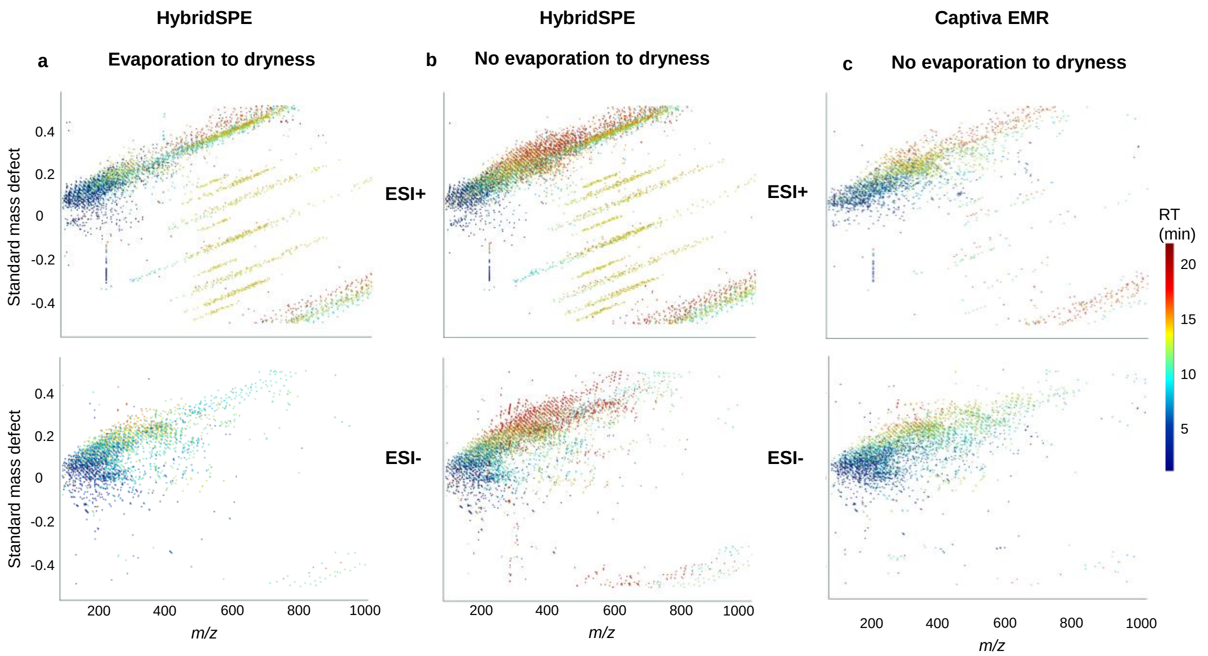

Figure S2. Effect of evaporation to dryness on non-targeted feature coverage. Standard mass defect plots of all non-targeted features in ESI+ and ESI- detected in pooled serum extracts (a) with HybridSPE after evaporation to dryness and reconstitution in 250 µL of 25% methanol (b) with HybridSPE after evaporation to 100 µL and dilution to 250 µL, 25% methanol final composition, or (c) with EMR after evaporation to 100 µL and dilution to 250 µL, 25% methanol final composition. Features are colored by retention time (RT, 1.3-22 min).

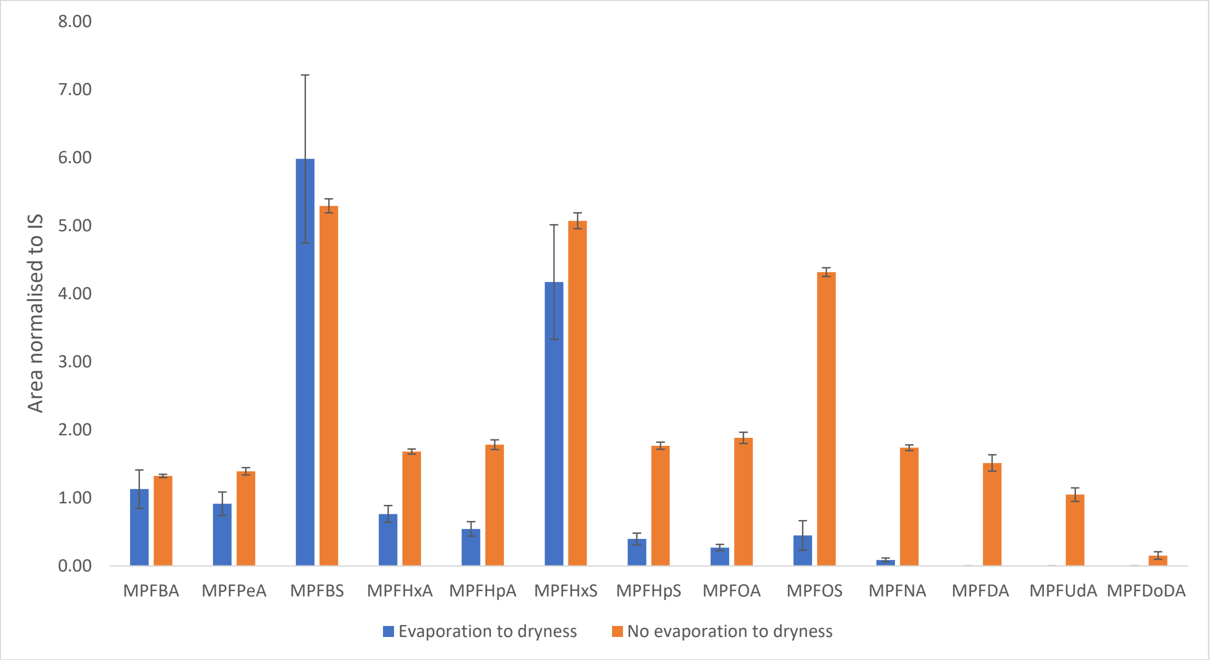

Figure S3. Effect of evaporation to dryness on PFAS targets with HybridSPE. Areas of isotopically-labelled PFAS (spiked before sample preparation at 2 ng/ml) in a treatment with (blue bars) or without (orange bars) evaporation to dryness (n = 3 for each condition).

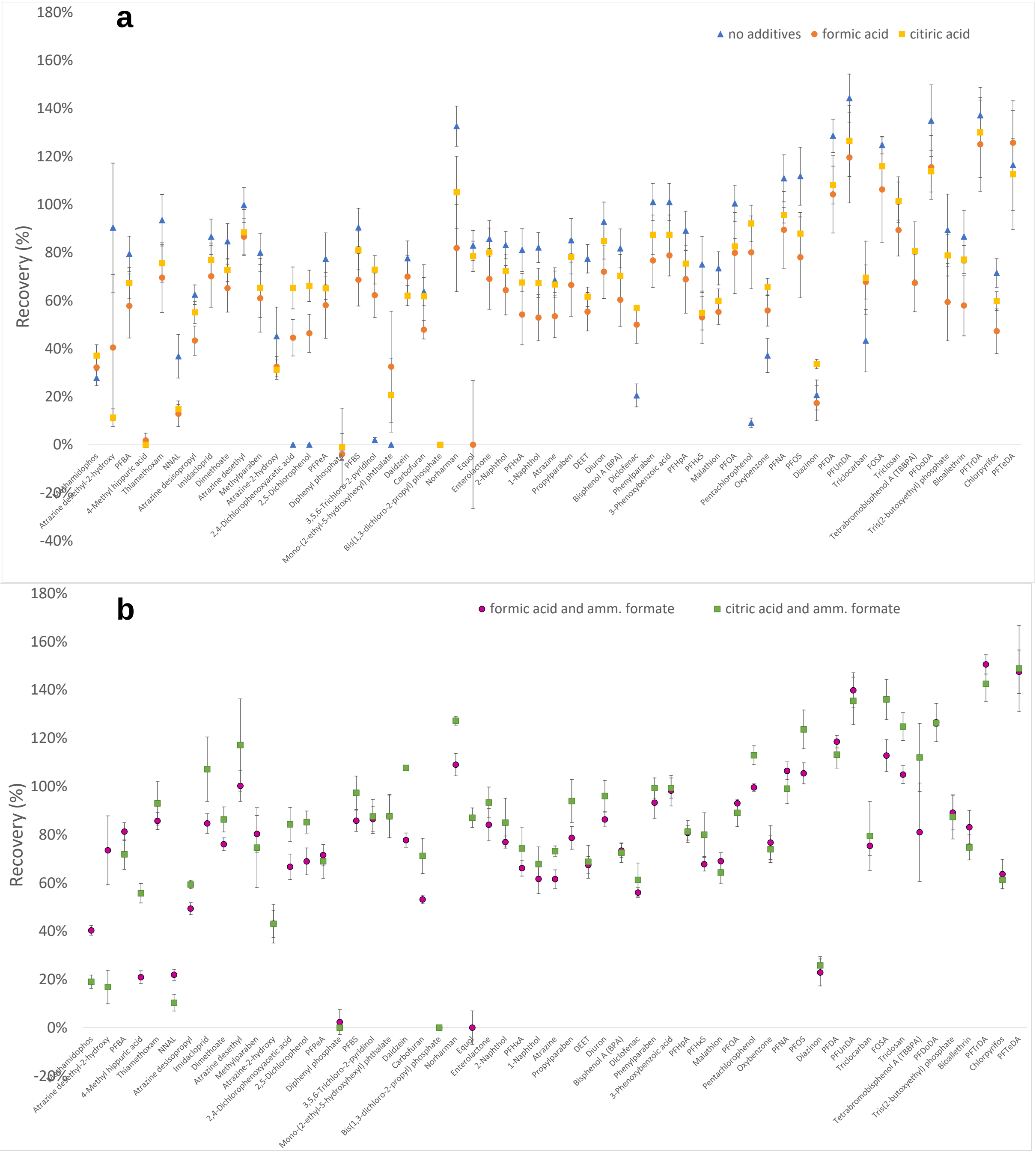

Figure S4. Effect of solvent additives on the recovery of targeted analytes. In panel (a) the tested conditions include no additive (blue triangles), 1% formic acid (orange circles), 0.5% citric acid (yellow squares). In panel (b) the tested conditions are purple circles: formic acid and ammonium formate, green squares: citric acid and ammonium formate (n = 3 for each condition, 5 ng/mL spiking). The analytes on the x - axis are ordered by retention time, from earliest to latest eluting.

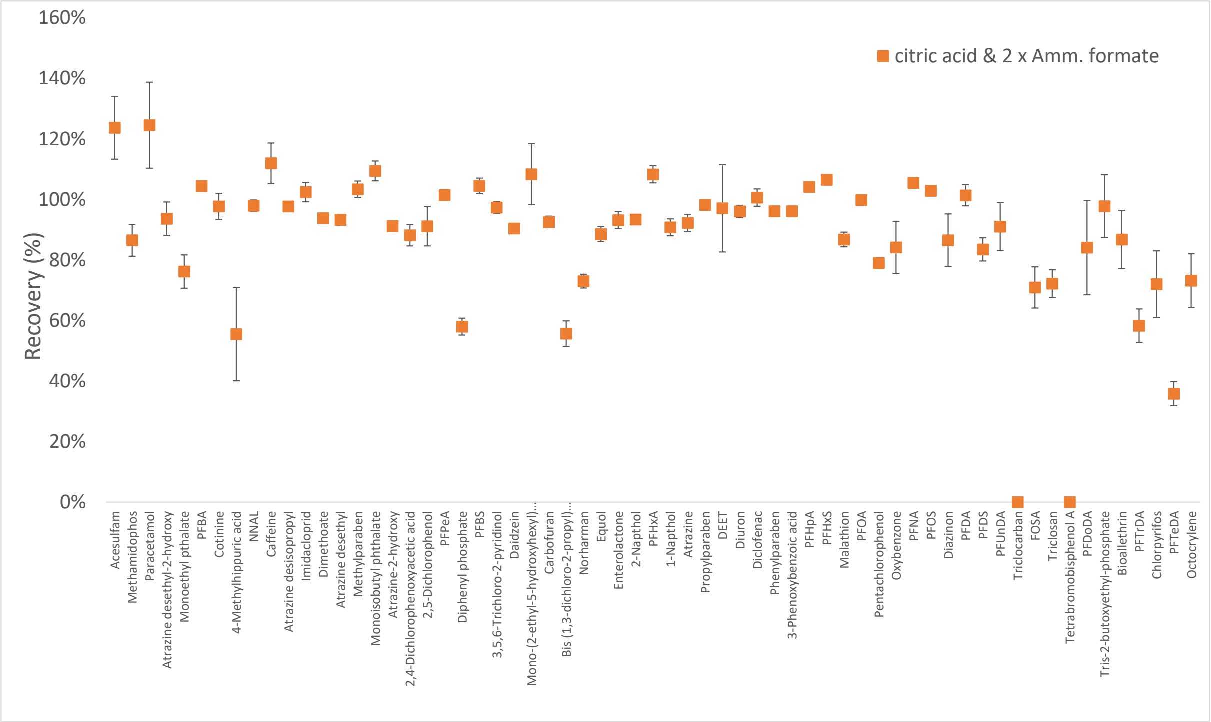

Figure S5. Recoveries of targeted analytes with the final combination of elution solvents and additives. Analyte recoveries after combining citric acid (0.5% in ACN, 1 mL) and ammonium formate (1% in MeOH, 2 mL) for elution are shown (n = 3, 5 ng/mL spiking). The analytes on the x - axis are ordered by retention time, from earliest to latest eluting.

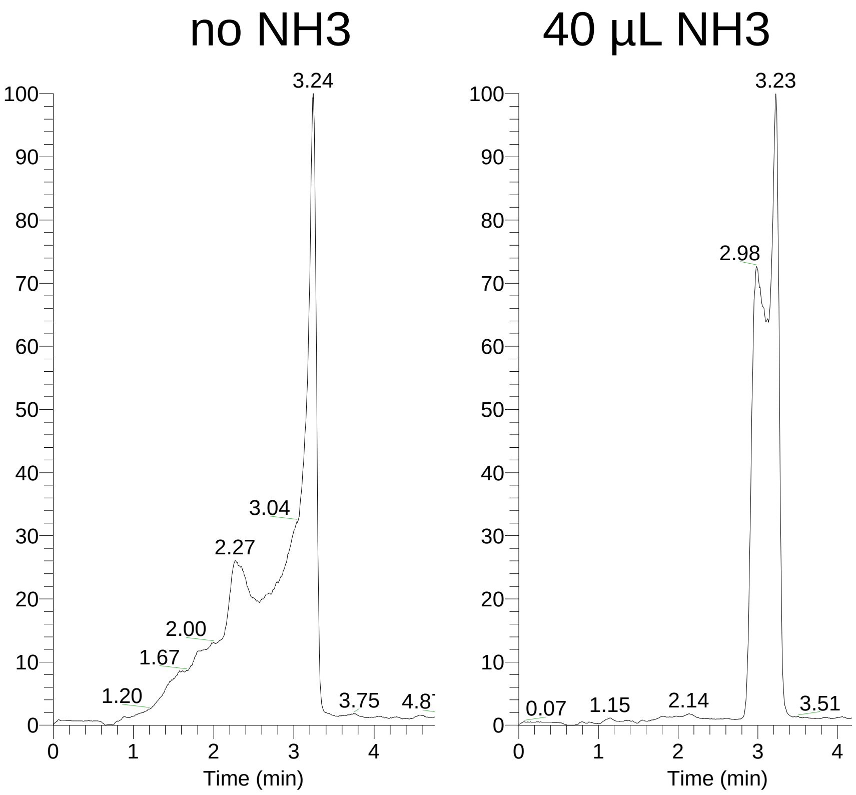

Figure S6. Effect of pH adjustment with ammonia (NH3) solution on peak shapes of early analytes. The pictured peak shapes are of paracetamol before (pH ~ 3) and after (pH ~ 6.5) addition of 40 µL NH3 solution (25%).

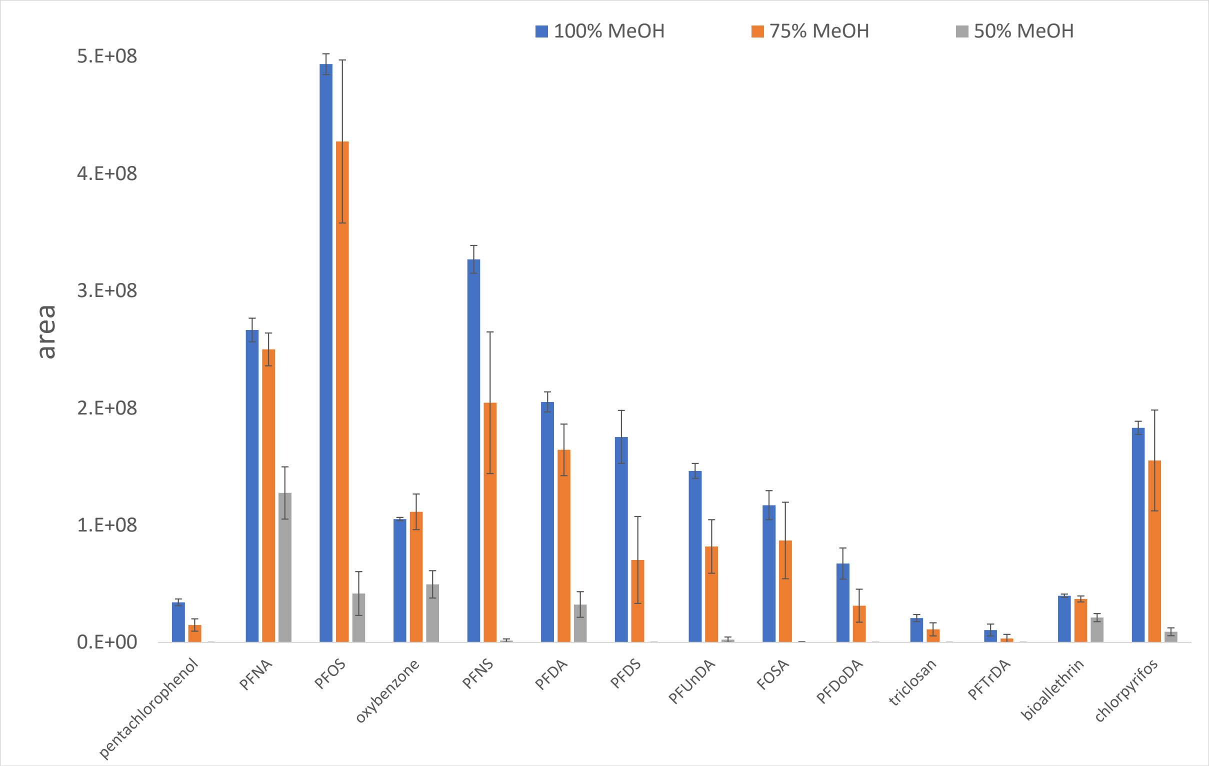

Figure S7. Effect of a final tube washing step on recoveries of hydrophobic or late eluting analytes. Analyte peak areas of relatively late eluting analytes are shown after applying a 100 µL wash with 100% MeOH (blue), 75% MeOH in water (orange) or 50% MeOH in water (grey). The analytes were spiked before sample preparation at 5 ng/mL. The analytes on the x - axis are ordered by retention time, from earliest to latest eluting.

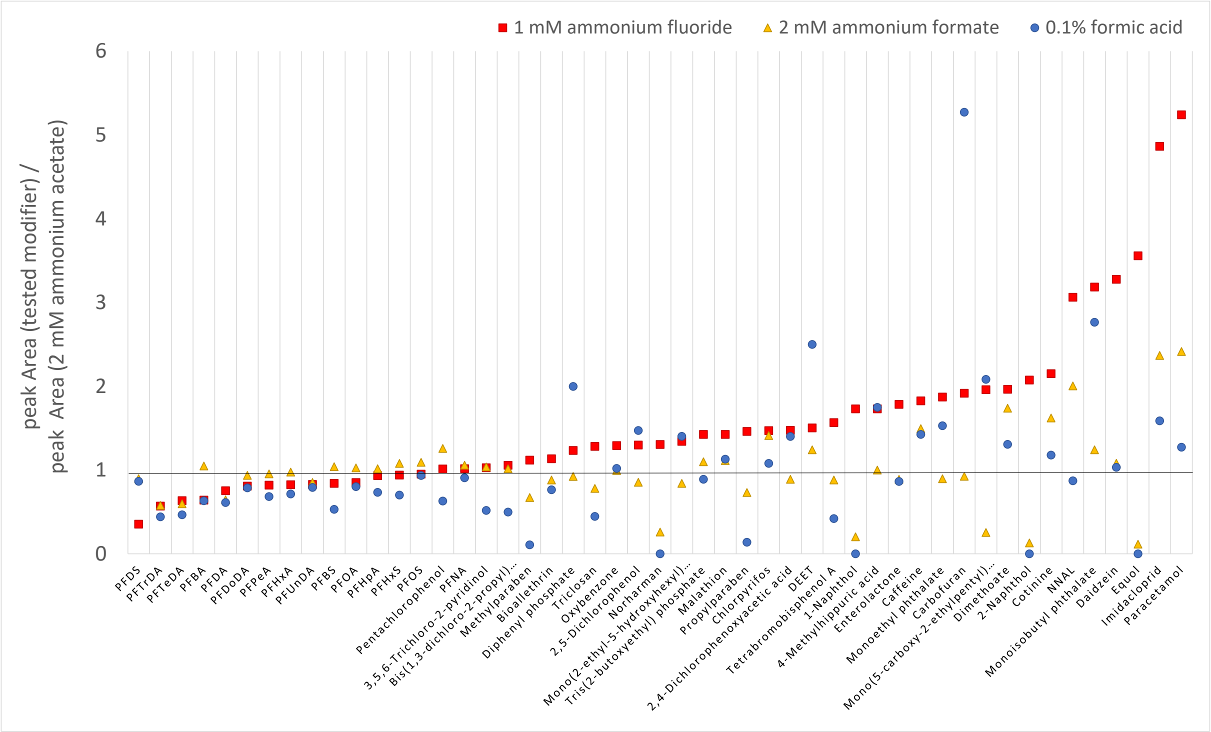

Figure S8. Effect of mobile phase A modifiers on the sensitivity of targeted analyte detection. Comparison of modifiers is shown in form of ratios of the peak areas obtained with 1 mM ammonium fluoride (red squares), 2 mM ammonium formate (yellow triangles) and 0.1% formic acid (blue circles) to the peak areas obtained with 2 mM ammonium acetate (black line). The analytes for this test were spiked in solvent at 5 ng/mL.

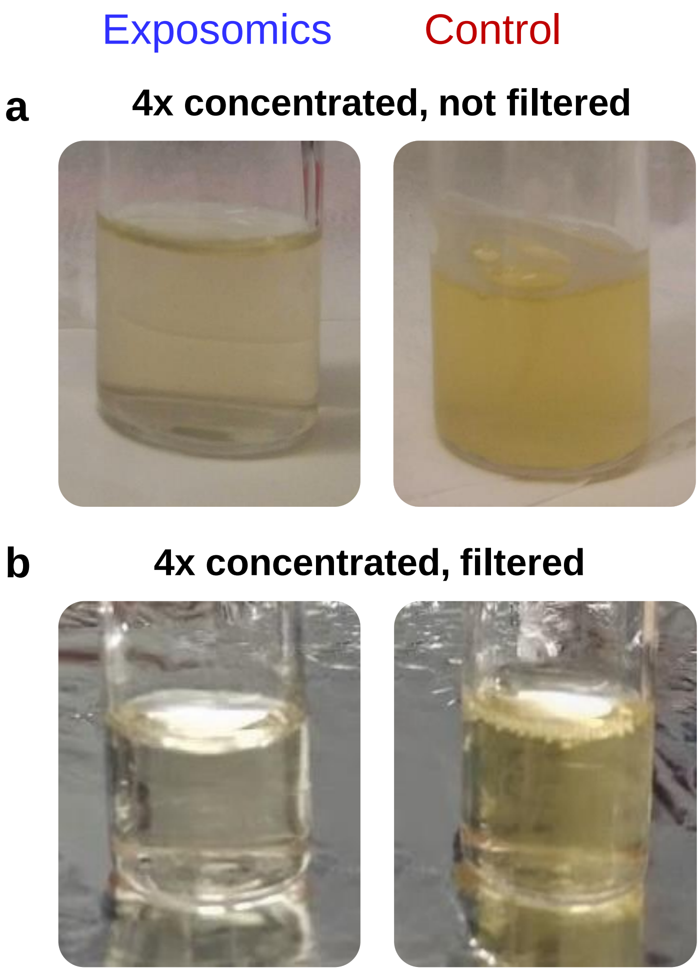

Figure S9. Comparison of concentrated exposomics and control extracts. Visual comparison of exposomics and control extracts after 4x concentration (a) before filtration and (b) after filtration. Precipitation was visible in the control extracts before filtration.

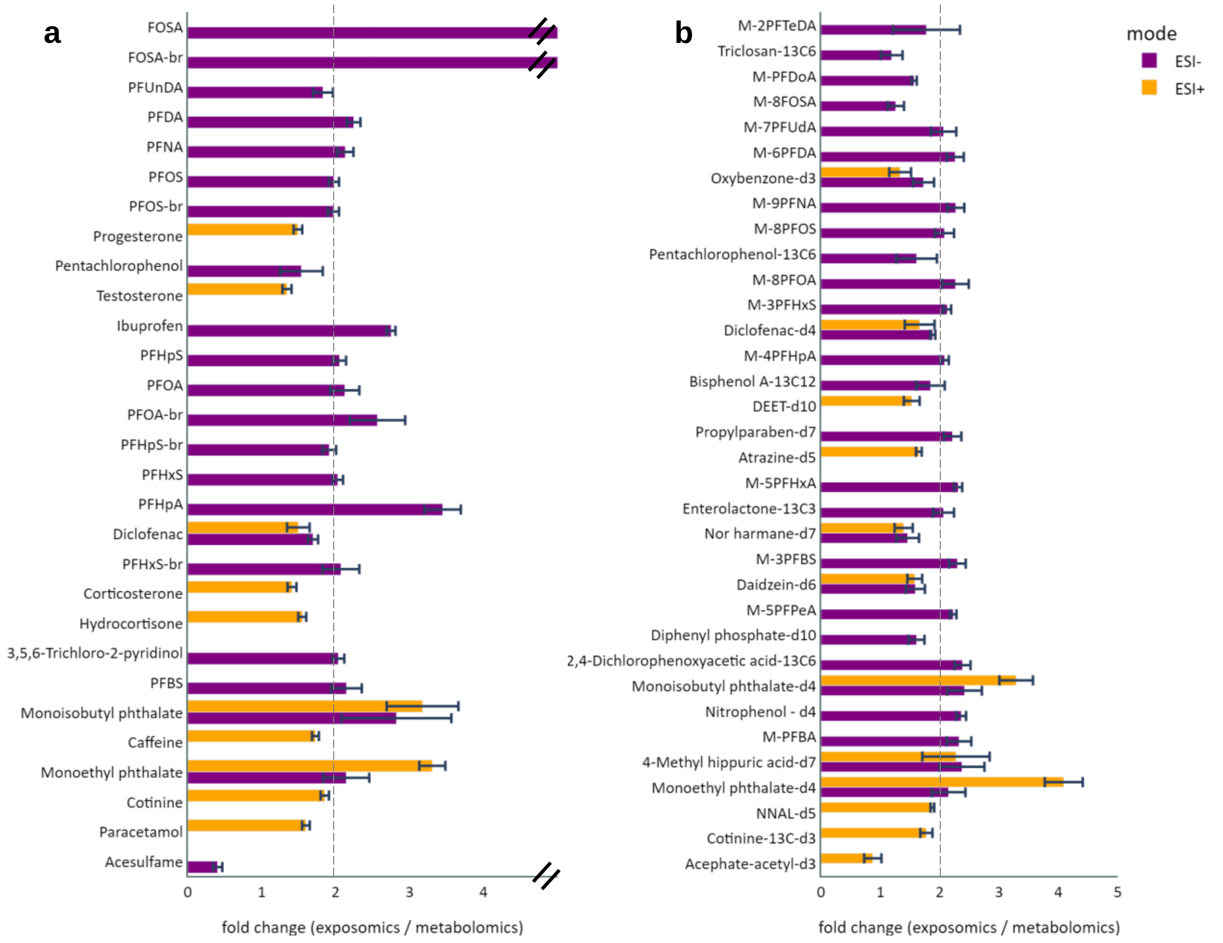

Figure S10. Comparison of detected targeted analytes and spiked internal standards in Swedish pooled plasma between the exposomics and the control protocols. (a) Detected (not spiked) targeted and (b) Isotopically-labelled (spiked) raw analyte peak areas expressed as a ratio between the exposomics and the control protocols in ESI- (purple) and ESI+ (orange)**.** Spiked standards were added before sample preparation. Areas for the sum of branched isomers of PFAS analytes were considered separately when detected. The dashed line indicates the fold change threshold of 2, as expected by the difference in injection volumes for the exposomics (20 µL) and control (10 µL) protocols. Analytes are ordered by retention time from bottom to top in each panel. The symbol // for FOSA and its branched isomers (FOSA-br) indicates that the analytes were only at detectable by the exposomics protocol.

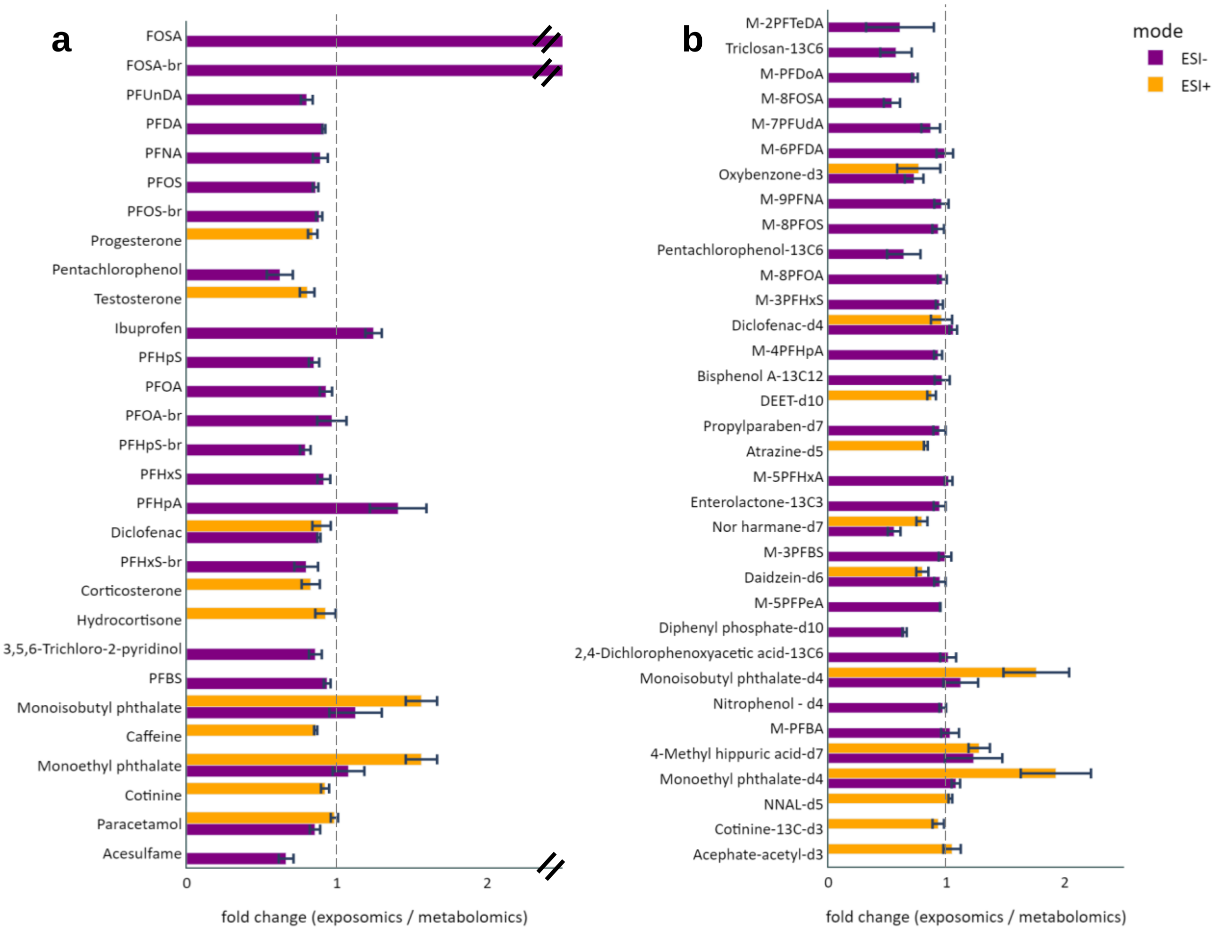

Figure S11. Comparison of detected targeted analytes and spiked internal standards in Swedish pooled plasma between the exposomics extracts spiked at 10 µL and the control extracts. (a) Detected (not spiked) targeted and (b) Isotopically-labelled (spiked) raw analyte peak areas expressed as a ratio between the exposomics (10 µL injection) and the control protocols in ESI- (purple) and ESI+ (orange). Spiked standards were added before sample preparation. Areas for the sum of branched isomers of PFAS analytes were considered separately when detected. The dashed line indicates the fold change threshold of 1, as expected by injecting the two types of extracts at the same volume. Analytes are ordered by retention time from bottom to top in each panel. The symbol // for FOSA and its branched isomers (FOSA-br) indicates that the analytes were only at detectable in the exposomics extracts.

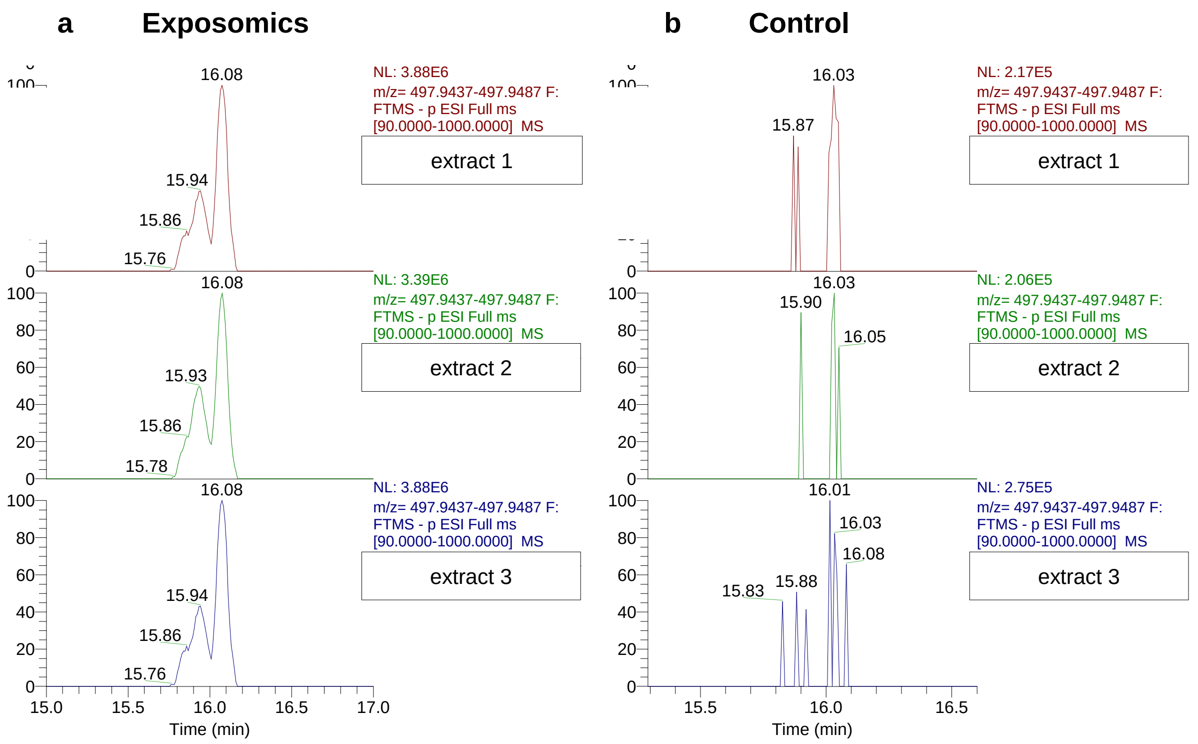

Figure S12. Comparison of the targeted analyte FOSA in Swedish pooled plasma between the exposomics and the contol protocols. Extracted ion chromatogram of FOSA (detected, not spiked) in Swedish pooled plasma (a) extracted with the exposomics method and (b) extracted with the control method. In the exposomics extracts the sum of branched isomers for FOSA elute at an earlier retention time (15.94 min).

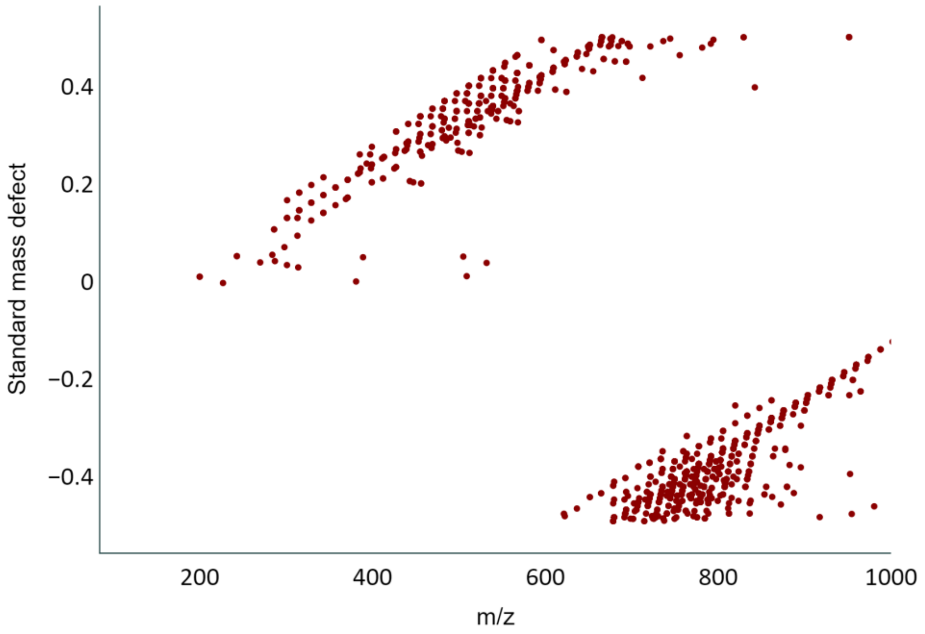

Figure S13. Chemical space of phospholipids depicted in a standard mass defect plot. Compounds categorised as glycerophospholipids from the LIPID MAPS[7] database are plotted (2008 version, n = 1915). The $[M+H]^+$ ion of the compound masses was used for mass defect calculations. Curated excel file downloaded from fiehnlab.ucdavis.edu[8]

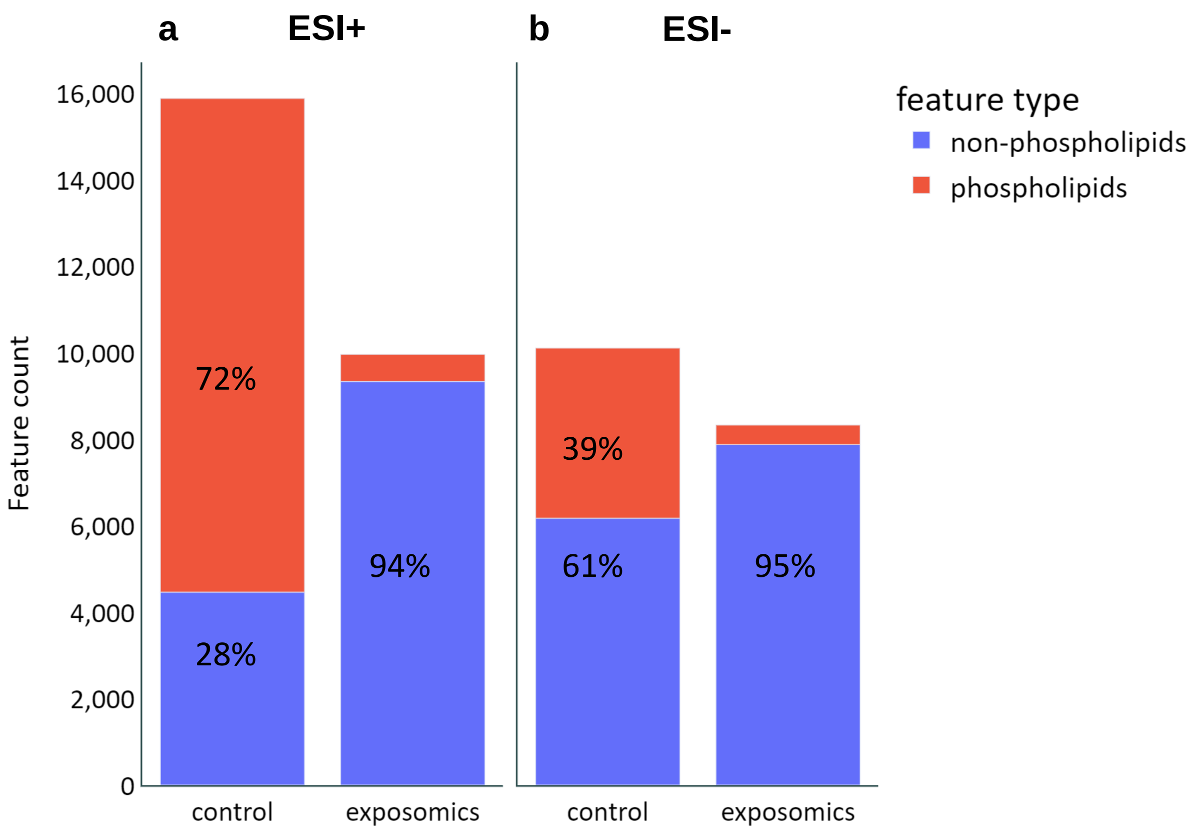

Figure S14. Feature count for phospholipid and non-phospholipid features detected by the exposomics and the control protocols in (a) ESI+ and (b) ESI-. Features are classified as "phospholipids" and "non-phospholipids" based on the presence or absence of phospholipid diagnostic fragment ions.

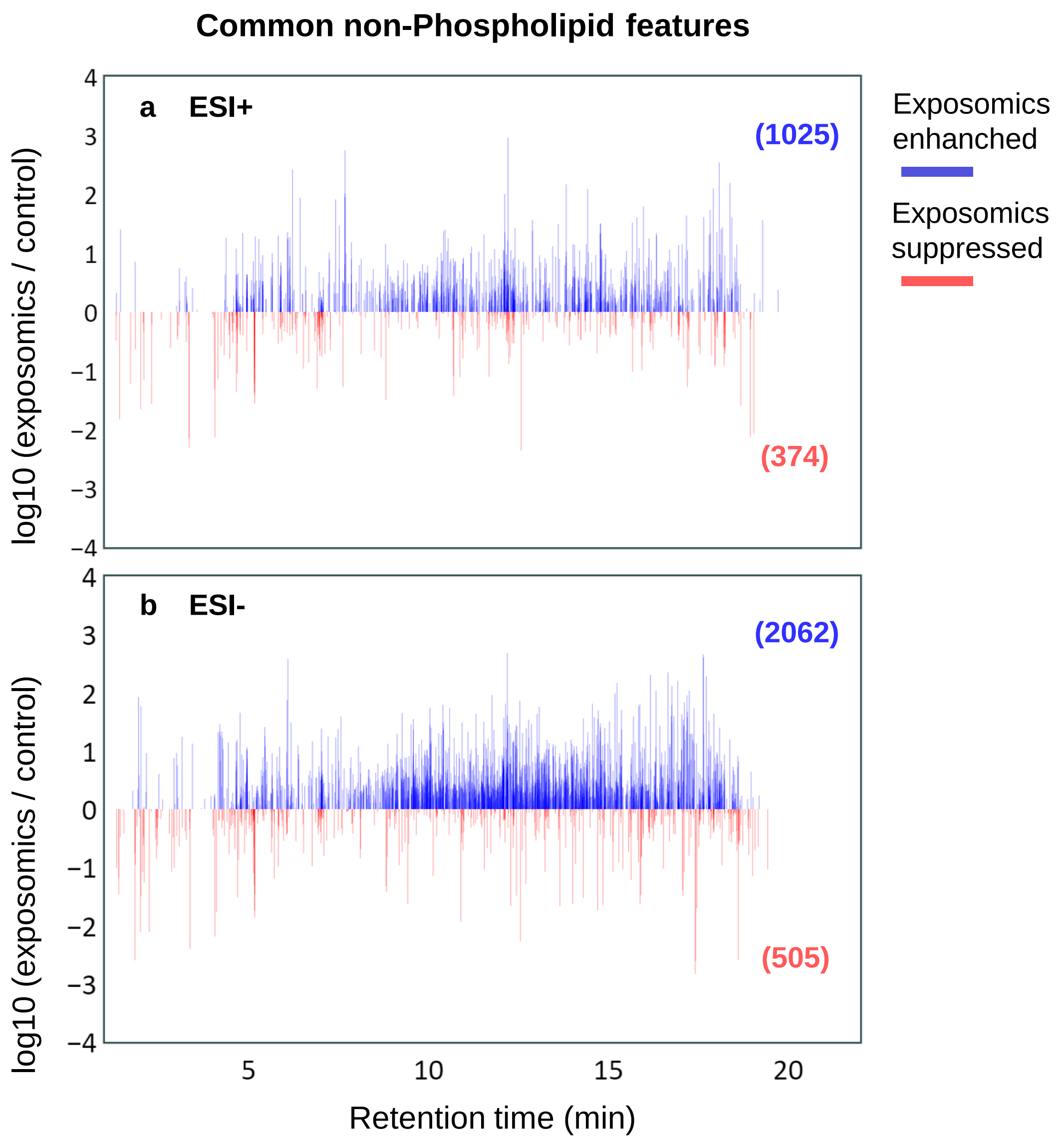

Figure S15. Comparison of areas of common non-phospholipid features between the exposomics and control protocols. Logged 10 values of fold changes (i.e., area ratio between exposomics and the control method) are presented on the y-axis for (a) ESI+ and (b) ESI-. Numbers in brackets in blue and red font are features enhanced and suppressed in exposomics extracts respectively.

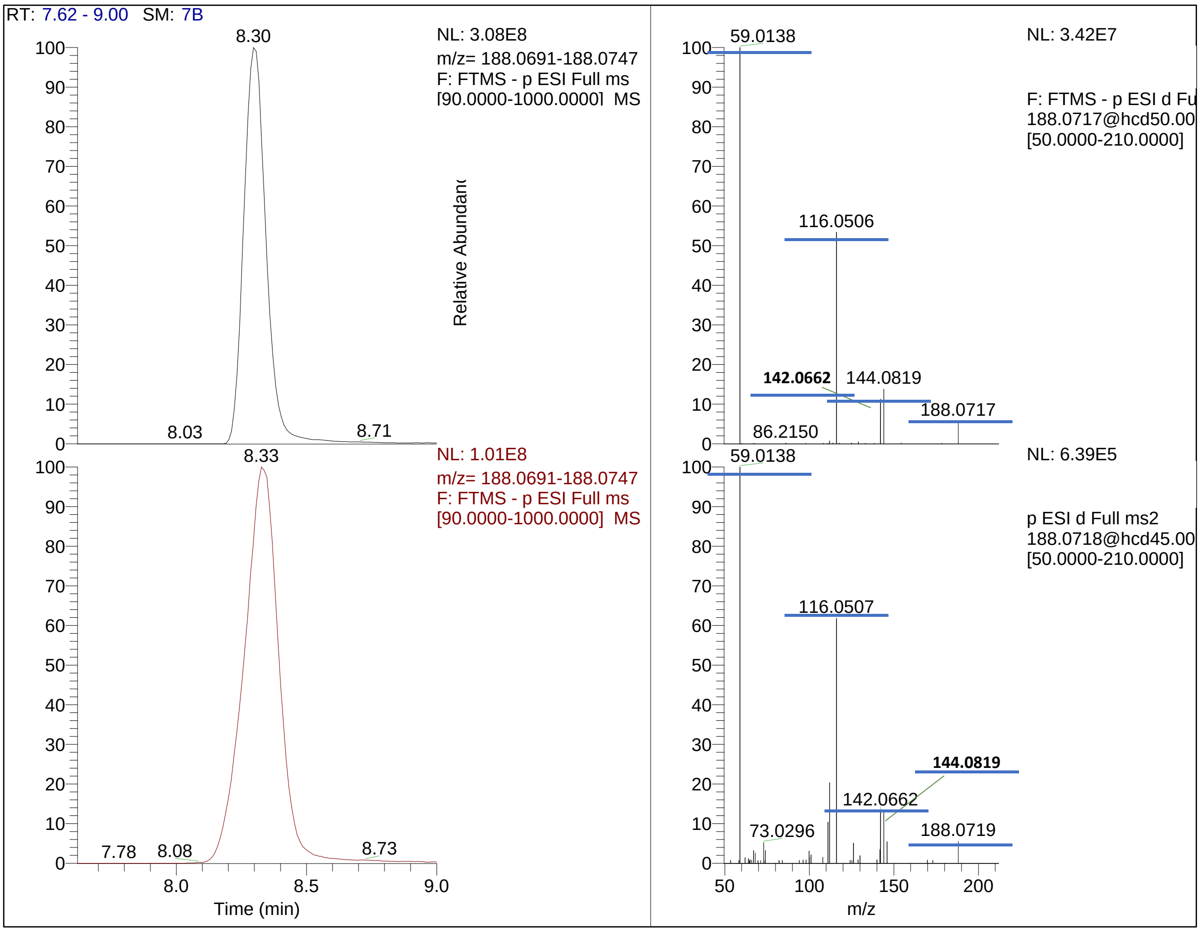

Figure S16. Extracted ion chromatogram (left) and data dependent acquisition (DDA) spectrum (right) for indolepropionic acid in pooled Swedish plasma (top) and spiked pooled plasma (bottom). MS2 ions specific to the precursor are underlined in blue (source: https://mona.fiehnlab.ucdavis.edu/spectra/display/MoNA038180).

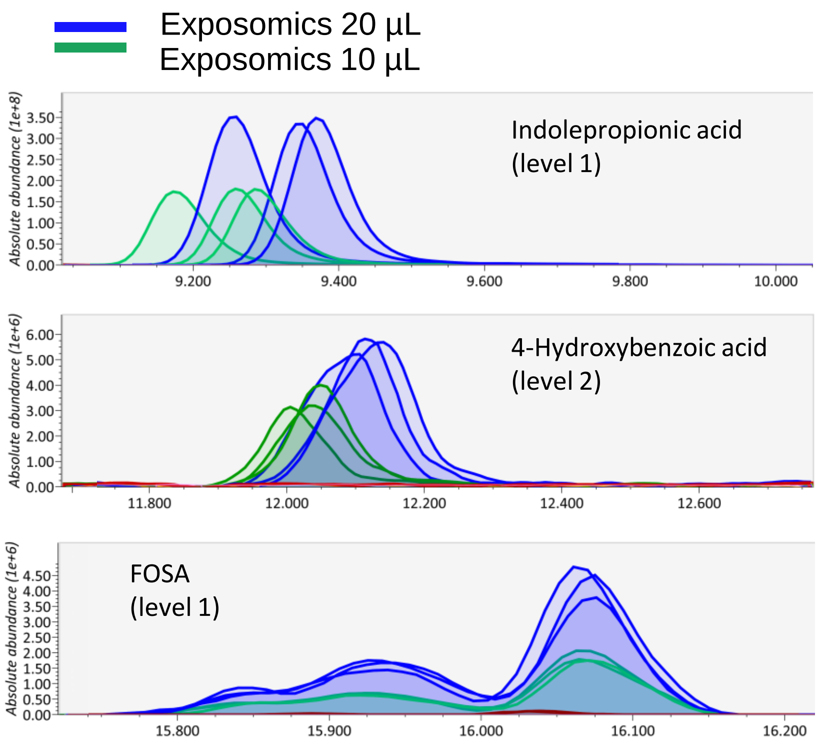

Figure S17. Exposomics “new features” detected at 10 µL injection volume. Extracted chromatograms for features detectable by exposomics (both 10 and 20 µL injection) but not by the control method. For corresponding library spectral matches see Figure 3d.

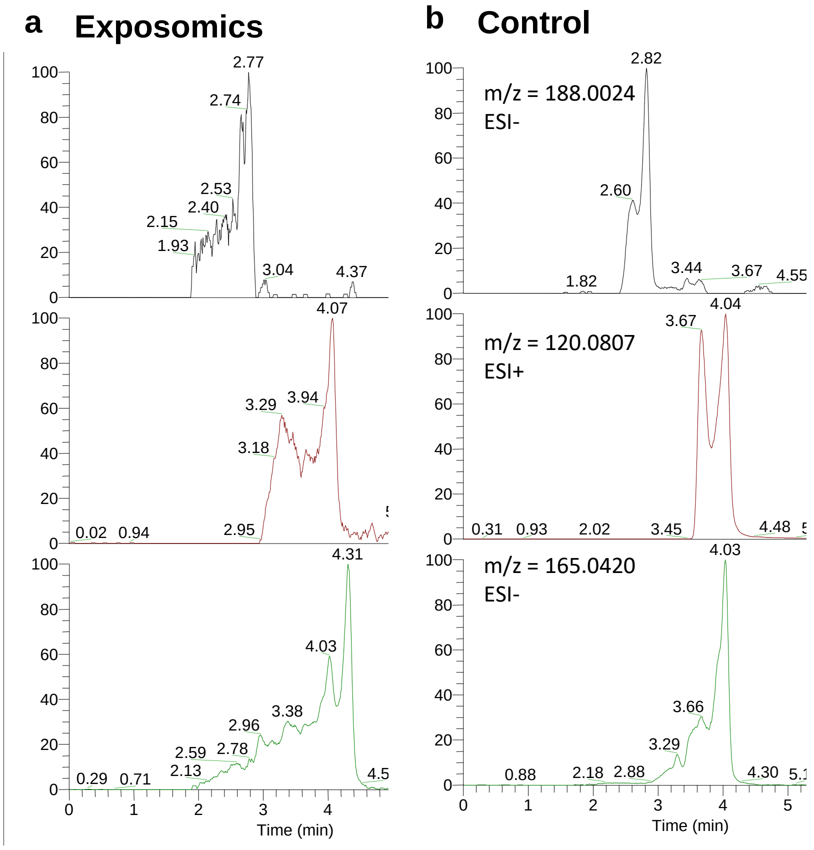

Figure S18. Peak broadening (fronting) examples for early eluting non-targeted features in the exposomics protocol. Examples of peak shapes are of early eluting non-targeted features for (a) the exposomics and (b) the control protocol, which elute outside the retention time range of the targeted analytes.

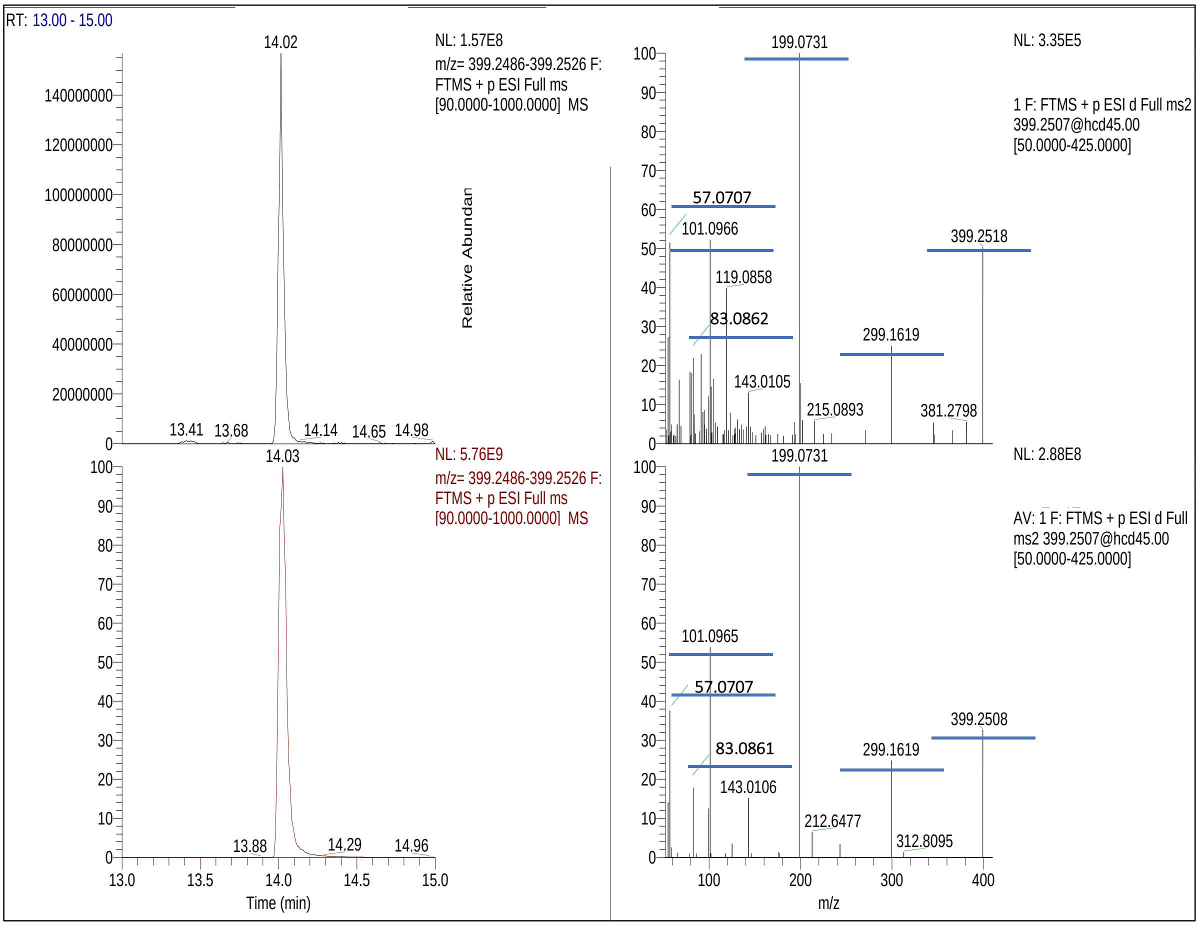

Figure S19. Extracted ion chromatogram (left) and data dependent acquisition (DDA) spectrum (right) for tris-2-butoxyethyl-phosphate in Individual Swedish plasma (top) and standard solution (bottom). MS2 ions specific to the precursor are underlined in blue (source: https://massbank.eu/MassBank/RecordDisplay?id=MSBNK-Antwerp_Univ AN118001&dsn=Antwerp_Univ).

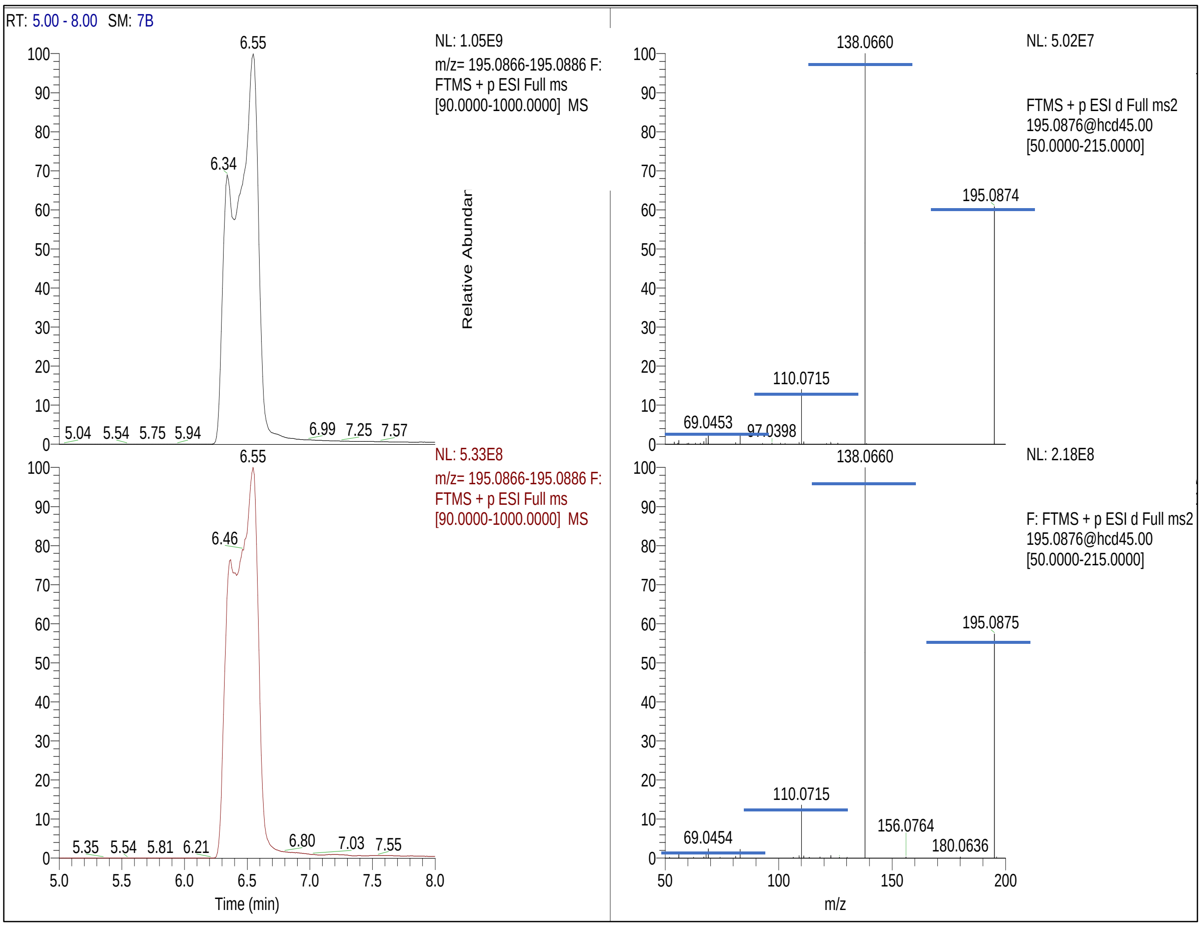

Figure S20. Extracted ion chromatogram (left) and data dependent acquisition (DDA) spectrum (right) for caffeine in pooled Swedish sample (top) and standard solution (bottom). MS2 ions specific to the precursor are underlined in blue (source: https://massbank.eu/MassBank/RecordDisplay?id=MSBNK-Eawag-EA030312&dsn=Eawag).

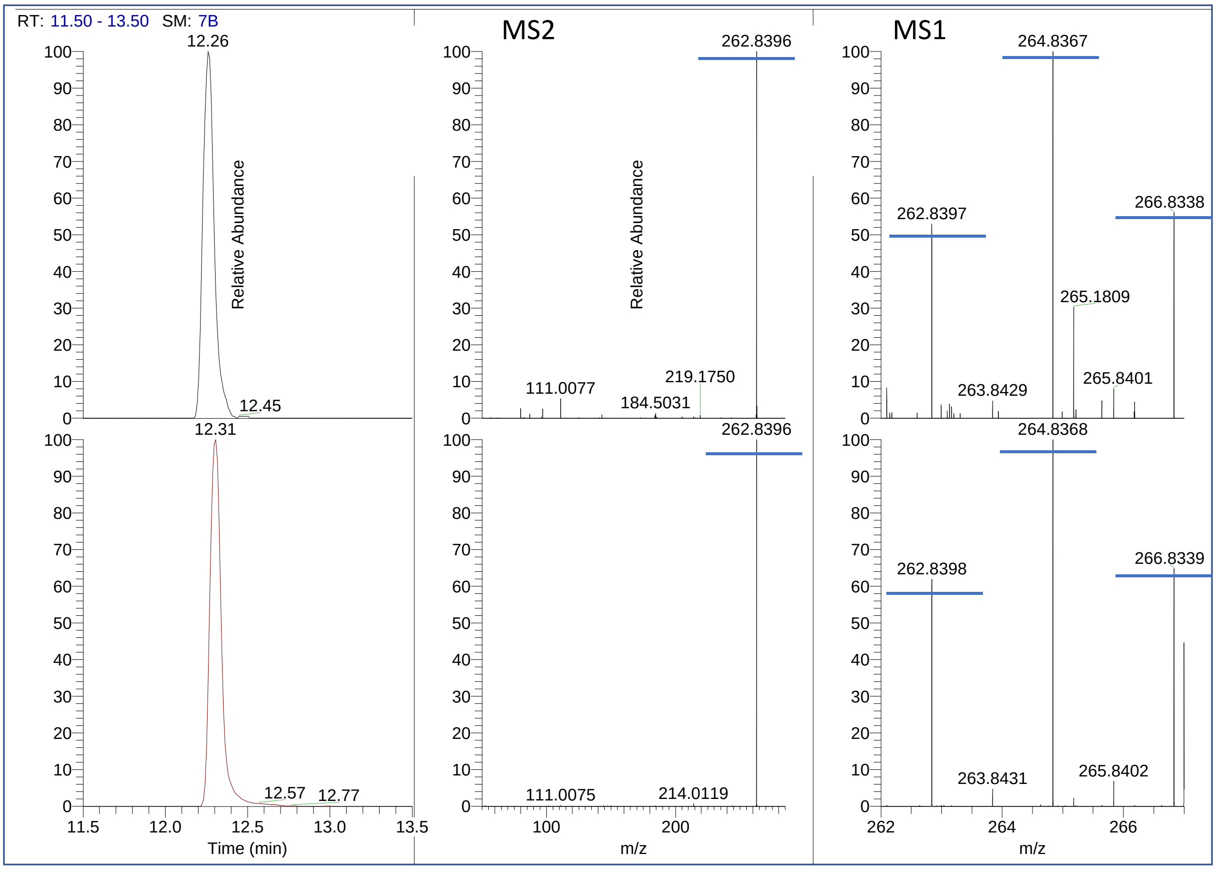

Figure S21. Extracted ion chromatogram (left) and data dependent acquisition (DDA) spectrum (middle) as well as MS1 acquisition (right) for pentachlorophenol in pooled Swedish sample (top) and spiked pooled plasma (bottom). The isotopic pattern of the pentachlorophenol molecular ion at MS1 is underlined in blue (ratio of the isotope peaks of 263 : 265 : 267 = 9 : 15 : 10).[9] MS2 ions specific to the precursor are underlined in blue (source: https://massbank.eu/MassBank/RecordDisplay?id=MSBNK-Eawag-EQ371851&dsn=Eawag).

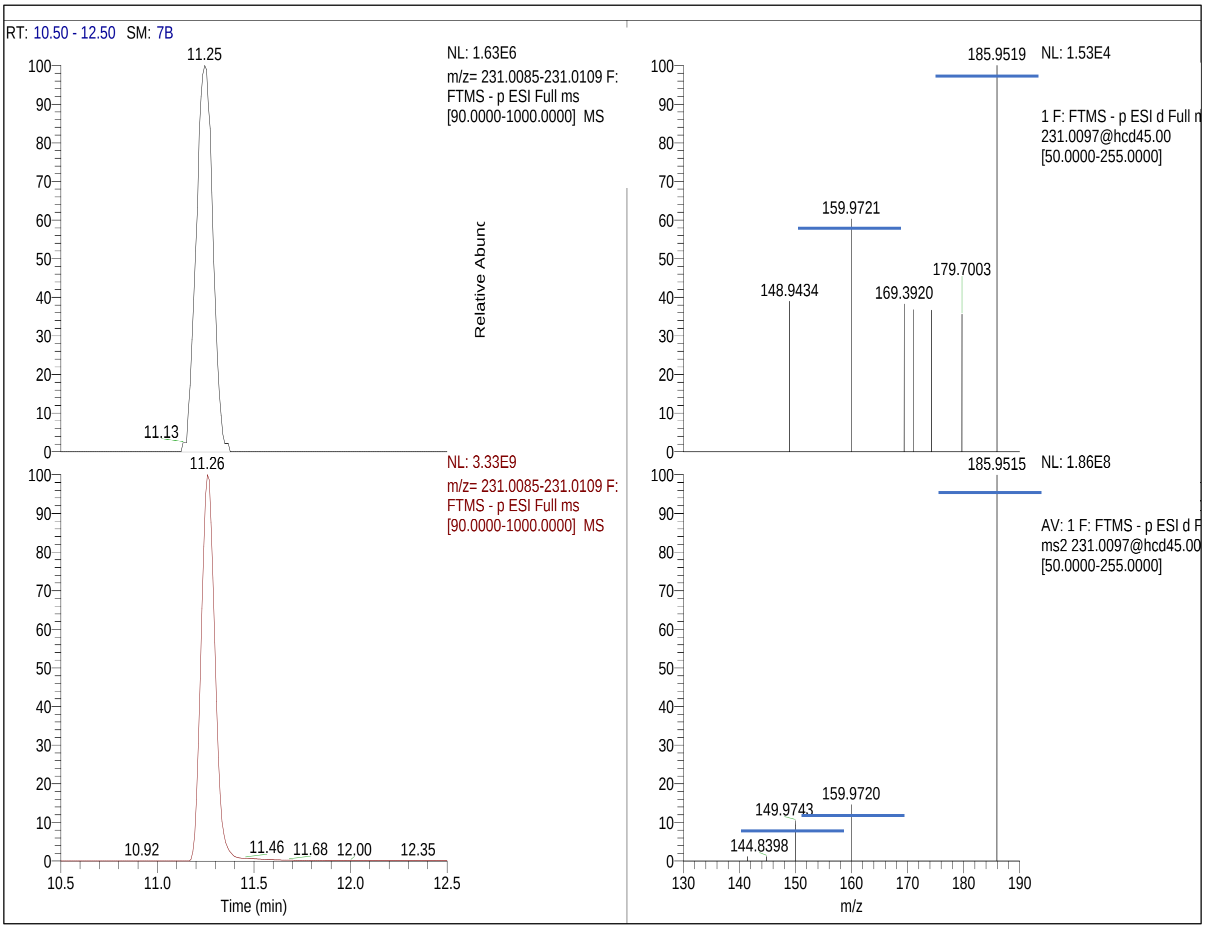

Figure S22. Extracted ion chromatogram (left) and data dependent acquisition (DDA) spectrum (right) for diuron in Individual Swedish plasma (top) and standard solution (bottom). MS2 ions specific to the precursor are underlined in blue (source: https://massbank.eu/MassBank/RecordDisplay?id=MSBNK-Eawag-EA029260&dsn=Eawag).

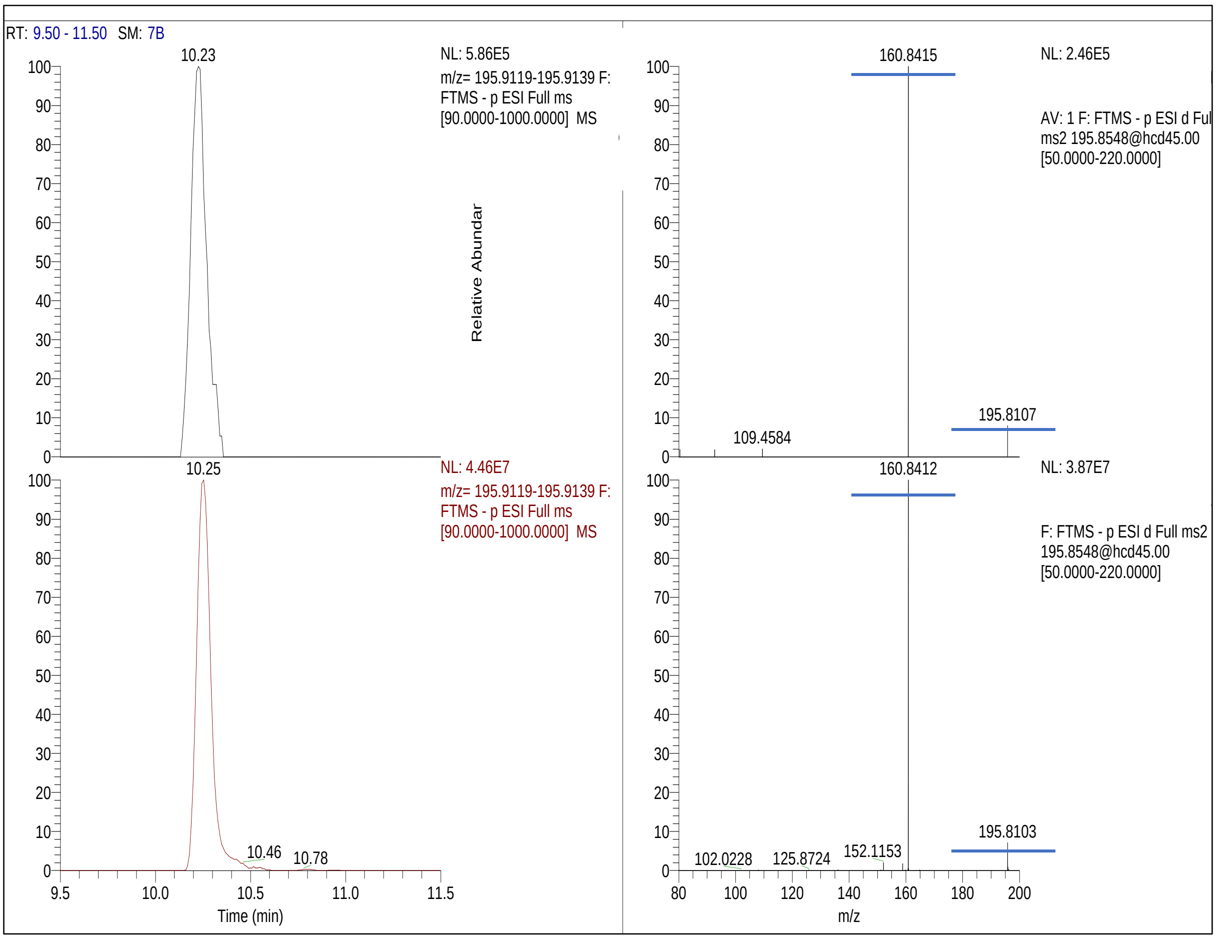

Figure S23. Extracted ion chromatogram (left) and data dependent acquisition (DDA) spectrum (right) for 3,5,6-trichloro-2-pyridinol in Individual Swedish plasma (top) and spiked pooled plasma (bottom). MS2 ions specific to the precursor are underlined in blue (source: https://massbank.eu/MassBank/RecordDisplay?id=MSBNK-Eawag-EA270451&dsn=Eawag).

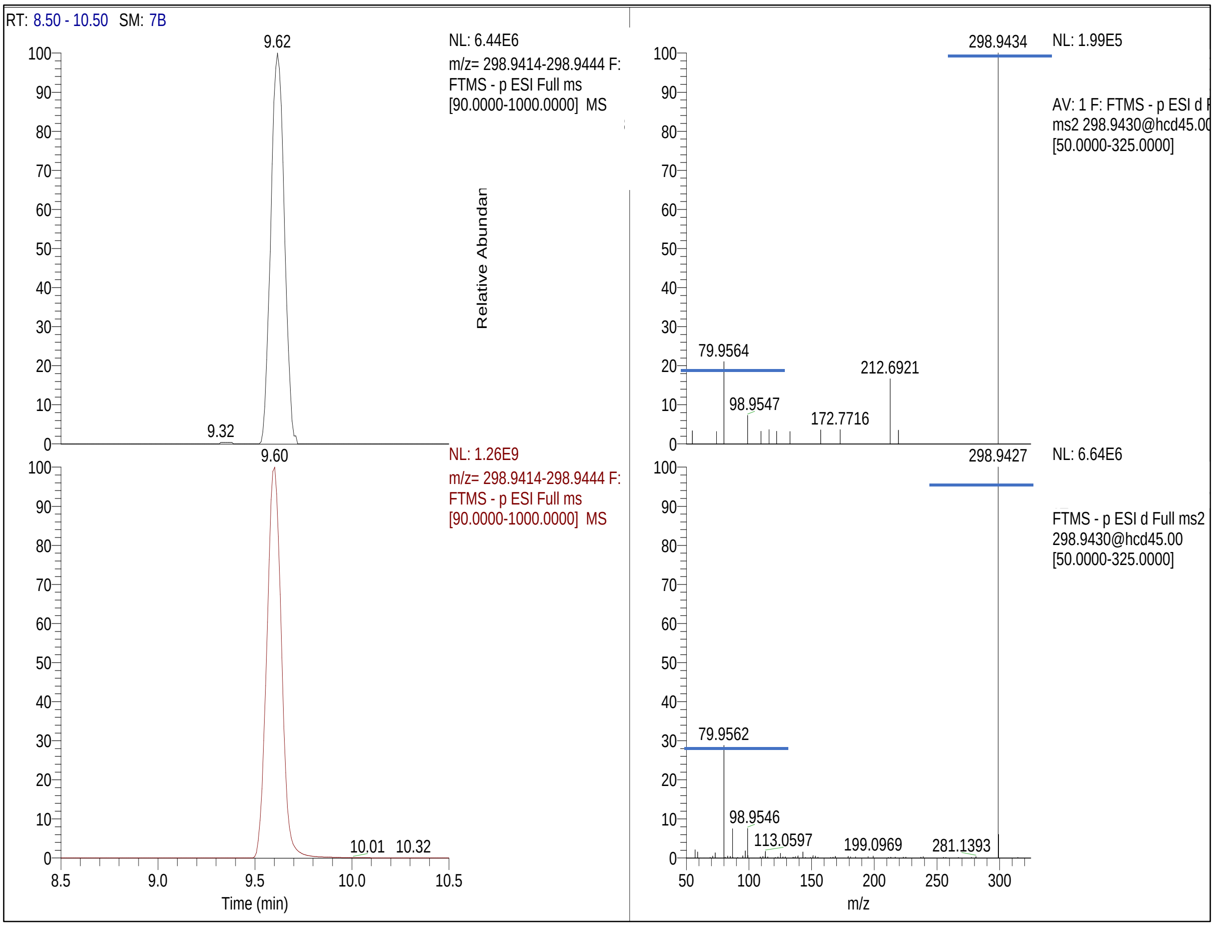

Figure S24. Extracted ion chromatogram (left) and data dependent acquisition (DDA) spectrum (right) for PFBS in pooled Swedish plasma (top) and standard solution (bottom). MS2 ions specific to the precursor are underlined in blue (source: https://mona.fiehnlab.ucdavis.edu/spectra/display/EQ367551).

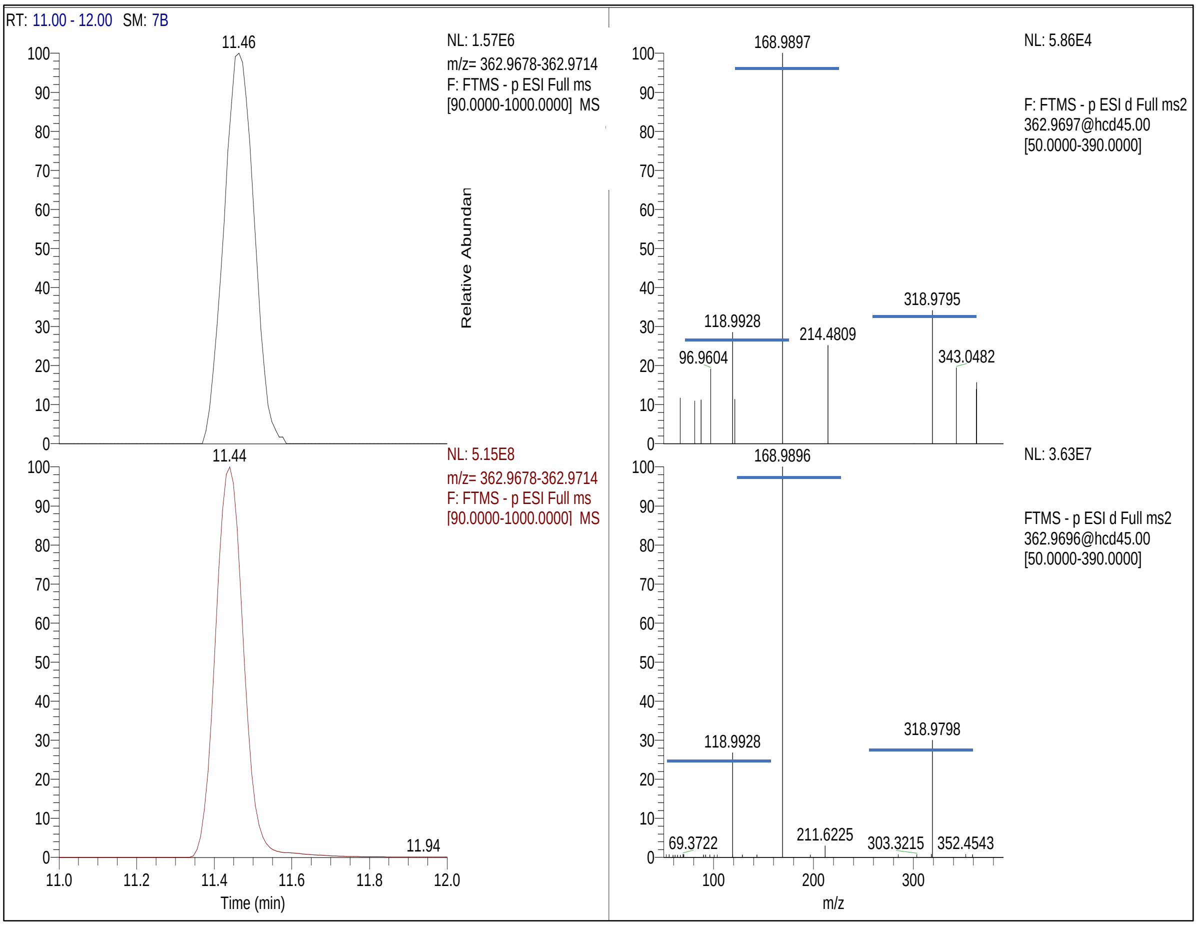

Figure S25. Extracted ion chromatogram (left) and data dependent acquisition (DDA) spectrum (right) for PFHpA in pooled Swedish plasma (top) and standard solution (bottom). MS2 ions specific to the precursor are underlined in blue (source: https://massbank.eu/MassBank/RecordDisplay?id=MSBNK-Athens_UnivAU594751&dsn=Athens_Univ).

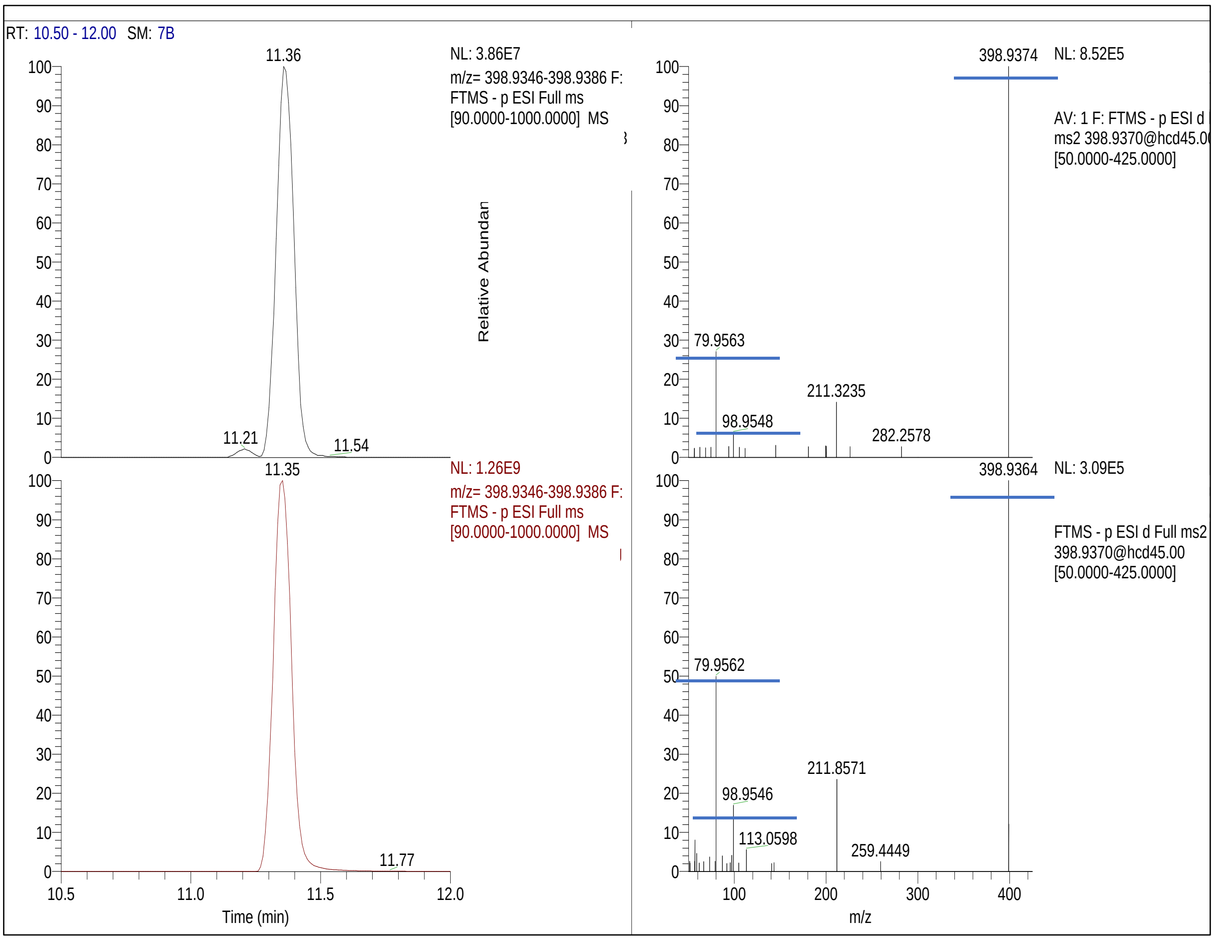

Figure S26. Extracted ion chromatogram (left) and data dependent acquisition (DDA) spectrum (right) for PFHxS in pooled Swedish plasma (top) and standard solution (bottom). MS2 ions specific to the precursor are underlined in blue (source: https://massbank.eu/MassBank/RecordDisplay?id=MSBNK-ACES_SU-AS000010&dsn=ACES_SU).

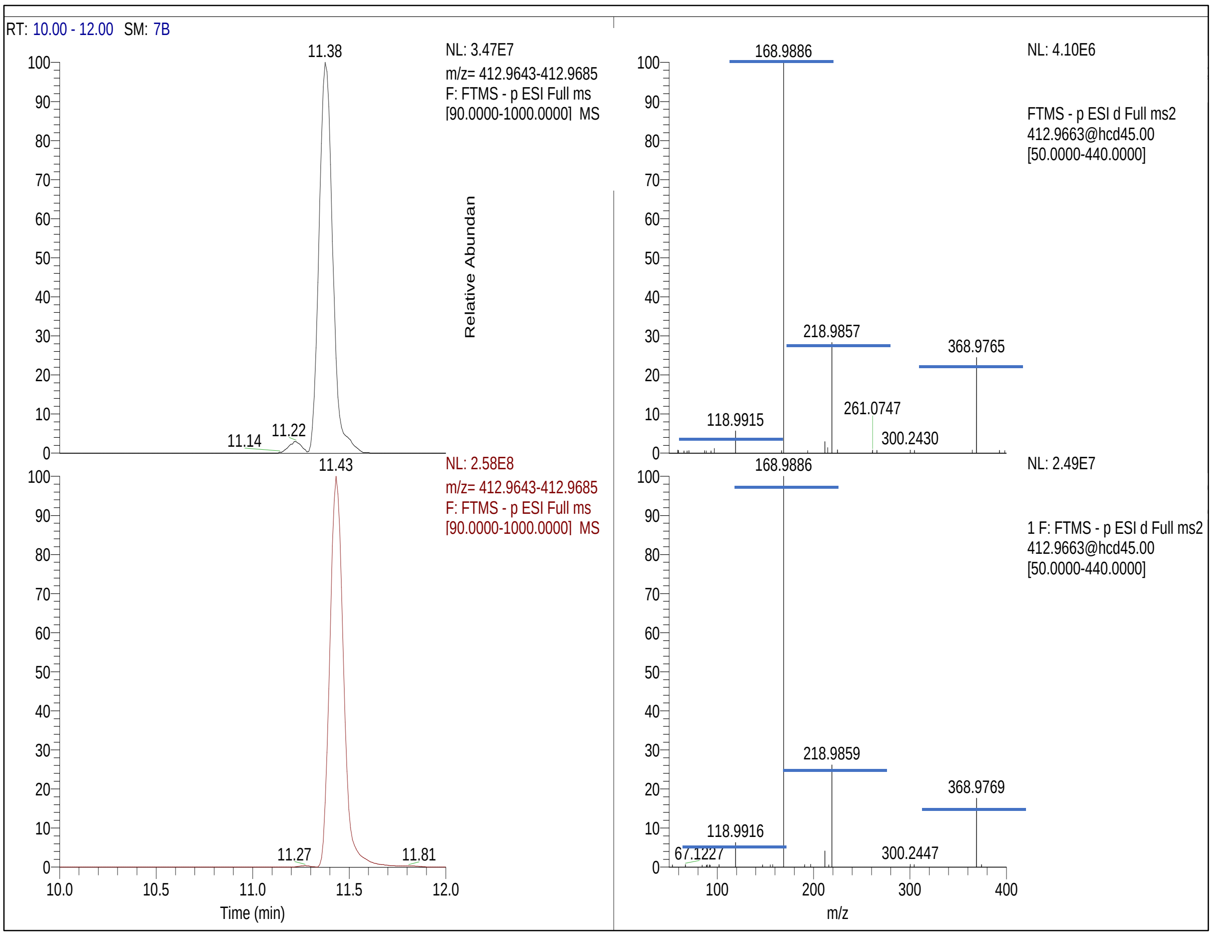

Figure S27. Extracted ion chromatogram (left) and data dependent acquisition (DDA) spectrum (right) for PFOA in pooled Swedish plasma (top) and spiked pooled plasma (bottom). MS2 ions specific to the precursor are underlined in blue (source: https://mona.fiehnlab.ucdavis.edu/spectra/display/MoNA024627). Notice that green lines do not show ion intensity but rather clarify m/z values of ions they point to.

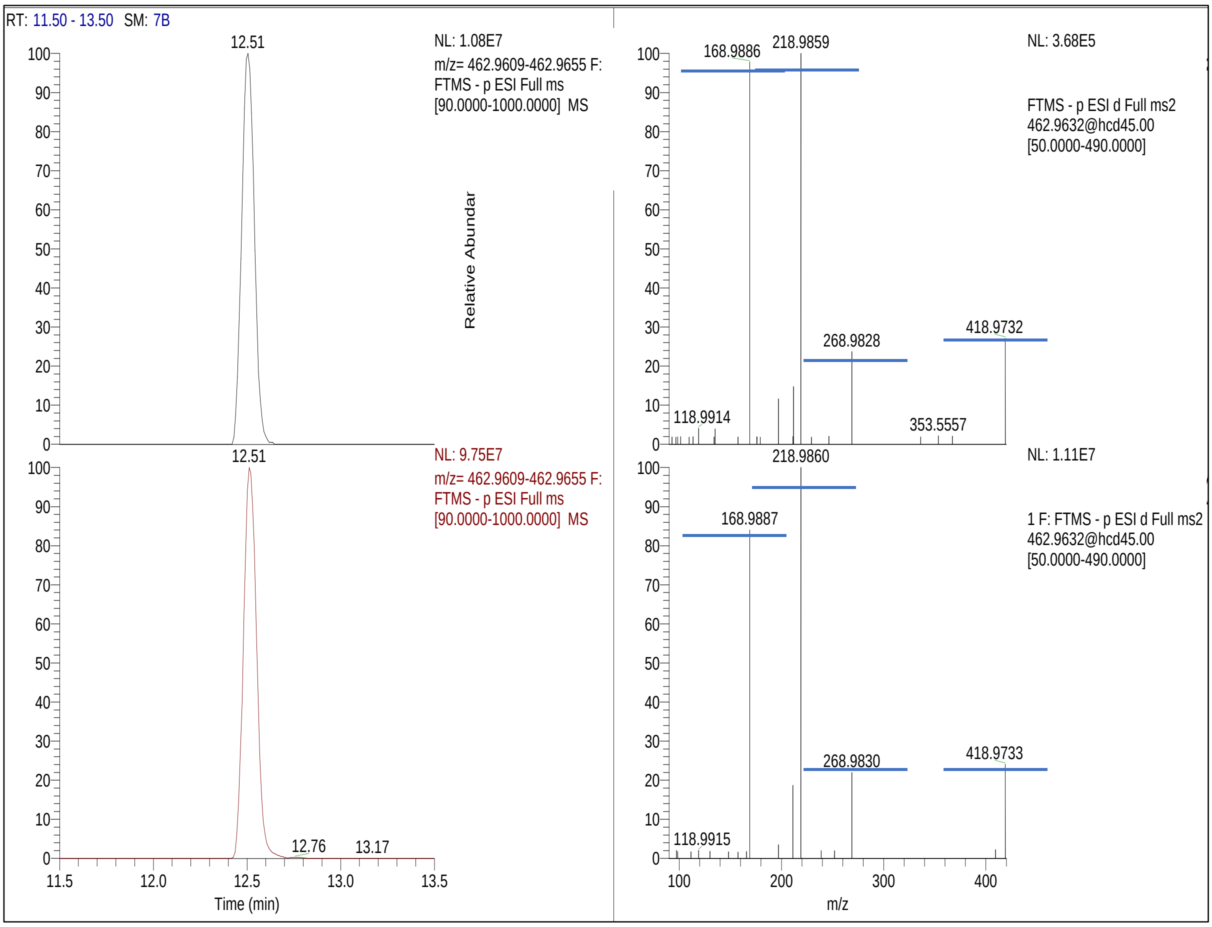

Figure S28. Extracted ion chromatogram (left) and data dependent acquisition (DDA) spectrum (right) for PFNA in pooled Swedish plasma (top) and spiked pooled plasma (bottom). MS2 ions specific to the precursor are underlined in blue (source: https://massbank.eu/MassBank/RecordDisplay?id=MSBNK-Eawag-EA271858&dsn=Eawag).

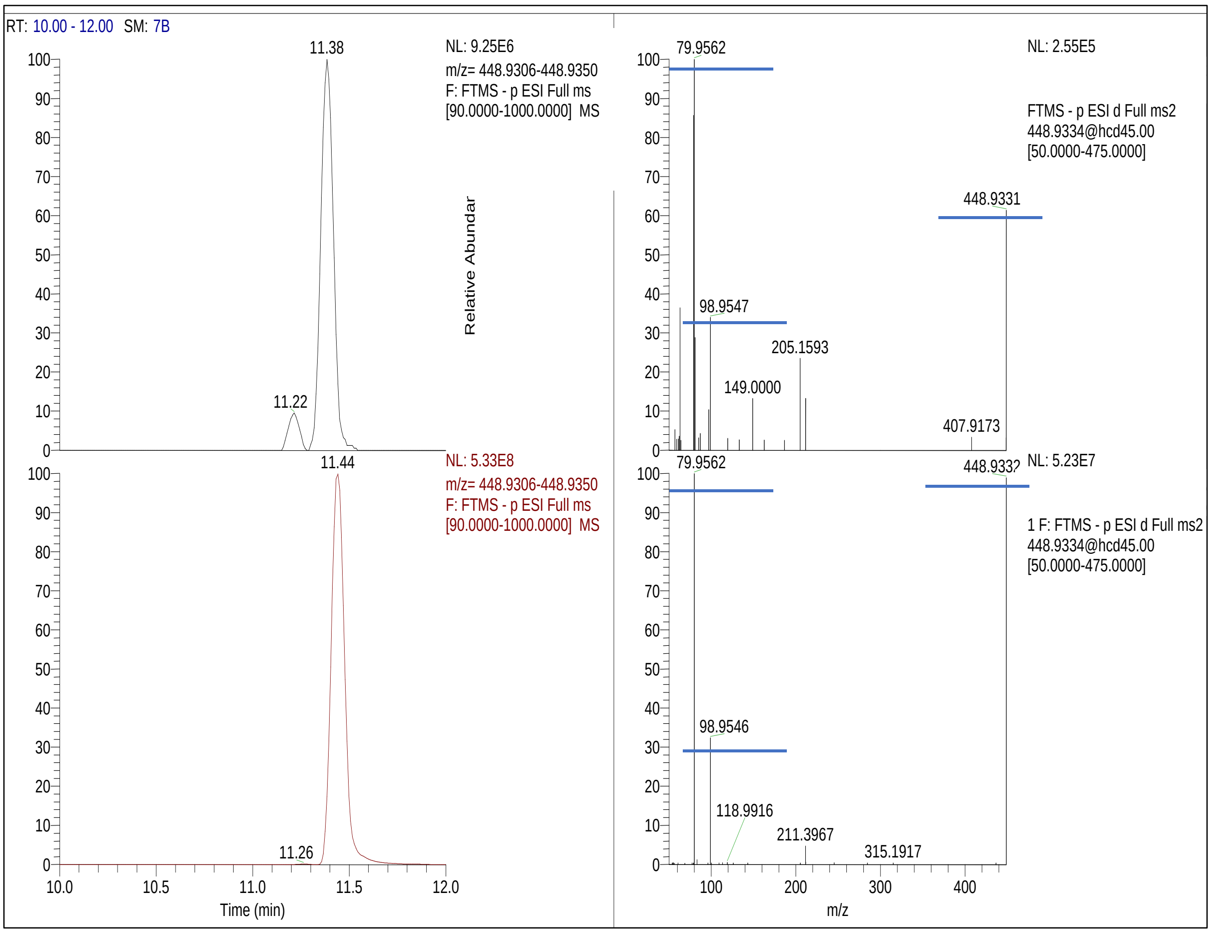

Figure S29. Extracted ion chromatogram (left) and data dependent acquisition (DDA) spectrum (right) for PFHpS in pooled Swedish plasma (top) and spiked pooled plasma (bottom). MS2 ions specific to the precursor are underlined in blue (source: https://mona.fiehnlab.ucdavis.edu/spectra/display/MoNA024593).

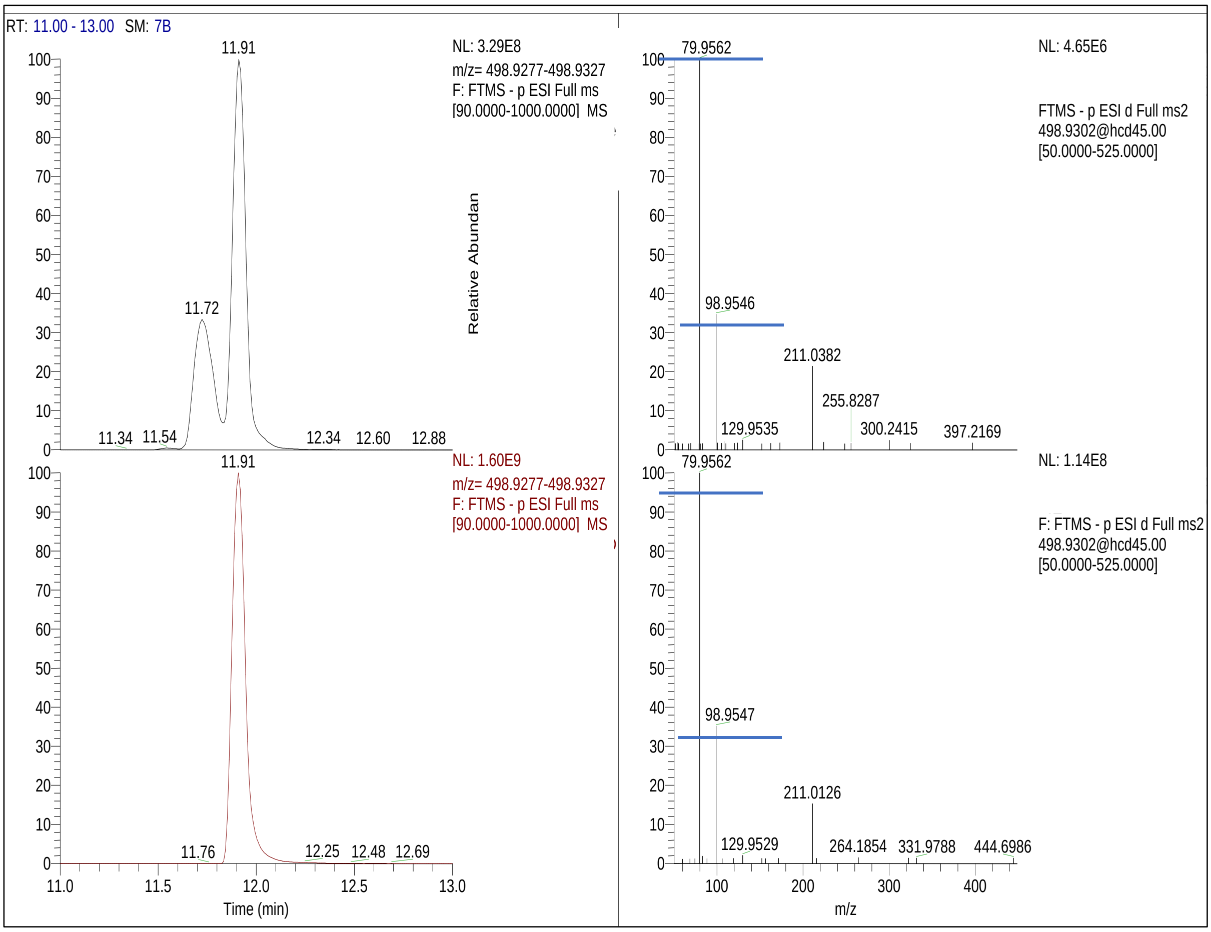

Figure S30. Extracted ion chromatogram (left) and data dependent acquisition (DDA) spectrum (right) for PFOS in pooled Swedish plasma (top) and standard solution (bottom). MS2 ions specific to the precursor are underlined in blue (source: https://massbank.eu/MassBank/RecordDisplay?id=MSBNK-PFOS_research_group-FFF00315&dsn=PFOS_research_group).

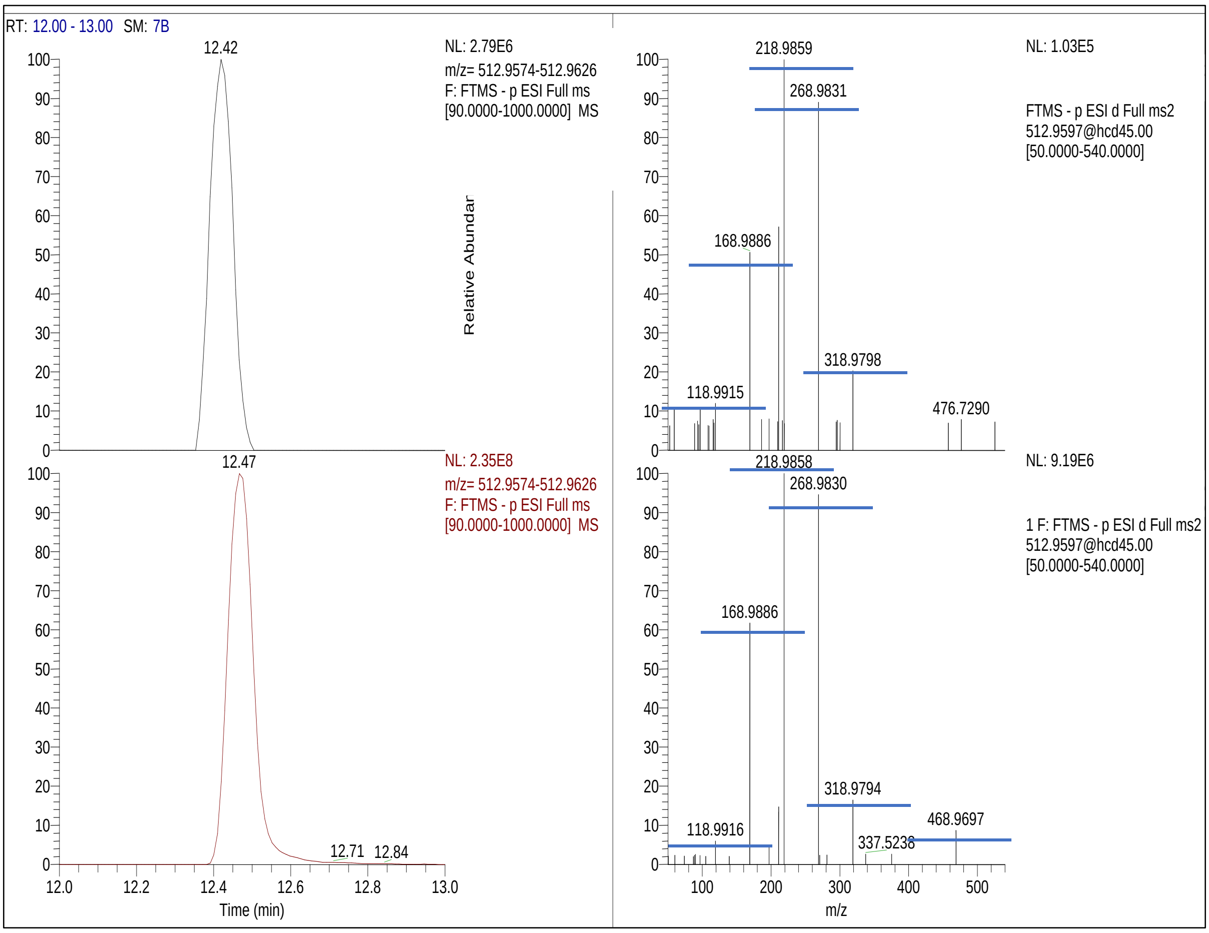

Figure S31. Extracted ion chromatogram (left) and data dependent acquisition (DDA) spectrum (right) for PFDA in pooled Swedish plasma (top) and spiked pooled plasma (bottom). MS2 ions specific to the precursor are underlined in blue (source: https://massbank.eu/MassBank/RecordDisplay?id=MSBNK-Athens_Univ-AU240358&dsn=Athens_Univ).

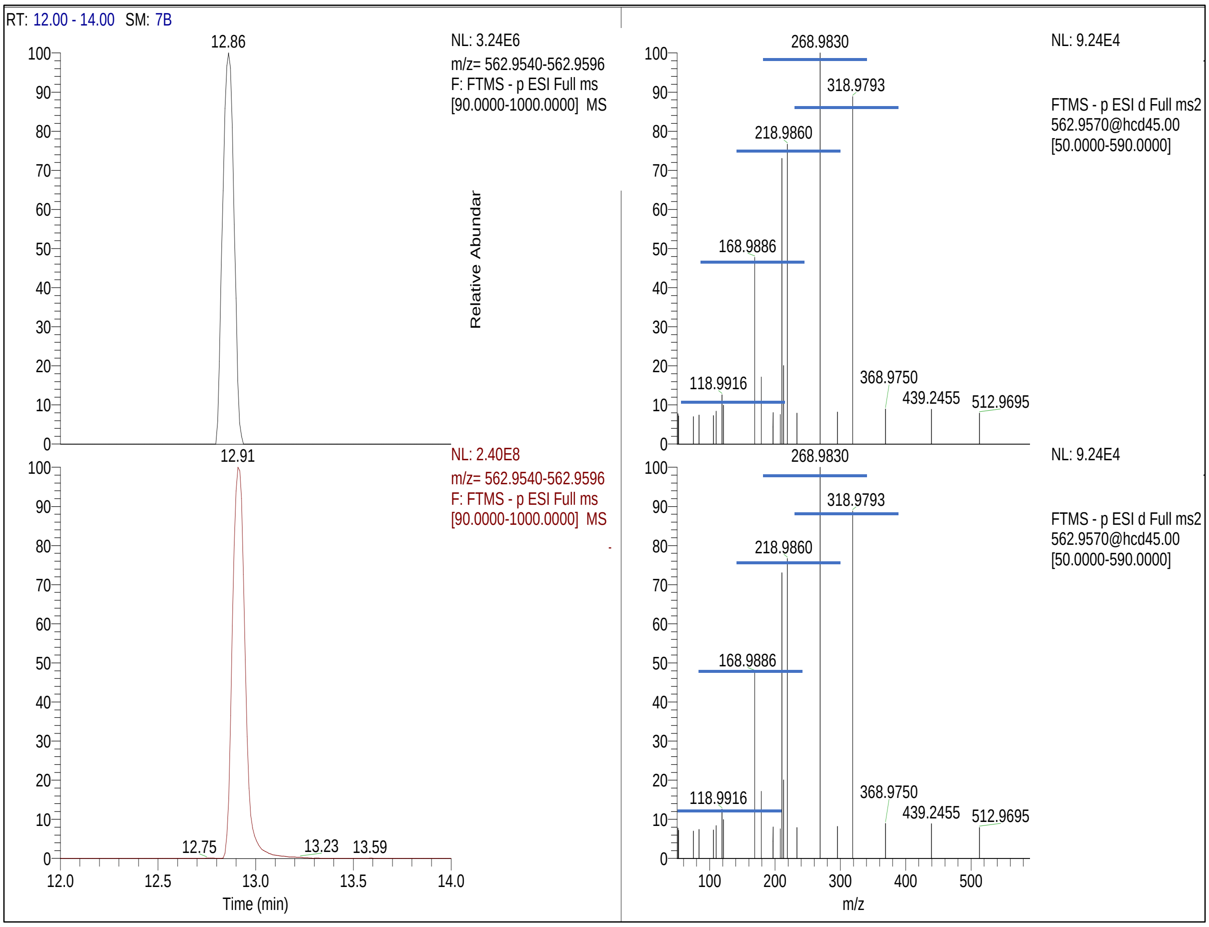

Figure S32. Extracted ion chromatogram (left) and data dependent acquisition (DDA) spectrum (right) for PFUnDA in pooled Swedish plasma (top) and spiked pooled plasma (bottom). MS2 ions specific to the precursor are underlined in blue (source: https://massbank.eu/MassBank/RecordDisplay?id=MSBNK-Athens_Univ-AU595151&dsn=Athens_Univ).

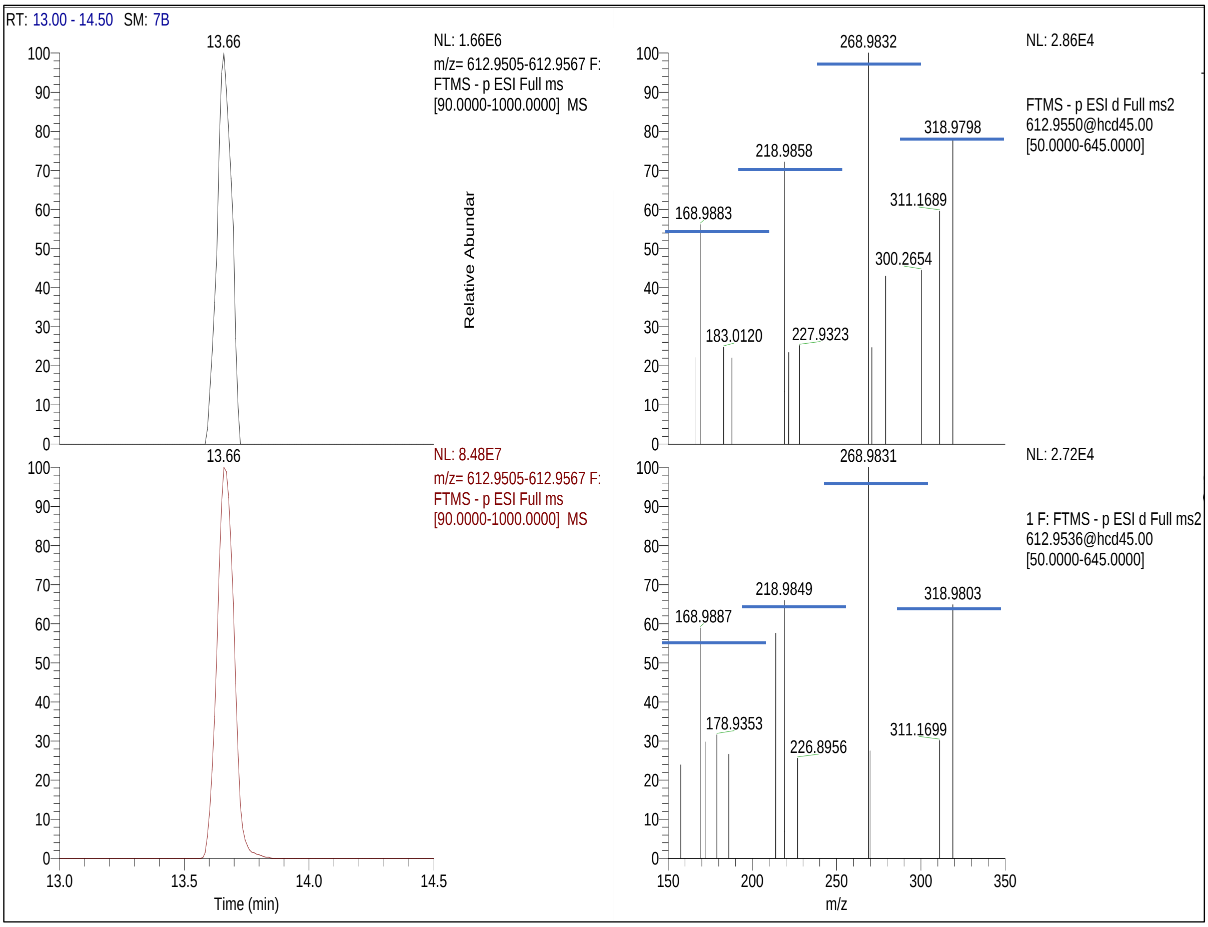

Figure S33. Extracted ion chromatogram (left) and data dependent acquisition (DDA) spectrum (right) for PFDoDA in Individual Swedish plasma (top) and spiked pooled plasma (bottom). MS2 ions specific to the precursor are underlined in blue (source: https://massbank.eu/MassBank/RecordDisplay?id=MSBNK-Athens_Univ-AU595251&dsn=Athens_Univ).

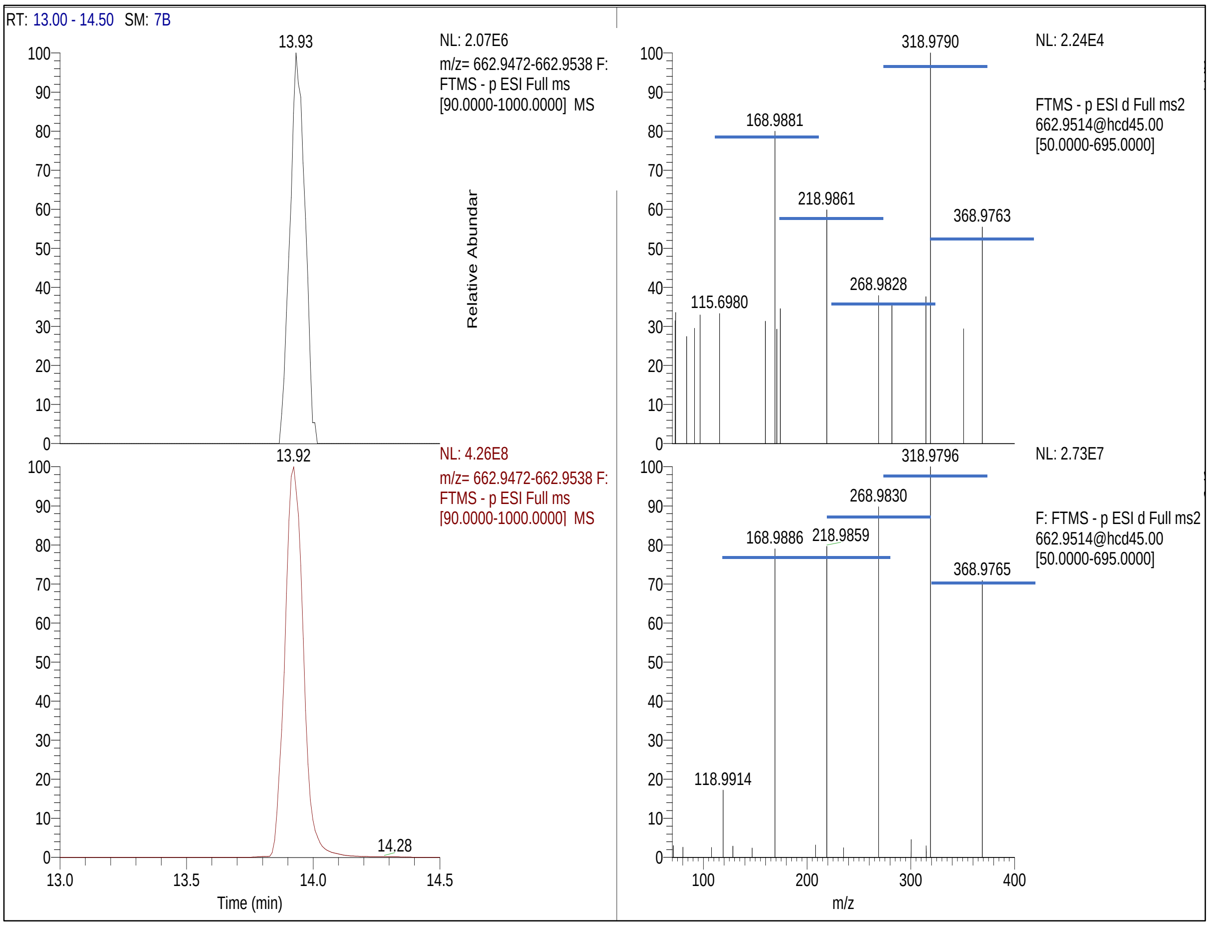

Figure S34. Extracted ion chromatogram (left) and data dependent acquisition (DDA) spectrum (right) for PFTrDA in Individual Swedish plasma (top) and standard solution (bottom). MS2 ions specific to the precursor are underlined in blue (source: https://massbank.eu/MassBank/RecordDisplay?id=MSBNK-Athens_Univ-AU595351&dsn=Athens_Univ).

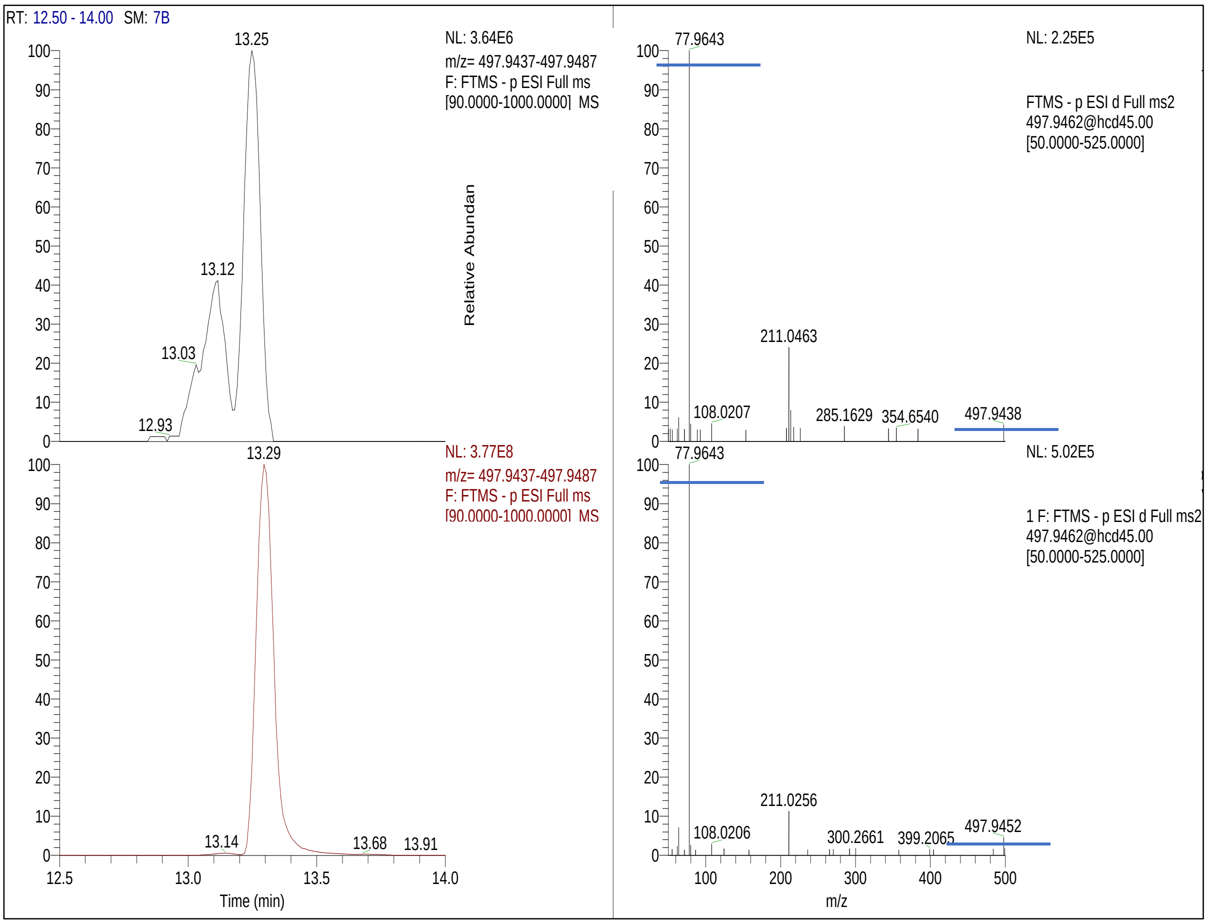

Figure S35. Extracted ion chromatogram (left) and data dependent acquisition (DDA) spectrum (right) for FOSA in pooled Swedish plasma (top) and spiked pooled plasma (bottom). MS2 ions specific to the precursor are underlined in blue (source: https://massbank.eu/MassBank/RecordDisplay?id=MSBNK-LCSB-LU056351&dsn=LCSB).

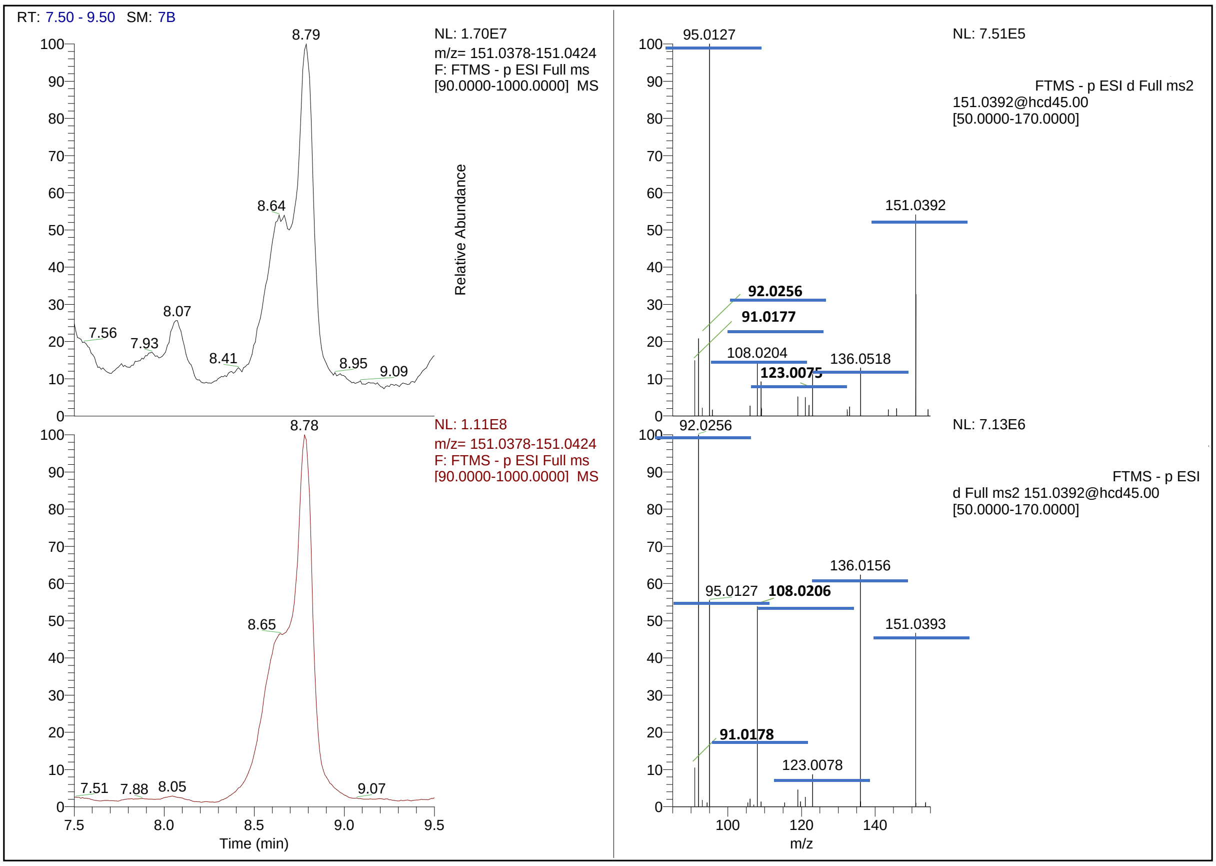

Figure S36. Extracted ion chromatogram (left) and data dependent acquisition (DDA) spectrum (right) for methylparaben in pooled Swedish plasma (top) and spiked pooled plasma (bottom). MS2 ions specific to the precursor are underlined in blue (source: https://massbank.eu/MassBank/RecordDisplay?id=MSBNK-Athens_Univ-AU237162&dsn=Athens_Univ).

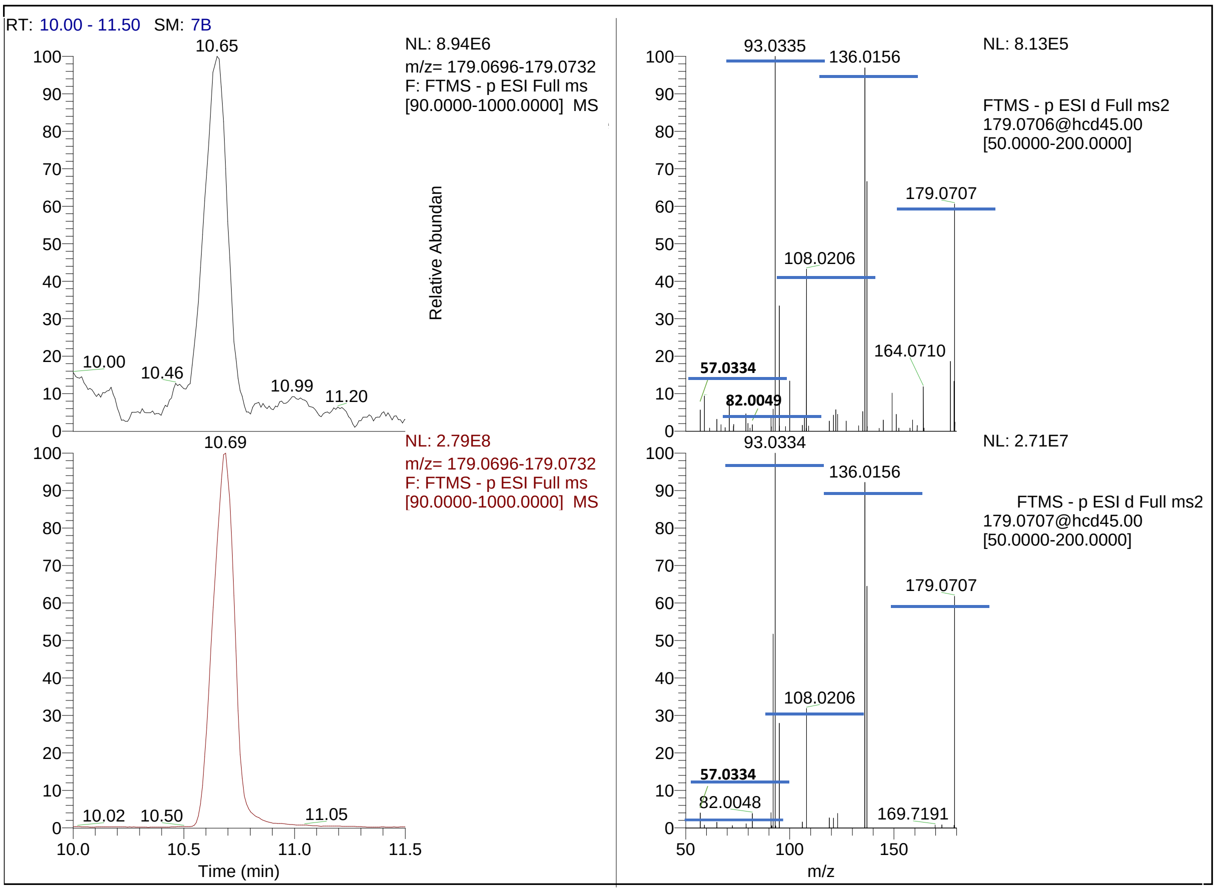

Figure S37. Extracted ion chromatogram (left) and data dependent acquisition (DDA) spectrum (right) for propyl paraben in pooled Swedish plasma (top) and spiked pooled plasma (bottom). MS2 ions specific to the precursor are underlined in blue (source: https://massbank.eu/MassBank/RecordDisplay?id=MSBNK-Athens_Univ-AU237262&dsn=Athens_Univ).

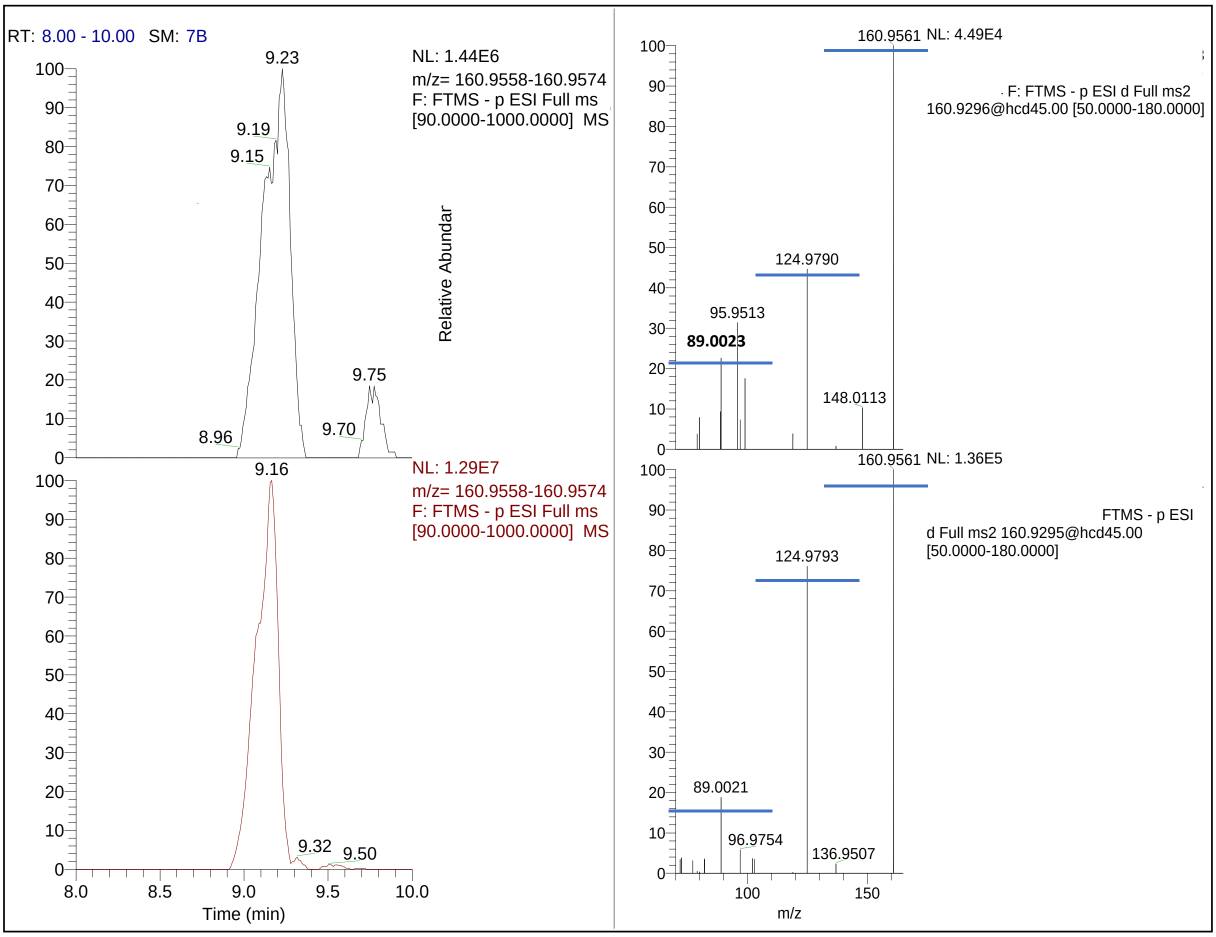

Figure S38. Extracted ion chromatogram (left) and data dependent acquisition (DDA) spectrum (right) for 2,5 dichlorophenol in Individual Swedish plasma (top) and spiked pooled plasma (bottom). MS2 ions specific to the precursor are underlined in blue (source: https://massbank.eu/MassBank/RecordDisplay?id=MSBNK-LCSB-LU090654&dsn=LCSB).

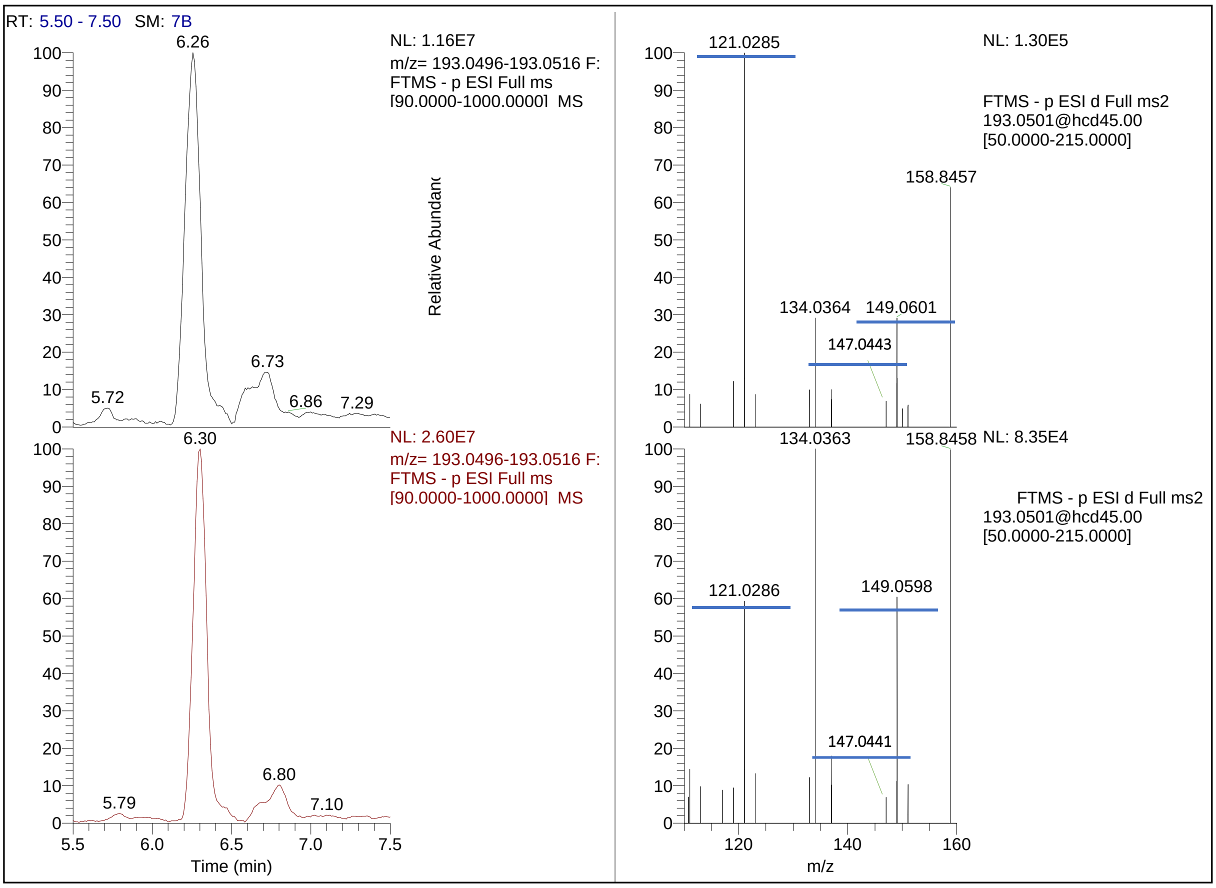

Figure S39. Extracted ion chromatogram (left) and data dependent acquisition (DDA) spectrum (right) for monoethyl phthalate in pooled Swedish plasma (top) and spiked pooled plasma (bottom). MS2 ions specific to the precursor are underlined in blue (source: https://massbank.eu/MassBank/RecordDisplay?id=MSBNK-CASMI_2016-SM823251&dsn=CASMI_2016).

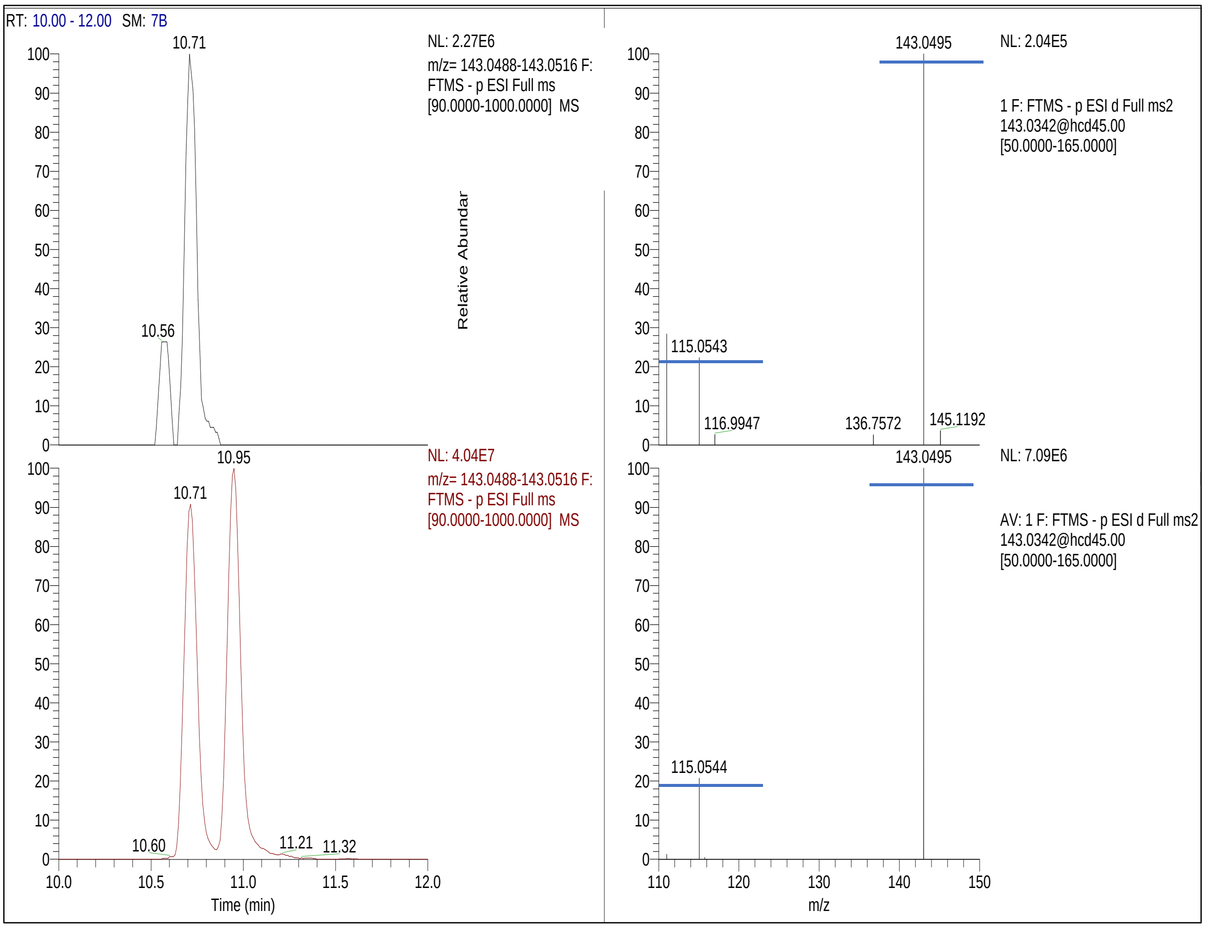

Figure S40. Extracted ion chromatogram (left) and data dependent acquisition (DDA) spectrum (right) for 2-naphthol in pooled Swedish plasma (top) and spiked pooled plasma (bottom). In the spiked pooled plasma 2- naphthol elutes at 10.71 min and 1- naphthol at 10.95 min. MS2 ions specific to the precursor are underlined in blue (source: Jacob et al.[10]).

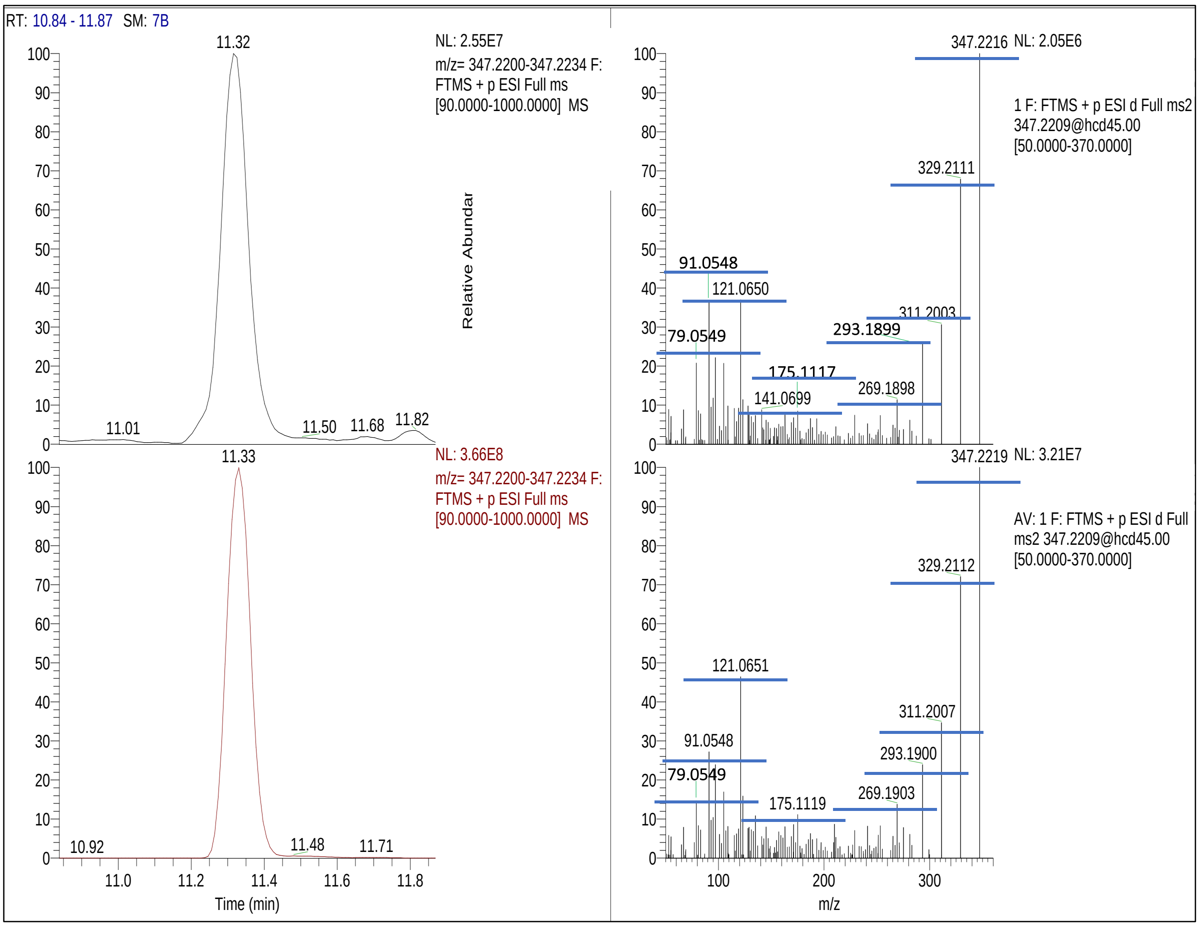

Figure S41. Extracted ion chromatogram (left) and data dependent acquisition (DDA) spectrum (right) for corticosterone in pooled Swedish plasma (top) and standard solution (bottom). MS2 ions specific to the precursor are underlined in blue (source: https://massbank.eu/MassBank/RecordDisplay?id=MSBNK-Eawag-EQ319803&dsn=Eawag).

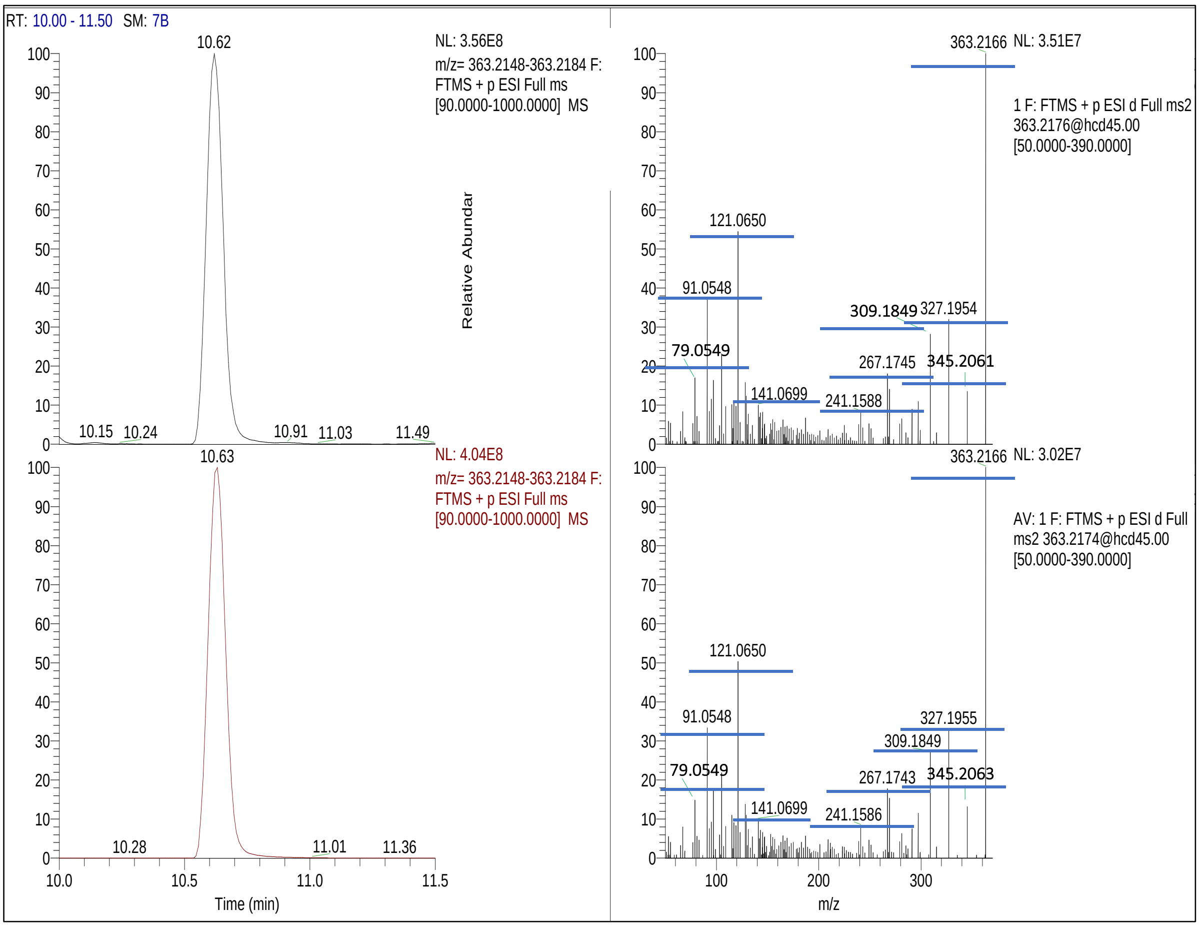

Figure S42. Extracted ion chromatogram (left) and data dependent acquisition (DDA) spectrum (right) for hydrocortisone in pooled Swedish plasma (top) and standard solution (bottom). MS2 ions specific to the precursor are underlined in blue (source: https://massbank.eu/MassBank/RecordDisplay?id=MSBNK-Univ_Connecticut-CO000223&dsn=Univ_Connecticut).

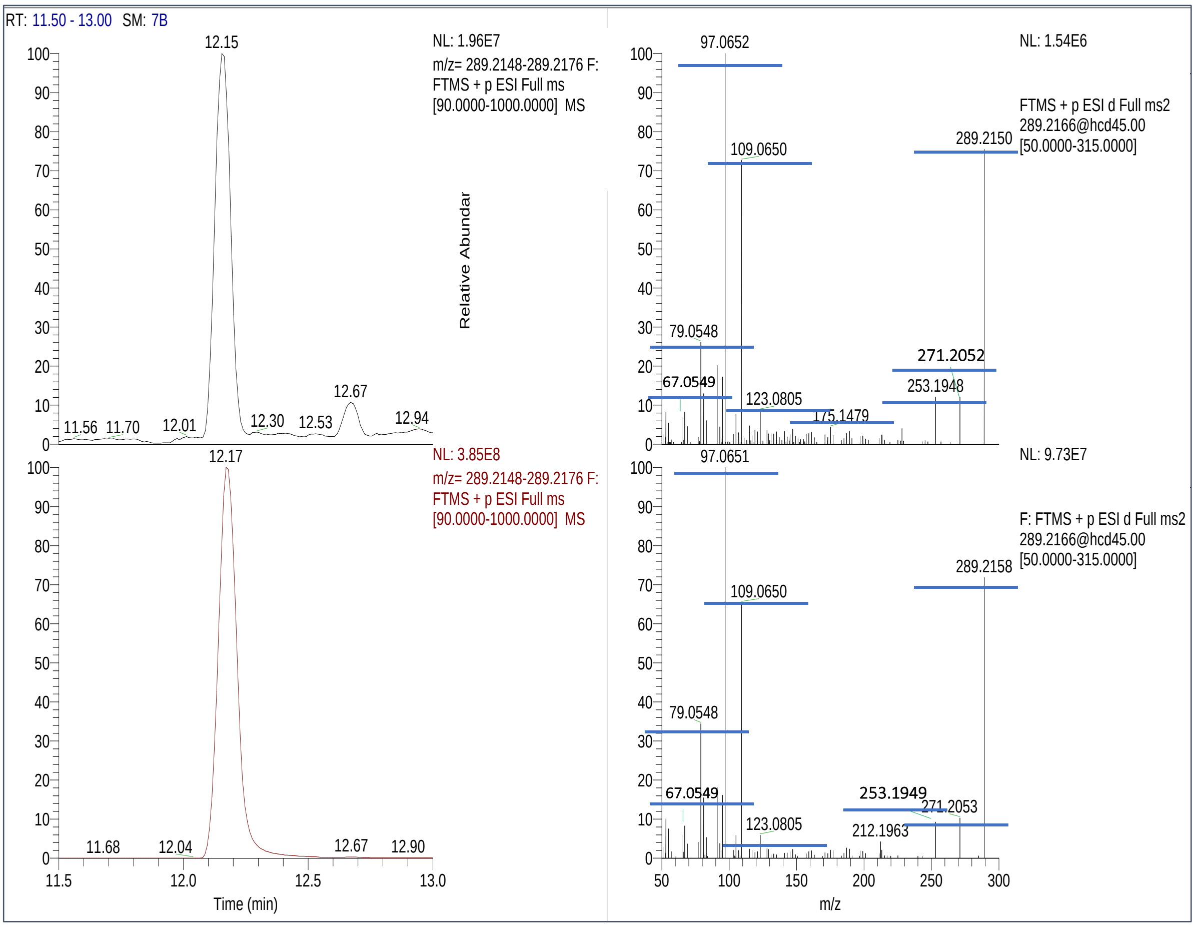

Figure S43. Extracted ion chromatogram (left) and data dependent acquisition (DDA) spectrum (right) for testosterone in pooled Swedish plasma (top) and standard solution (bottom). MS2 ions specific to the precursor are underlined in blue (source: https://massbank.eu/MassBank/RecordDisplay?id=MSBNK-Athens_Univ-AU280206&dsn=Athens_Univ).

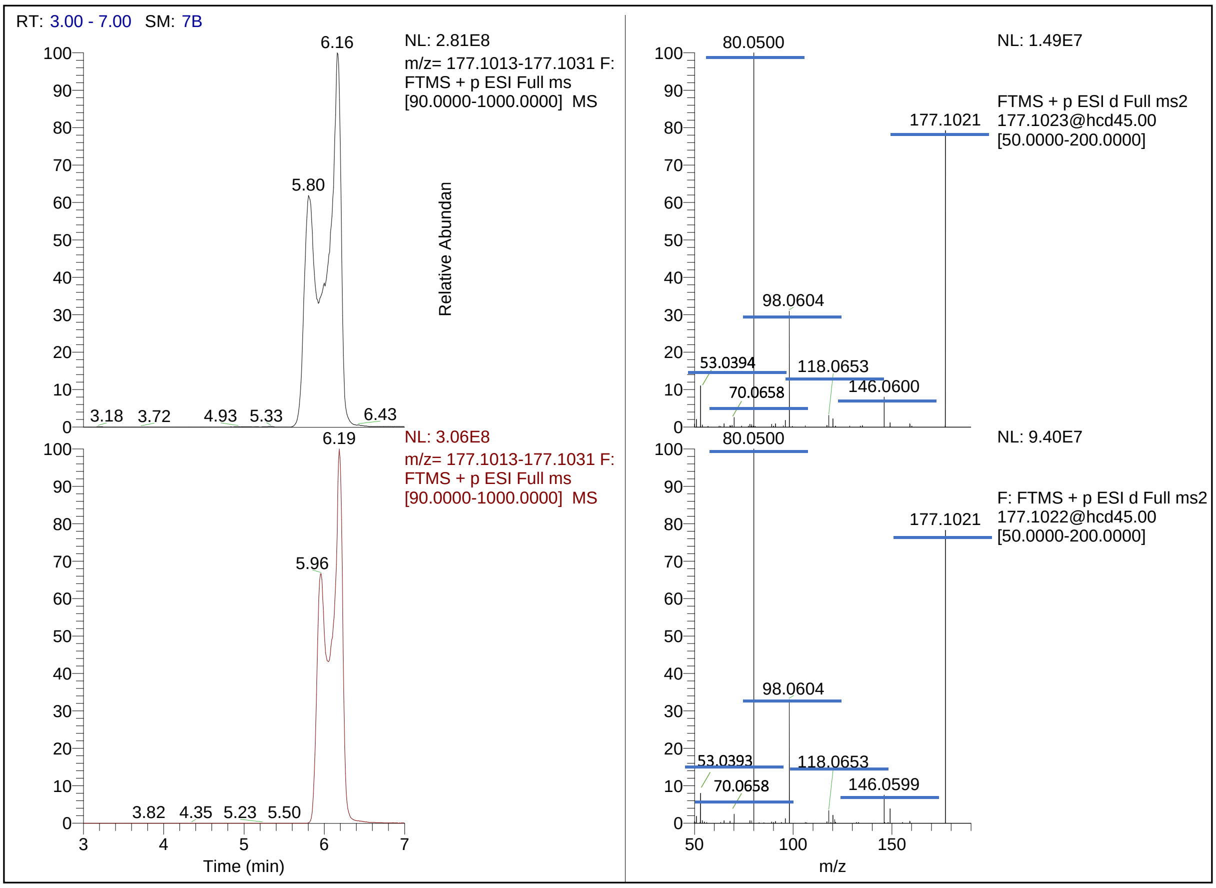

Figure S44. Extracted ion chromatogram (left) and data dependent acquisition (DDA) spectrum (right) for cotinine in pooled Swedish plasma (top) and standard solution (bottom). MS2 ions specific to the precursor are underlined in blue (source: https://massbank.eu/MassBank/RecordDisplay?id=MSBNK-Eawag-EQ328205&dsn=Eawag).

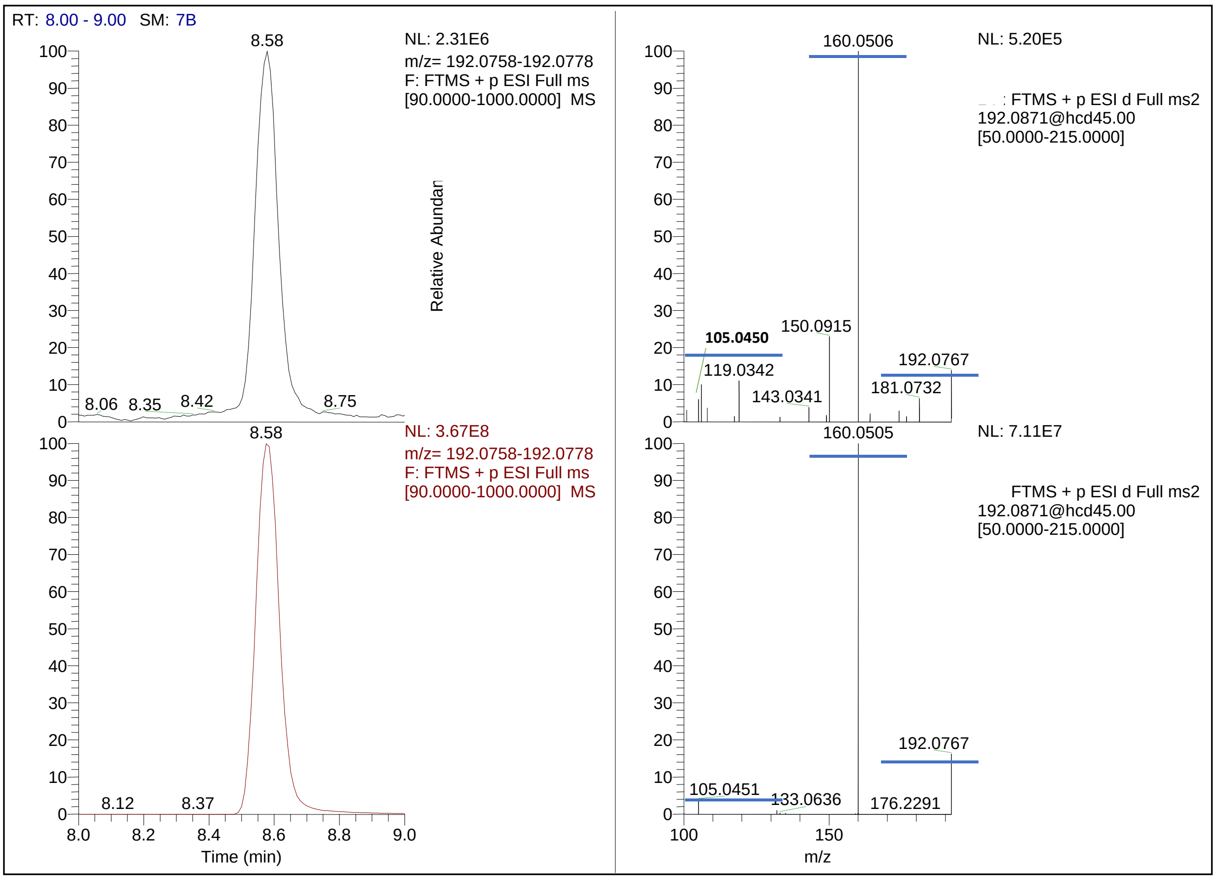

Figure S45. Extracted ion chromatogram (left) and data dependent acquisition (DDA) spectrum (right) for carbendazim in Individual Swedish plasma (top) and spiked pooled plasma (bottom). MS2 ions specific to the precursor are underlined in blue (source: https://mona.fiehnlab.ucdavis.edu/spectra/display/CCMSLIB00001058284).

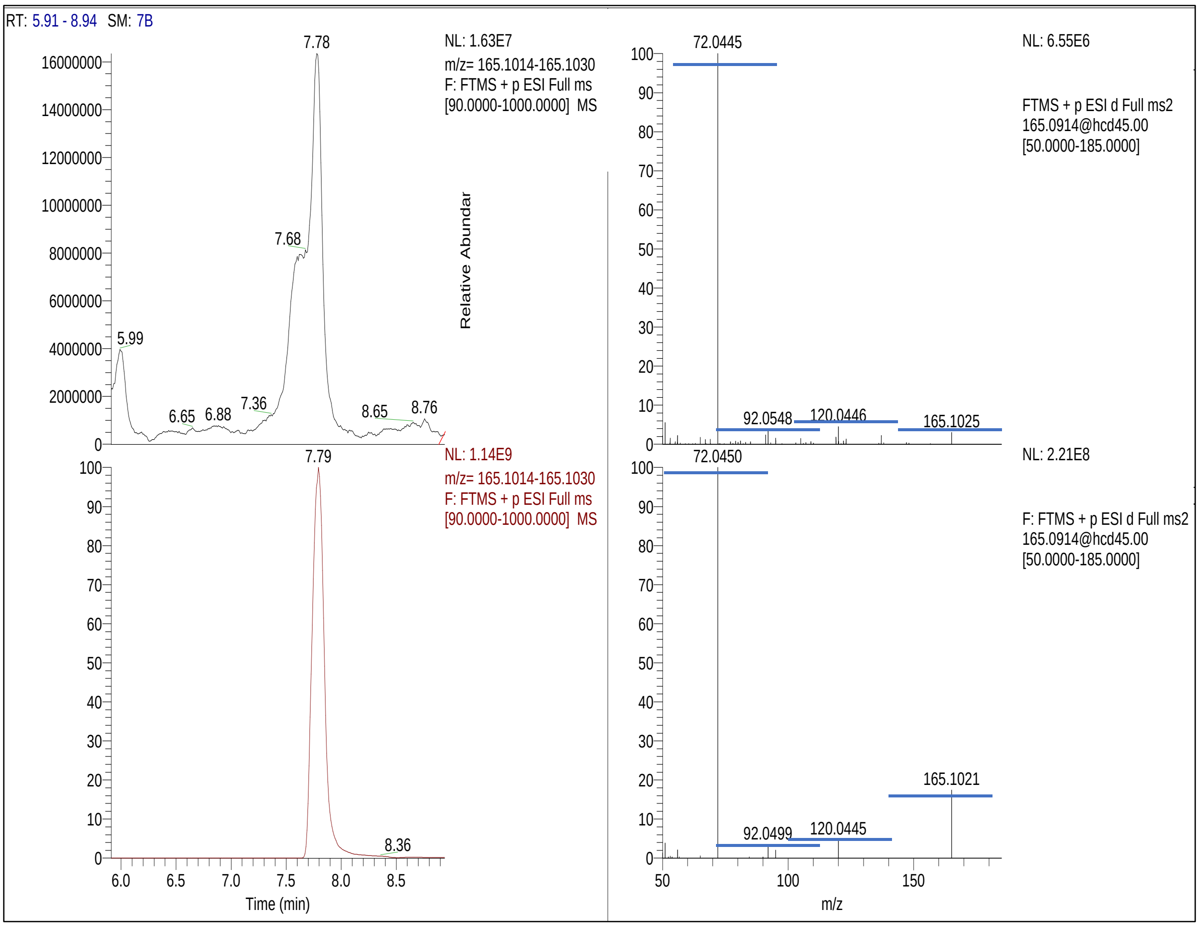

Figure S46. Extracted ion chromatogram (left) and data dependent acquisition (DDA) spectrum (right) for fenuron in Individual Swedish plasma (top) and standard solution (bottom). MS2 ions specific to the precursor are underlined in blue (source: Wang et al.[11]).

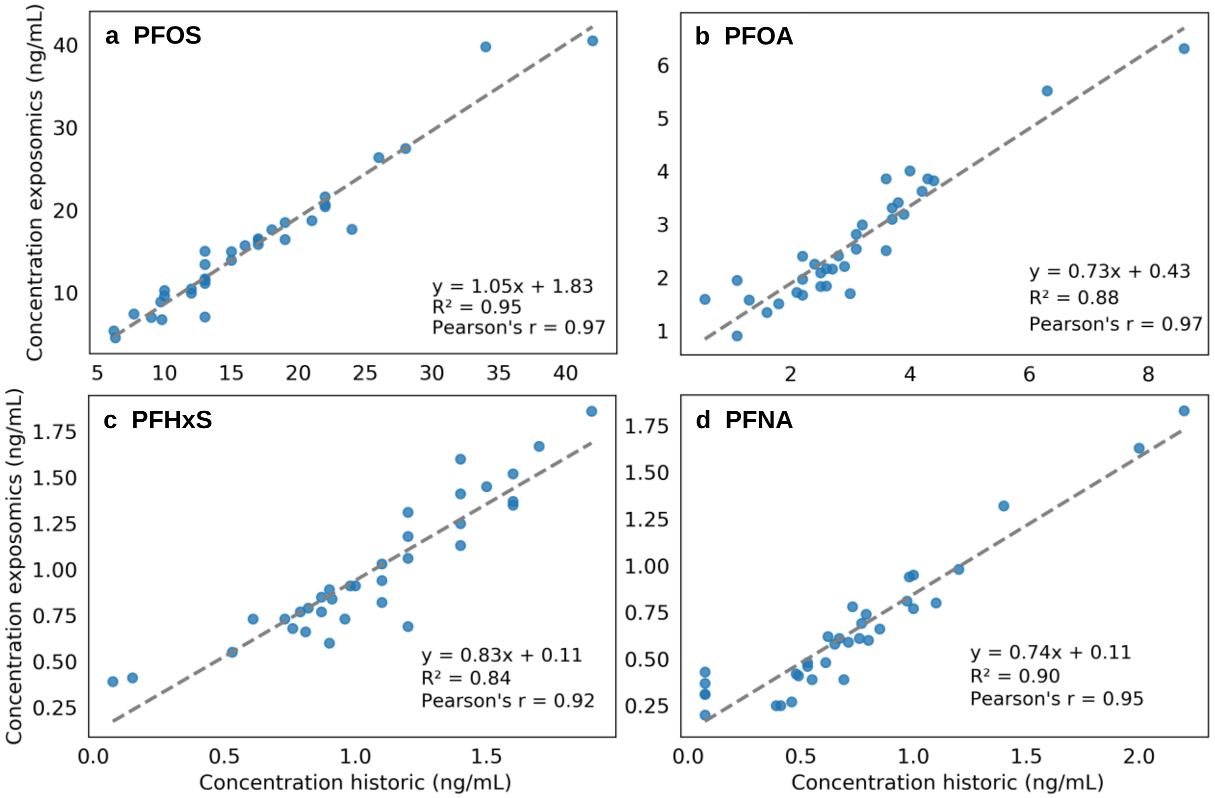

Figure S47. External validation of method accuracy for the quantification of 4 PFAS by the exposomics protocol. Linear regression is shown for (a) PFOS, (b) PFOA, (c) PFHxS and (d) PFNA concentrations quantified by the exposomics protocol (y-axis) and by a targeted PFAS method in a former study (x-axis).[12] For each regression line, the equation, R-squared value (R^2) and Pearson correlation coefficient are reported.

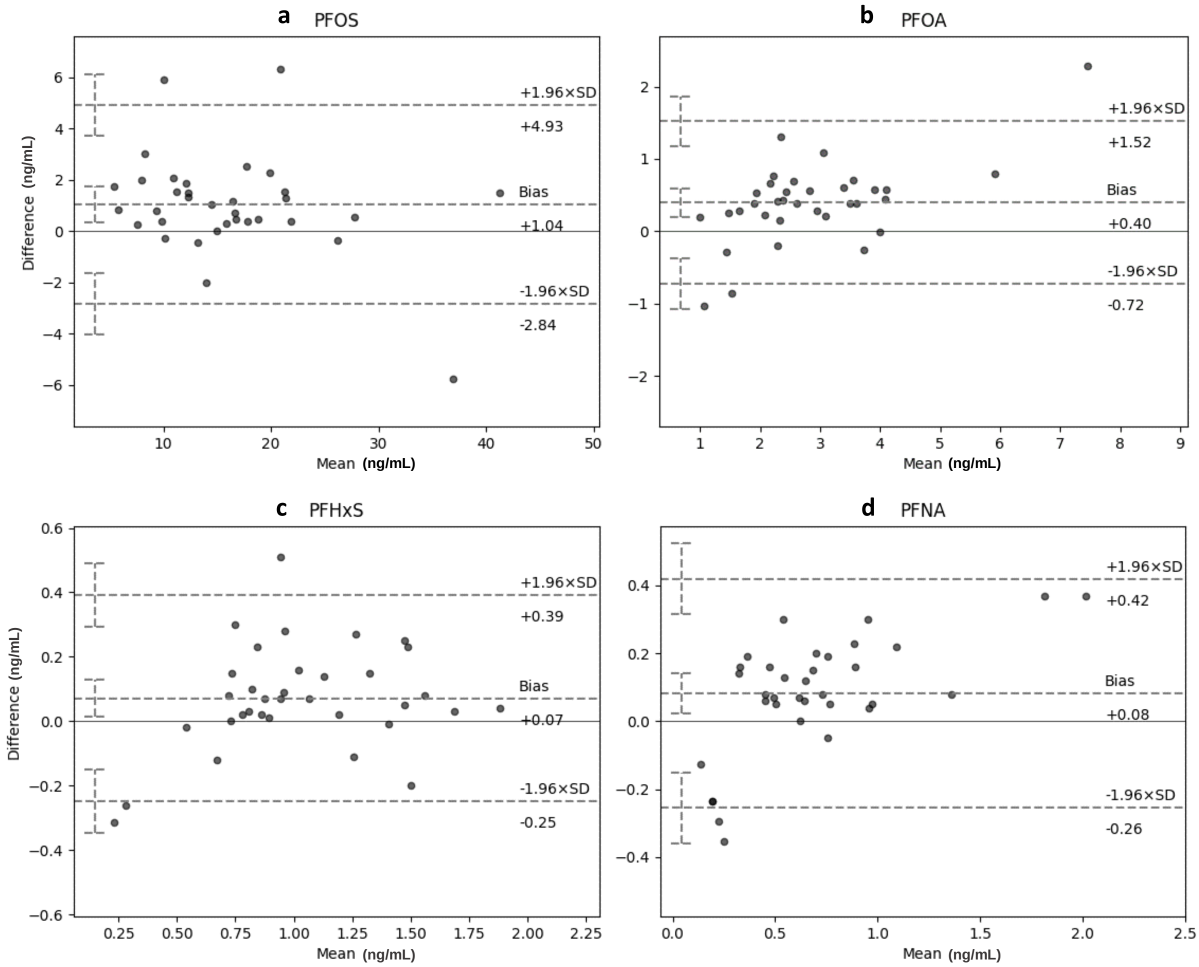

Figure S48. Bland-Altman Plots showing paired differences between concentrations obtained by a targeted PFAS method [12] and the current exposomics method versus the mean of the two methods. Horizontal lines are drawn at the 0 difference (solid line), mean of differences or bias (dashed middle line) and at the limits of agreement (dashed outer lines). Vertical bars present confidence interval limits for bias and agreement limits. Plots are shown for (a) PFOS, (b) PFOA, (c) PFHxS and (d) PFNA. Greatest differences (i.e. < -0.25 for PFHxS, and < -0.1 for PFNA), at concentrations < 0.5 ng/mL were only observed when the targeted analysis reported values below the quantification limits, which were substituted by LOQ/2.

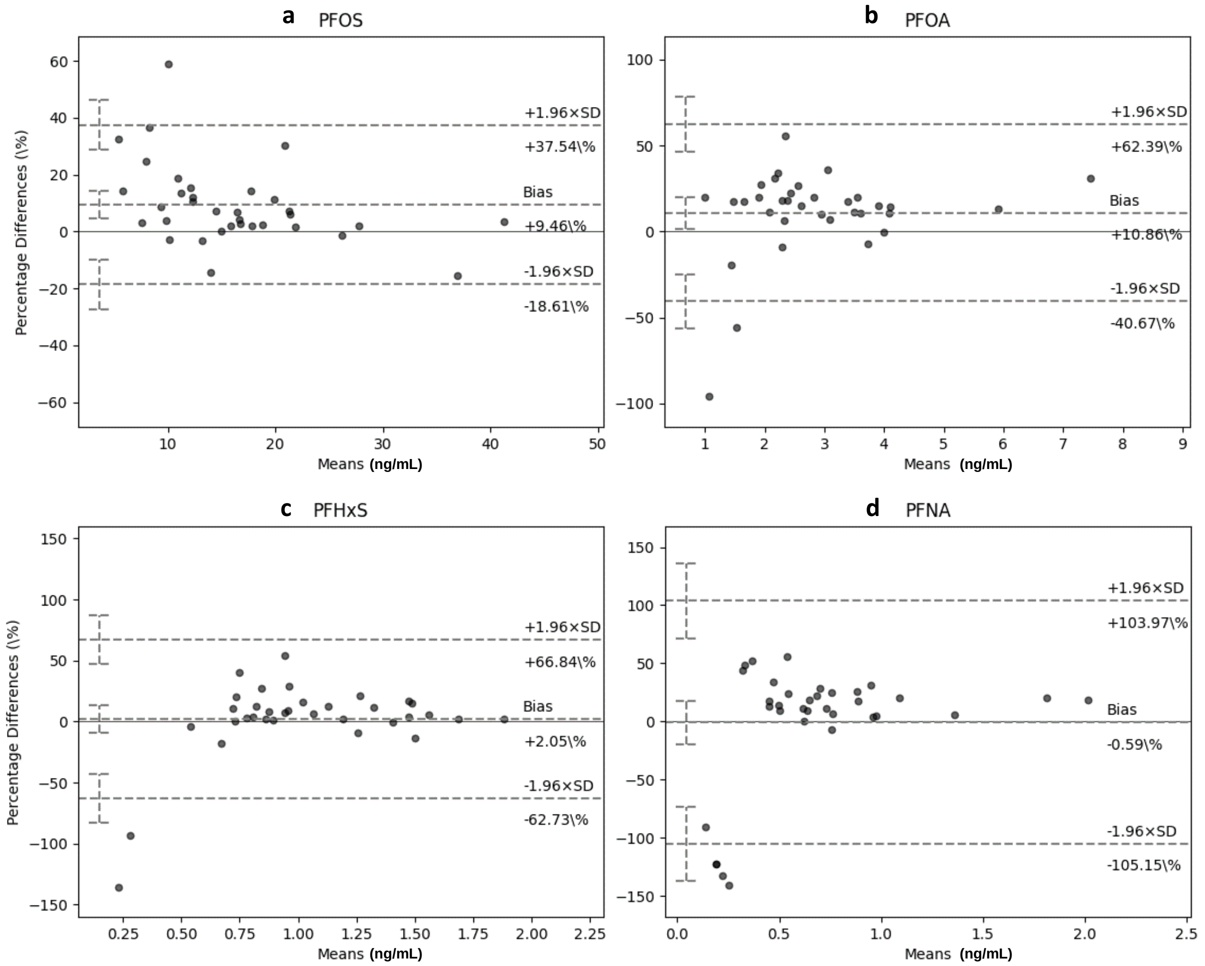

Figure S49. Bland-Altman Plots showing the paired percentage differences between concentrations obtained by a targeted PFAS method [12] and by the current exposomics method versus the mean of the two methods. Differences are expressed as percentages calculated as (concentrations historic – concentrations exposomics)/mean%. Horizontal lines are drawn at the 0 difference (solid line), mean of differences or bias (dashed middle line) and at the limits of agreement (dashed outer lines). Vertical bars present confidence interval limits for bias and agreement limits. Plots are shown for (a) PFOS, (b) PFOA, (c) PFHxS and (d) PFNA. Greatest percentage differences for PFHxS and PFNA (i.e. < -50%), at concentrations < 0.5 ng/mL were only observed when the targeted analysis reported values below the quantification limits, which were substituted by LOQ/2.

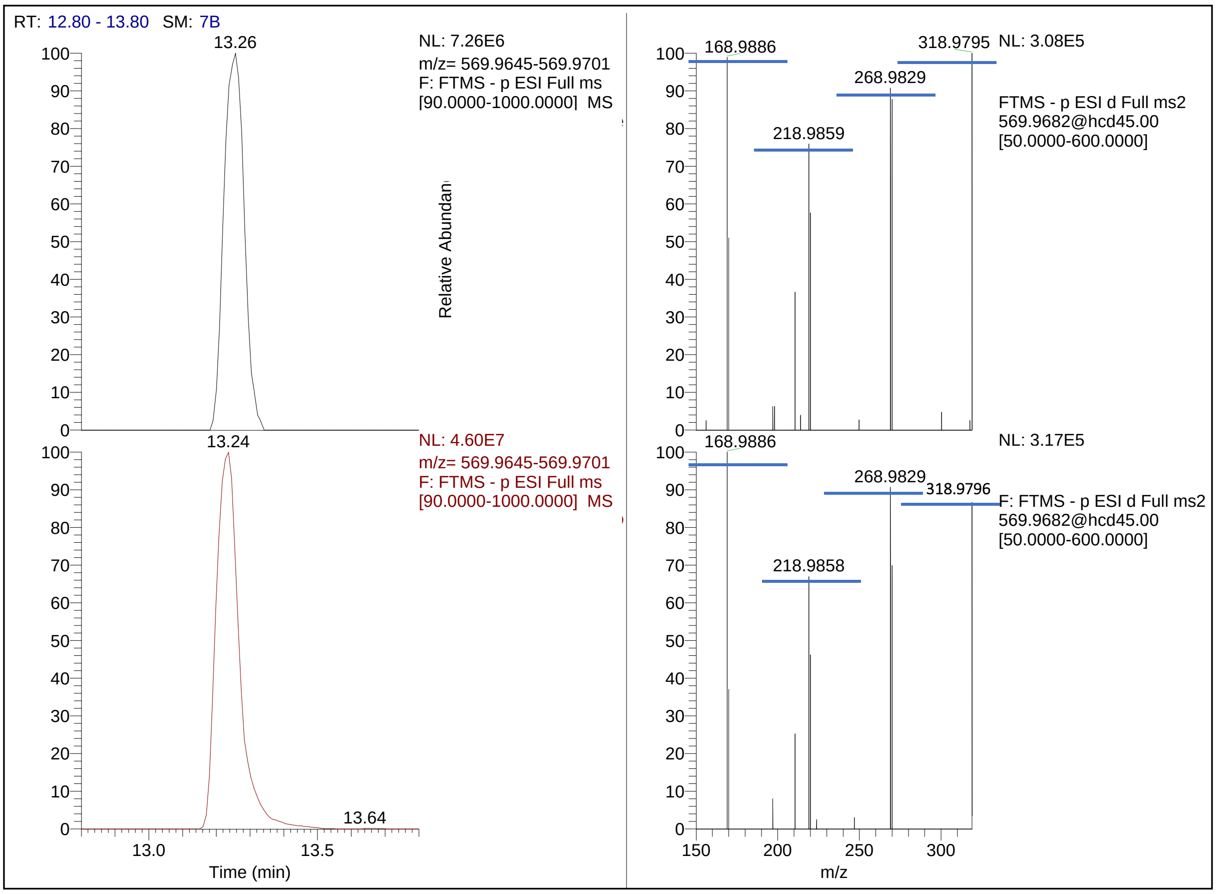

Figure S50. Extracted ion chromatogram (left) and data dependent acquisition (DDA) spectrum (right) for NMeFOSA in pooled Swedish plasma (top) and standard solution (bottom). MS2 ions specific to the precursor are underlined in blue (source: Gebbink et al.[13]).

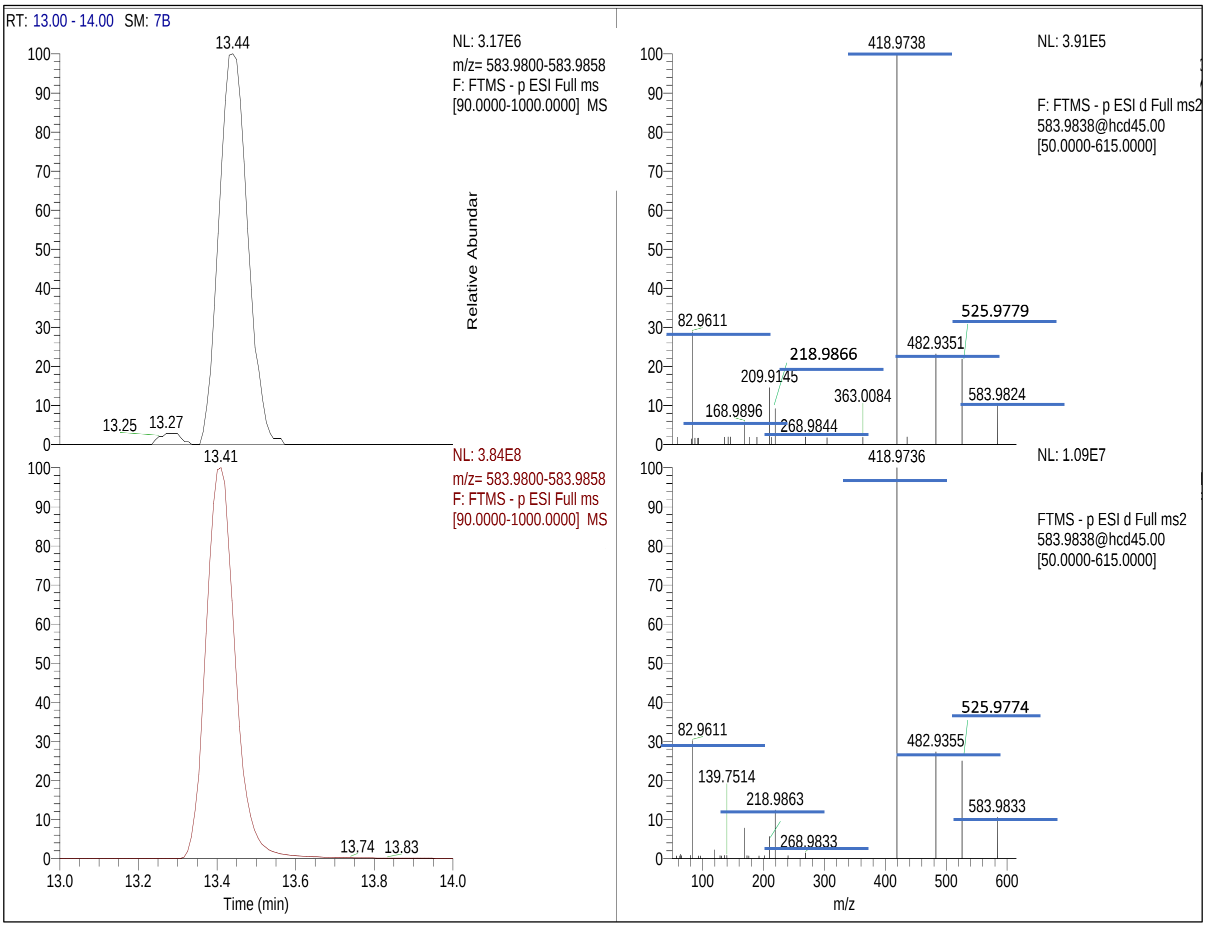

Figure S51. Extracted ion chromatogram (left) and data dependent acquisition (DDA) spectrum (right) for NEtFOSA in pooled Swedish plasma (top) and standard solution (bottom). MS2 ions specific to the precursor are underlined in blue (source: Bugsel et al.[14]).

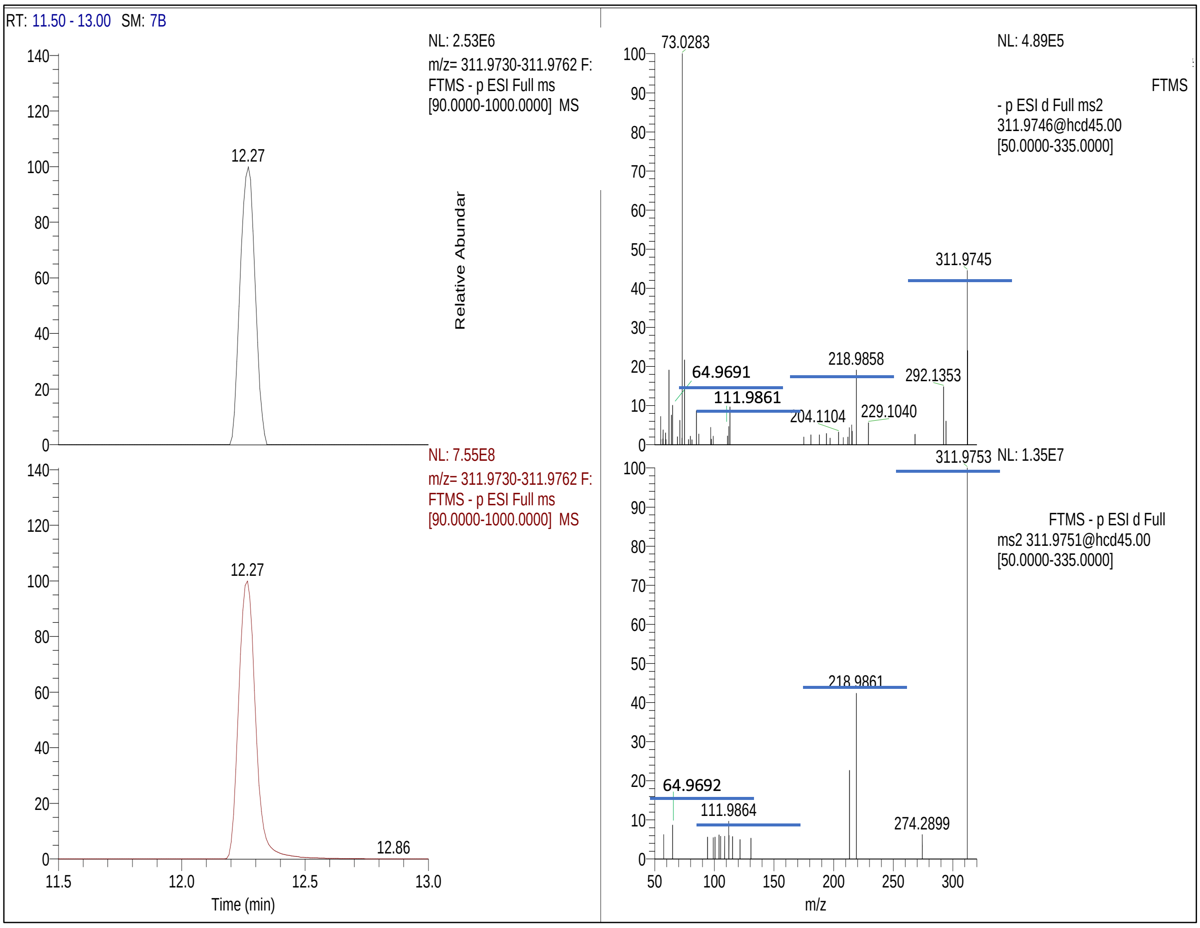

Figure S52. Extracted ion chromatogram (left) and data dependent acquisition (DDA) spectrum (right) for MeFBSA in pooled Swedish plasma (top) and standard solution (bottom). MS2 ions specific to the precursor are underlined in blue (source: Gebbink et al.[13] and Koelmel et al.[15]).

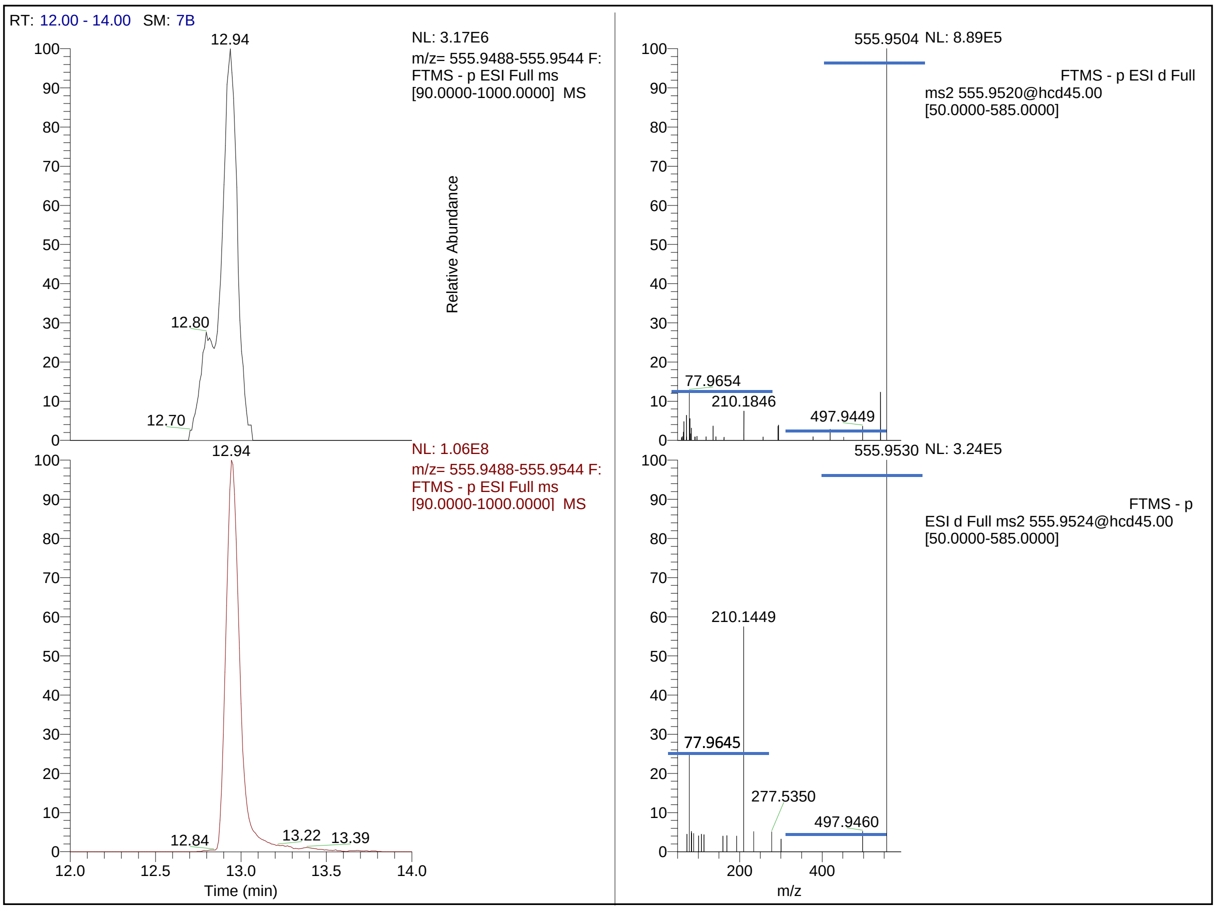

Figure S53. Extracted ion chromatogram (left) and data dependent acquisition (DDA) spectrum (right) for FOSAA in pooled Swedish plasma (top) and spiked pooled plasma (bottom). MS2 ions specific to the precursor are underlined in blue (source: https://massbank.eu/MassBank/RecordDisplay?id=MSBNK-Eawag-EA291961&dsn=Eawag and https://massbank.eu/MassBank/RecordDisplay?id=MSBNK-Eawag-EA291951&dsn=Eawag).

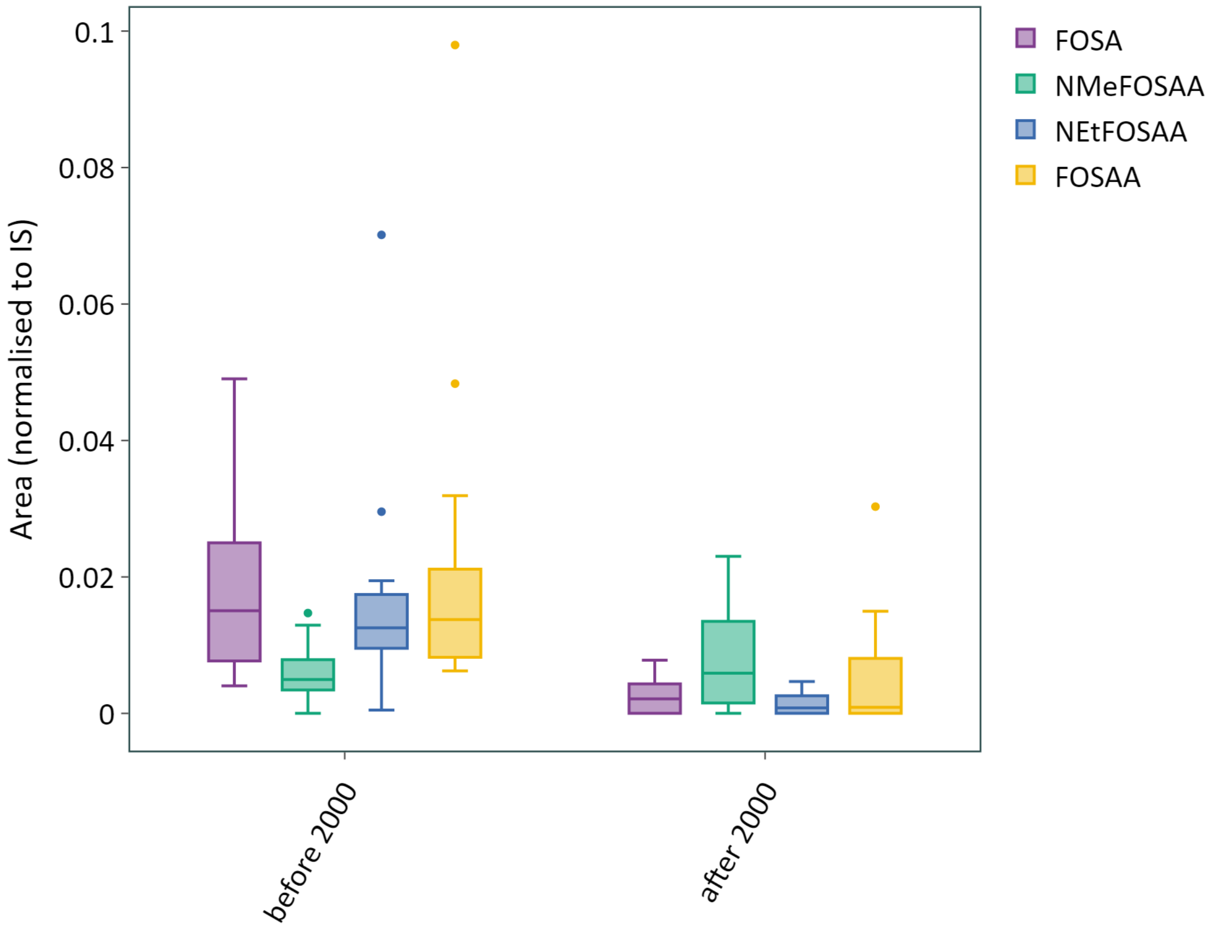

Figure S54. Comparison of exposure to PFOS precursors for individuals sampled before and after the year 2000. PFOS precursor peak areas, normalized to diuron-d6 internal standard (IS) are shown on the left for samples taken before the year 2000 (n = 17), and on the right for samples taken after the year 2000 (n = 17).

References

(1) Ahmad, S.; Kalra, H.; Gupta, A.; Raut, B.; Hussain, A.; Rahman, Md. A. HybridSPE: A Novel Technique to Reduce Phospholipid-Based Matrix Effect in LC–ESI-MS Bioanalysis. *J Pharm Bioallied Sci* **2012**, *4* (4), 267–275. https://doi.org/10.4103/0975-7406.103234.

(2) Anumol, T.; Stevens, J.; Xu, X. Analysis of Per- and Polyfluoroalkyl Substances (PFASs) in Biological Fluid Using a Novel Lipid Removing Sorbent and LC-MS/MS. *Appl. Note Environ. Agil. 8*.

(3) Tsakelidou, E.; Virgiliou, C.; Valianou, L.; Gika, H.; Raikos, N.; Theodoridis, G. Sample Preparation Strategies for the Effective Quantitation of Hydrophilic Metabolites in Serum by Multi-Targeted HILIC-MS/MS. *Metabolites* **2017**, *7* (2), 13. https://doi.org/10.3390/metabo7020013.

(4) Honda, M.; Robinson, M.; Kannan, K. A Rapid Method for the Analysis of Perfluorinated Alkyl Substances in Serum by Hybrid Solid-Phase Extraction. *Environ. Chem.* **2018**, *15* (2), 92. https://doi.org/10.1071/EN17192.

(5) Preindl, K.; Braun, D.; Aichinger, G.; Sieri, S.; Fang, M.; Marko, D.; Warth, B. A Generic Liquid Chromatography−Tandem Mass Spectrometry Exposome Method for the Determination of Xenoestrogens in Biological Matrices. *Anal. Chem.* **2019**, *91* (17), 11334–11342. https://doi.org/10.1021/acs.analchem.9b02446.

(6) Dolan, J. How Much Can I Inject? Part I: Injecting in Mobile Phase. *LCGC North America* **2014**, *32* (10), 780–785.

(7) Sud, M.; Fahy, E.; Cotter, D.; Brown, A.; Dennis, E. A.; Glass, C. K.; Merrill, A. H.; Murphy, R. C.; Raetz, C. R. H.; Russell, D. W.; Subramaniam, S. LMSD: LIPID MAPS Structure Database. *Nucleic Acids Research* **2007**, *35* (Database), D527–D532. https://doi.org/10.1093/nar/gkl838.

(8) Kind, T. *LipidMaps DB 2008*. LipidMaps DB 2008. https://fiehnlab.ucdavis.edu/downloads/staff/kind/Metabolomics/LipidAnalysis/lipidmapsmar2008-small.xls (accessed 2023-03-29).

(9) Jiang, H.; Zhang, Y.; Chen, X.; Lv, J.; Zou, J. Simultaneous Determination of Pentachlorophenol, Niclosamide and Fenpropathrin in Fishpond Water Using an LC-MS/MS Method for Forensic Investigation. *Analytical Methods* **2013**, *5* (1), 111–115. https://doi.org/10.1039/C2AY25685A.

(10) Jacob, P.; Wilson, M.; Benowitz, N. L. Determination of Phenolic Metabolites of Polycyclic Aromatic Hydrocarbons in Human Urine as Their Pentafluorobenzyl Ether Derivatives Using Liquid Chromatography−Tandem Mass Spectrometry. *Anal. Chem.* **2007**, *79* (2), 587–598. https://doi.org/10.1021/ac060920l.

(11) Wang, Q.-Y.; Yang, J.; Dong, X.; Chen, Y.; Ye, L.-H.; Hu, Y.-H.; Zheng, H.; Cao, J. Zirconium Metal-Organic Framework Assisted Miniaturized Solid Phase Extraction of Phenylurea Herbicides in Natural Products by Ultra-High-Performance Liquid Chromatography Coupled with Quadrupole Time-of-Flight Mass Spectrometry. *Journal of Pharmaceutical and Biomedical Analysis* **2020**, *180*, 113071. https://doi.org/10.1016/j.jpba.2019.113071.

(12) Donat-Vargas, C.; Bergdahl, I. A.; Tornevi, A.; Wennberg, M.; Sommar, J.; Kiviranta, H.; Koponen, J.; Rolandsson, O.; Åkesson, A. Perfluoroalkyl Substances and Risk of Type II Diabetes: A Prospective Nested Case-Control Study. *Environment International* **2019**, *123*, 390–398. https://doi.org/10.1016/j.envint.2018.12.026.

(13) Gebbink, W. A.; Glynn, A.; Darnerud, P. O.; Berger, U. Perfluoroalkyl Acids and Their Precursors in Swedish Food: The Relative Importance of Direct and Indirect Dietary Exposure. *Environmental Pollution* **2015**, *198*, 108–115. https://doi.org/10.1016/j.envpol.2014.12.022.

(14) Bugsel, B.; Bauer, R.; Herrmann, F.; Maier, M. E.; Zwiener, C. LC-HRMS Screening of per- and Polyfluorinated Alkyl Substances (PFAS) in Impregnated Paper Samples and Contaminated Soils. *Anal Bioanal Chem* **2022**, *414* (3), 1217–1225. https://doi.org/10.1007/s00216-021-03463-9.

(15) Koelmel, J. P.; Paige, M. K.; Aristizabal-Henao, J. J.; Robey, N. M.; Nason, S. L.; Stelben, P. J.; Li, Y.; Kroeger, N. M.; Napolitano, M. P.; Savvaides, T.; Vasiliou, V.; Rostkowski, P.; Garrett, T. J.; Lin, E.; Deigl, C.; Jobst, K.; Townsend, T. G.; Godri Pollitt, K. J.; Bowden, J. A. Toward Comprehensive Per- and Polyfluoroalkyl Substances Annotation Using FluoroMatch Software

and Intelligent High-Resolution Tandem Mass Spectrometry Acquisition. *Anal. Chem.* **2020**, *92* (16), 11186–11194. https://doi.org/10.1021/acs.analchem.0c01591.
